# Ultrasound localisation microscopy predicts neoadjuvant chemotherapy response in patients with breast cancer

**DOI:** 10.1016/j.ebiom.2026.106442

**Published:** 2026-08-24

**Authors:** Céline Porte, Matthias Kohlen, Thomas Lisson, Stefanie Dencks, Saskia von Stillfried, Zuzanna A. Magnuska, Brigitte S. Winkler, Anne Rix, Patrick Koczera, Peter Boor, Steven R. Talbot, Georg Schmitz, Elmar Stickeler, Fabian Kiessling

**Affiliations:** a Institute for Experimental Molecular Imaging, Uniklinik RWTH Aachen, Aachen, Germany; b Department of Gynecology and Obstetrics, Uniklinik RWTH Aachen, Aachen, Germany; c Center for Integrated Oncology (CIO) – Aachen, Düsseldorf (ABCD), Bonn, Köln, Germany; d Chair for Medical Engineering, Department of Electrical Engineering and Information Technology, Ruhr University Bochum, Bochum, Germany; e Institute of Pathology, Uniklinik RWTH Aachen, Aachen, Germany; f Institute for Laboratory Animal Science, Hannover Medical School, Hannover, Germany; g Fraunhofer Institute for Digital Medicine MEVIS, Aachen, Germany

**Keywords:** Ultrasound localisation microscopy, Super-resolution, Radiomics, Breast cancer, Neoadjuvant chemotherapy, Pathological complete response

## Abstract

**Background:**

Patients with breast cancer undergoing neoadjuvant chemotherapy mostly show insufficient therapy response and would highly benefit from an early prediction of pathological complete response (pCR). As vessels play a major role in drug delivery, ultrasound localisation microscopy (ULM) holds great promise as predictive tool by visualising vascular networks in high detail, at a resolution beyond the acoustic diffraction limit.

**Methods:**

In this prospective pilot study (NCT05445050), we examined patients with breast cancer undergoing neoadjuvant chemotherapy using a ULM algorithm designed for conventional clinical ultrasound systems. To generate a vascular fingerprint, we performed the most detailed radiomics ULM analysis to date, based on longitudinal data from 20 patients. Features were used to classify therapy response using logistic regression and the performance of ULM was compared to clinically available imaging methods.

**Findings:**

Compared to patients with non-pCR (n = 12), patients with pCR (n = 8) showed a more pronounced vascular network before therapy. Furthermore, their vessel coverage strongly changed following the first chemotherapy administration (26.97 [20.23–36.24] % vs. 6.00 [2.73–10.49] %), representing a promising biomarker for therapy prediction (p < 0.01, effect size: 2.21). ULM features allowed to correctly predict up to 85% (AUC: 0.85) of patient responses and already 75% (AUC: 0.73) before the start of therapy, thus outperforming tumour size measurements (accuracy up to 60%, AUC up to 0.53), B-mode images (accuracy up to 75%, AUC up to 0.76), maximum intensity projections of contrast-enhanced ultrasound (accuracy up to 75%; AUC up to 0.79), and histological CD31 staining (accuracy: 75%; AUC: 0.83).

**Interpretation:**

Our findings emphasise the importance of vascular assessment in predicting pCR in patients with breast cancer and promote the translation of ULM as powerful clinical tool with the potential to optimise neoadjuvant treatment schedules.

**Funding:**

This work was supported by the 10.13039/501100001659German Research Foundation.

**Research in context**

**Evidence before this study**

Patients with highly aggressive breast cancer typically receive a chemotherapy treatment before the surgical removal of their tumour. The goal of this treatment regimen is the complete disappearance of cancer cells in the tumour tissue (i.e., pathological complete response, pCR), which is associated with a particularly favourable prognosis. However, a large number of patients do not achieve pCR and would necessitate an adapted treatment, in turn requiring to prognosticate the patients’ therapy responses. Ultrasound localisation microscopy (ULM) holds great potential as a predictive tool as it allows to precisely visualise and characterise the microvasculature, which is expected to show the reaction of the tumour to chemotherapy particularly early on.

**Added value of this study**

We extensively quantified the vasculature of patients with breast cancer by computing a large set of parameters at different time points before and during therapy. Our analysis shows that, compared to patients with non-pCR, patients with pCR had a more pronounced and homogeneously distributed vessel network before therapy, which is favourable for drug delivery to tumours. We further observed that patients with pCR showed a stronger vascular reaction to chemotherapy very early on. The distinct vascular patterns of both groups allowed us to correctly predict the therapy response of 85% of patients and even 75% already prior to therapy. This appeared to outperform other diagnostic methods available in the clinics. Future larger-scale studies will include subgroup analyses, which are expected to improve therapy prediction even further.

**Implications of all the available evidence**

Predicting the patients’ therapy response would allow to individually escalate or deescalate their treatment from the beginning on, holding potential to tremendously improve treatment outcome. ULM could be a valuable tool for this purpose, while being cheap, applicable to standard clinical ultrasound devices, and thus suitable for clinical practice.

## Introduction

The vasculature plays a crucial role in cancer, where it is strongly involved in tumour growth, local invasion, and metastasis.^1^ In breast cancer, increased microvascular density, endothelial cell proliferation, and pro-angiogenic factor levels have been already reported in pre-invasive lesions such as ductal carcinoma in situ,^2^ suggesting that the angiogenic switch is one of the earliest changes in carcinogenesis.^3^ Accordingly, alterations in the tumour microvasculature tend to precede any measurable anatomical changes, both in tumour development^4^ and in response to anti-cancer treatments.^5^ The microvasculature may therefore serve as a promising biomarker of disease state, progression, and therapeutic efficacy.

Clinically, the vasculature of mammary lesions is primarily evaluated using dynamic contrast-enhanced magnetic resonance imaging (DCE-MRI). Despite providing valuable insight in perfusion,^6^ DCE-MRI is costly and assesses vascularisation only indirectly and semi-quantitatively,^7^ similar to contrast-enhanced mammography^8^ and dynamic contrast-enhanced ultrasound.^9^ Direct imaging of blood flow is currently only possible using Doppler ultrasound, but is restricted to macro- or near-microvessels because of its limited sensitivity to low blood flow, even when employing specialised microvascular flow imaging modes.^10^

Ultrasound localisation microscopy (ULM) is an emerging technique that enables the visualisation of microvascular networks and individual vessels in super-resolution, down to the capillary level,^11^^,^^12^ thus being of particular interest for breast cancer characterisation.^13^^,^^14^ By combining both morphological and functional information in unsurpassed spatial resolution, ULM can generate a detailed vascular profile of the tissue of interest that extends substantially beyond conventional perfusion measurements.^15^ Since its conceptual introduction in 2011,^16^^,^^17^ ULM has undergone significant technical improvements, enabling its use with clinical scanners and the conduction of in-human studies. To date, ULM has shown promising proof of concept in several fields^18^, ^19^, ^20^, ^21^ and has demonstrated initial evidence for differentiating benign and malignant breast masses^22^ and liver lesions,^23^ and for assessing changes during chemotherapy.^24^^,^^25^ Despite the growing interest in ULM, it remains an open question as to which applications ULM can address an unsolved clinical need and improve patient management.

In this context, we see a wide gap in the treatment of patients with high-risk breast cancer, which remains challenging. Nowadays, these patients are commonly subjected to neoadjuvant chemotherapy (NAC), a treatment regimen where systemic therapy, including anti-HER2 (human epidermal growth factor receptor 2) directed antibodies, checkpoint inhibitors, and chemotherapy, is applied before surgery.^26^ Patients showing pathological complete response (pCR) to NAC have a substantially higher overall and event-free survival,^27^ reaching 92% and 86% in patients with triple-negative breast cancer (TNBC) and pCR compared to only 58% and 50% in those without.^28^ However, a large number of patients do not achieve pCR^29^ and would strongly benefit from an early optimisation of their therapy. Currently, therapy response is only monitored by measuring tumour size,^30^ which allows to identify non-responders or patients with stable disease, but not to differentiate between patients with non-pCR and pCR.

**Fig. 1:**
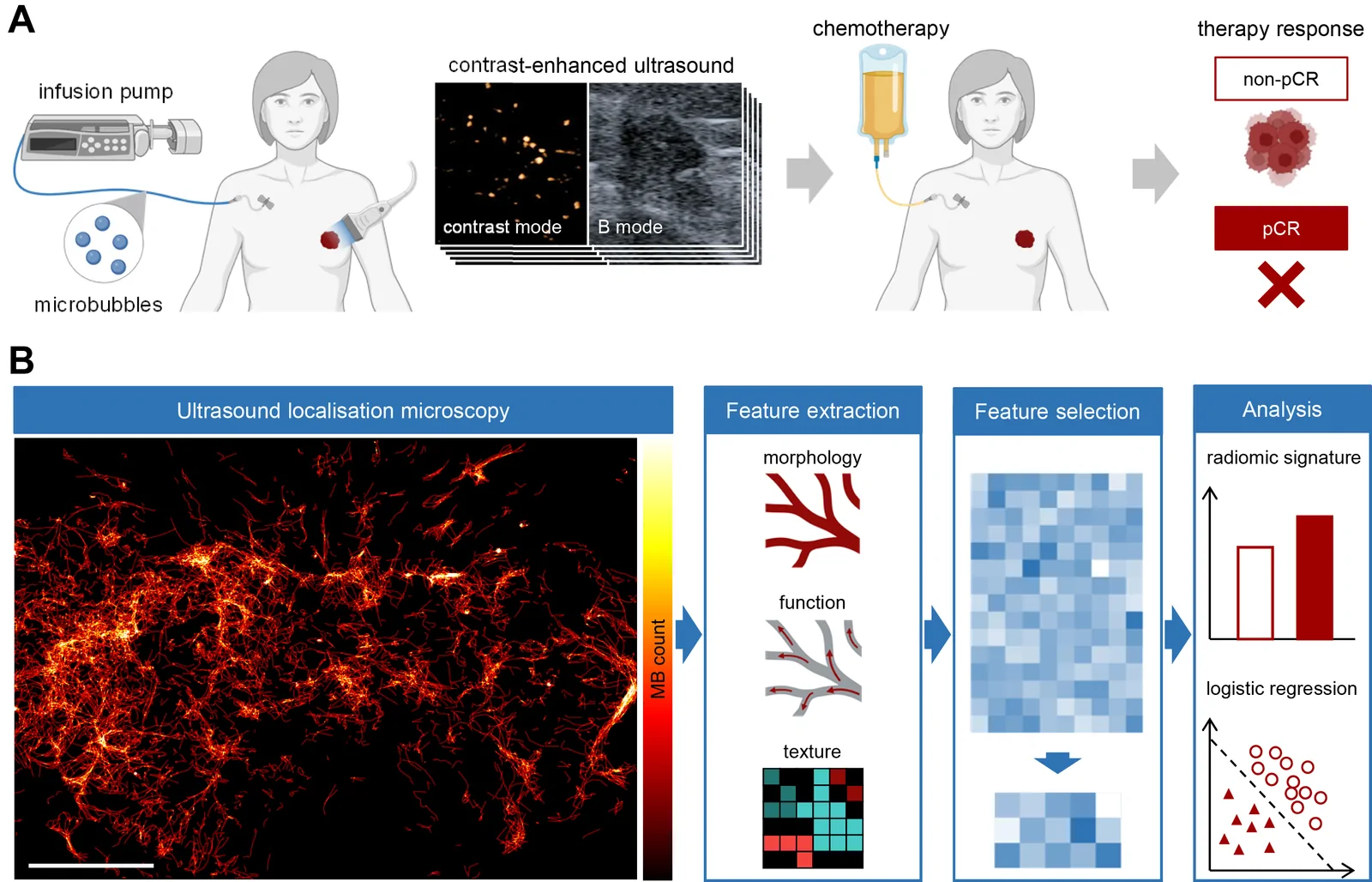
**Study overview. A** Before receiving neoadjuvant chemotherapy (NAC), patients underwent contrast-enhanced ultrasound (CEUS) in split-screen mode (contrast mode & B mode). After completion of all NAC cycles and surgical removal of the tumour bed, the patients' responses to NAC were determined to be a pathological complete response (pCR) in the absence of cancer cells in the breast resection specimen or a non-pCR in the opposite case. **B** Ultrasound localisation microscopy (ULM) images were calculated based on the recorded CEUS videos and analysed via a radiomics approach. For this purpose, morphological, functional, and texture features were extracted from the ULM data. After feature selection, these were used to obtain a radiomic signature of the tumour vasculature of both patients with non-pCR and pCR, which was in turn used to classify patient responses through logistic regression. Scale bar: 5 mm. Icons were created using BioRender.

Here, we propose ULM for improving both the preselection and monitoring of patients with breast cancer undergoing NAC. Within this prospective pilot study, we performed the most comprehensive radiomics analysis reported to date (Fig. 1), evaluating ULM-derived features both before and during early NAC. We identified significant differences in microvascular characteristics between patients with non-pCR and pCR, thereby allowing a better understanding of the connection between microvasculature and therapeutic efficacy. In addition, we employed logistic regression to predict therapy response before the start of NAC or very early during NAC and demonstrated that ULM outperformed conventional ultrasound imaging and histological CD31 analyses in terms of predictive performance.

## Methods

### Study design and approval

The clinical study (Supplementary Table S1) was conducted prospectively at the Department of Gynecology and Obstetrics of the Uniklinik RWTH Aachen, Germany. It was approved by the University's ethics committee (EK 351/19; the full study protocol is available in Supplementary Material) and registered at DRKS (DRKS00023954) and clinicaltrials.gov (NCT05445050). All patients gave their written consent to participate.

### Patient recruitment

Twenty-two female patients with histologically confirmed high-risk breast cancer scheduled for NAC were enrolled between September 2022 and April 2024. Because the study was exploratory, patient recruitment did not adhere to formal sample size planning. All eligible patients were consecutively included in the study. All treatment decisions were made independently of the study and in accordance with the national breast cancer guidelines.^31^ Exclusion criteria comprised age under 18 years, pregnancy, and contraindications to the contrast agent (Supplementary Table S1). In one patient, chemotherapy was discontinued, and another patient had a histological cancer subtype (invasive lobular) that differed from all others (no special type), which is why both were excluded.

### Patient examinations

The primary breast tumour of each patient was examined at the starting day of the first, second, and fourth cycle of chemotherapy. Each examination consisted of a tumour size and a contrast-enhanced ultrasound (CEUS) measurement with an Aplio 500 (Canon Medical Systems, Otawara, Japan) and a PLT 1005BT linear transducer at a frequency of 5 MHz.

#### Size measurement

Two B-mode images were recorded, at the largest tumour cross-section and perpendicular to it. The volume was calculated assuming an ellipsoid (see comparative methods).

#### CEUS measurement

Patients were instructed to breathe normally and avoid large-scale movements. MB injection and CEUS acquisition were conducted following a previously established protocol.^32^ Briefly, the patients were injected with 15 μl/kg of body weight (0.99 ± 0.36 ml) of SonoVue® (Bracco, Milan, Italy) via a chemotherapy port system connected to the common jugular vein using an infusion pump (Perfusor, B Braun, Melsungen, Germany) at 100 μl/s. Simultaneously to the start of injection, we recorded an ultrasound measurement in split-screen mode (B-mode and contrast mode), with a mechanical index of 0.07 and a frame rate of 10 Hz. The acquisition was distributed over three consecutive videos of 90 s each, resulting in a total of 2700 frames. These were processed sequentially, and the results were merged for the ULM analysis.

### ULM post-processing pipeline

All processing steps were performed using MATLAB (MathWorks, Natick, MA, USA), incorporating a C^++^ implementation for the tracking of MBs. As the ultrasound system does not allow to export RF or IQ data, we utilised the envelope data saved within DICOM files. Tumour segmentation and compensation for patient- and examiner motion were performed on the recorded CEUS data in B-mode, and MB detection and localisation on the data in contrast-mode.

#### Tumour segmentation

The frame showing the highest normalised cross-correlation (NCC) with all other frames during the main bolus (30–130 s after the start of MB injection) was determined as the reference frame. The tumour contour was manually outlined by a radiologist (F.K.; >20 years of experience), a resident doctor in gynaecology (M.K.; >4 years of experience), and a preclinical ultrasound expert (A.R.; >10 years of experience). To facilitate tumour segmentation and enable a better assessment of the extent of the tumour, annotators were provided with all acquired measurement data. In case of mismatches between annotators, a consensus decision was taken. Inter-observer variability was assessed using the dice similarity coefficient. All subsequent image processing steps were performed within a bounding box around the tumour (margin: 40 pixels (18/20 patients) or 80 pixels (2/20 patients)).

#### Motion compensation

Motion estimation was performed through non-rigid image registration with the reference frame using MATLAB's function *imregdemons*. Both B-mode and contrast-mode images were motion-corrected with the resulting displacement fields. Frames exhibiting an NCC<0.85 with the reference frame after motion compensation were considered out of plane and excluded.

#### MB detection and localisation

MBs were detected on motion compensated, thresholded (30/256) contrast images using MATLAB's function *imregionalmax*. To localise a single MB, a linearised Gaussian least-squares fit was applied to a patch of non-thresholded 7 × 7 pixels surrounding the detected maximum. Localisations where the MB width (estimated standard deviation) was greater than twice the median width of all fits were excluded.^33^

#### MB tracking

MBs were tracked using a Markov Chain Monte Carlo Data Association algorithm with an integrated motion model.^34^ All involved parameters were kept constant for all measurements.

#### Reduction of static noise

Tracks with less than 3 or more than 150 positions were excluded. Furthermore, we calculated a correction parameter *p*_corr_ for each pixel of the CEUS video, which is the percentage of frames where contrast signal was detected. Pixels with high *p*_corr_ are likely to represent false assignments of static noise. Thus, tracks containing MB positions detected within pixels with *p*_corr_>0.5 were excluded.

#### Visualisation

Four different parameter maps were computed using Bresenham's line algorithm, namely binary (0: no track, 1: one or more tracks), occurrence (number of tracks per pixel), flow velocity, and flow direction maps. The pixel size was set to Δx=Δz= 20 μm.

### ULM feature extraction

Feature extraction was performed in MATLAB based on raw track data, which contain the MB positions and velocities for each track, or on parameter maps. Both ULM reconstruction and feature extraction were performed blinded to clinical information and pCR status. Three different categories of features were determined: 30 morphological, 14 functional, and 2 texture features (Supplementary Table S2). The first category included the vessel coverage, i.e. the relative vessel area, as well as distances to the closest track, the variance of flow direction, track lengths, and track tortuosity.^35^ We also calculated a compensated variant of the vessel coverage (i.e., compensated vessel coverage; referred to as ${\hat{P}}_{v}$ in^36^), which accounts for differences between measurements and corresponds to the estimated value given complete vessel reconstruction.^36^ Functional features comprised MB velocity and track-wise velocity range. Furthermore, a fractal analysis was performed, yielding two texture features: the fractal dimension and its goodness-of-fit.^37^

The degree of reconstruction (DOR) achieved with ULM was determined by dividing the non-compensated by the compensated vessel coverage.

### Response assessment

The patients’ response to NAC was determined routinely, following the standard procedure of the breast centre at Uniklinik RWTH Aachen. Briefly, after surgical removal of the tumour bed and the sentinel lymph node, the resected tissue was analysed histologically. In the absence of invasive cancer in the breast resection specimen and the axillary lymph nodes, the patient was considered to show pCR.

### Comparative methods

To allow for a comparison with ULM, several clinically available methods were investigated, including conventional ultrasound and histology. Conventional ultrasound included a tumour size measurement, the B-mode reference frame used for ULM and a maximum intensity projection (MIP) of the motion-corrected contrast-mode video. The two latter images were resampled to a pixel width of 100 μm and discretised using a fixed grey-level bin size of 25. For the histological analysis, the patients’ core needle biopsy tissue, extracted routinely before NAC, was analysed for vessels using the endothelial cell marker CD31 (Supplementary methods; Supplementary Fig. S1). The tissue of one patient could not be analysed because the biopsy was performed externally. All comparative methods were quantified with radiomic features (Supplementary Table S3). Comparative methods and corresponding feature extraction were performed blinded to clinical information and pCR status.

### Delta radiomics

For measurements acquired at several time points (ULM, tumour size measurement, B-mode image, MIP) and for each extracted feature F, delta radiomic features were additionally calculated. For changes between two measurements, e.g., 1 and 2, these included:

$${\Delta F}^{\left(1,2\right)}=F^{(2)\;}-\;F^{\left(1\right)}$$

$$\left|{\Delta F}^{\left(1,2\right)}\right|=\left|F^{\left(2\right)}-F^{\left(1\right)}\right|$$

$$F_{\min}^{\left(1,2\right)}=\min\left(F^{\left(1\right)},F^{\left(2\right)}\right)$$

$$F_{\max}^{\left(1,2\right)}=\max\left(F^{\left(1\right)},F^{\left(2\right)}\right)$$

An overview of all analysed feature sets, including changes between measurements, is given in Supplementary Table S4.

### Statistical analysis

The distribution of individual features was evaluated using the Shapiro–Wilk test, histograms, and Q–Q plots, which indicated deviations from normality. Accordingly, parameters are reported as median and interquartile range (IQR). Differences between groups (non-pCR vs. pCR) are illustrated with boxplots where the middle line represents the median, the box the IQR, the whiskers the outermost values of non-outliers, and the scatter points outliers. Effect sizes were quantified using Hedge's g, which is appropriate for small sample sizes, with bootstrapped confidence intervals to account for non-normality. For inferential statistics, continuous variables were compared between patients using two-sided Wilcoxon rank sum tests and across time points using two-sided Wilcoxon signed-rank tests. Categorical variables were analysed using Fisher's exact test. Statistical significance was assumed if p < 0.05. To account for multiple testing, Benjamini-Hochberg correction was applied to control the false discovery rate (FDR) at 5 or 10% (Supplementary Fig. S2).

While ultrasound measurements, ULM post-processing, and histological staining were successfully performed for all patients, one patient showed no detectable perfused vessels within the tumour region in one of the CEUS measurements. This prevented the calculation of certain ULM features, which were handled as missing values and excluded from feature-specific analyses, while the measurement was retained in all classification and performance analyses.

### Principal component analysis

A principal component analysis (PCA) was performed to evaluate the ability of ULM or comparative methods to separate patients with pCR and non-pCR in an unsupervised manner. Therefore, features were first normalised using z-score. Non-informative (quasi-)constant features with an IQR of zero before normalisation were removed. Feature correlation was determined using Spearman's ρ and, for each feature pair with |ρ| > 0.9, the feature with the lower predictive performance (i.e., the lower area under the receiver operating curve, AUC) was removed. If both features exhibited equal AUC, the feature with the lower effect size was removed. The remaining features were used for the PCA. Results were evaluated based on scatter plots of the first four principal components.

### Predictive performance

The predictive performance of each imaging method was assessed via logistic regression with leave-one-out cross-validation (LOOCV) (Supplementary Fig. S3). Feature selection was embedded within the LOOCV framework and included feature normalisation as well as the exclusion of non-informative or highly correlated features. From the remaining feature set, features with the highest AUC were selected. Logistic regression was chosen as a low-complexity classification model. Predicted probabilities ≥0.5 were classified as pCR. The model configuration was optimised via grid search (Supplementary Table S5), exploring different types of feature normalisation, feature correlation reduction, numbers of selected features, and model regularisations. The model with the highest predictive accuracy across all evaluated model configurations was selected as the final model. If several models achieved equal accuracy, the model with the highest AUC was chosen. Model performance was evaluated using predictive accuracy, AUC, sensitivity, and specificity. Additionally, the AUC of the final ULM model was compared with those of the comparative methods using DeLong's test. To assess the robustness of feature selection, feature selection frequencies were determined for an exemplary configuration that involved additional LASSO-based feature selection (Supplementary Fig. S4).

The predictive performance of individual features was quantified via the AUC, calculated with MATLAB's *perfcurve* function, with therapy response (pCR vs. non-pCR) as the binary class label and the feature values as predictor scores. Corresponding 95% confidence intervals were estimated by non-parametric bootstrapping using 5000 resamples. Predictive accuracy was assessed through logistic regression with LOOCV.

### Role of the funding source

The funding source had no role in study design, data collection, data analysis, data interpretation, or writing of this manuscript.

## Results

### Patient characteristics

Among the twenty analysed patients (Supplementary Fig. S5), all major breast cancer subtypes were represented: nine luminal, hormone receptor (HR)-positive, two HER2-positive, and nine TNBCs (Table 1, Supplementary Table S6). Based on histopathological characteristics, patients received either weekly paclitaxel and carboplatin, paclitaxel alone, or biweekly epirubicin and cyclophosphamide. Eight patients achieved pCR while twelve did not. None exhibited stable or progressive disease. The observed pCR rates were consistent with subtype-specific expectations,^38^ reaching 11% for HR-positive, 50% for HER2-positive, and 67% for TNBC. In the case of TNBC, the pCR rate was higher than typically reported, but was in line with studies that demonstrated better outcome when administering platinum-based chemotherapeutics as carboplatin^39^ and the immune checkpoint inhibitor pembrolizumab.^40^

**Table 1:** Baseline clinical characteristics.

Characteristic	Non-pCR (n = 12) n (%)	pCR (n = 8) n (%)	All (n = 20) n (%)	p-value
**General**				
Sex				
Female	12 (100)	8 (100)	20 (100)	1.00
Age (years)^a^	55.0 (49.5–67.5)	46.5 (34.5–56.0)	52.5 (41.5–61.5)	0.07
**Cancer diagnosis**				
Tumour type				0.05
Hormone receptor positive	8 (67)	1 (12.5)	9 (45)	
HER2 receptor positive	1 (8)	1 (12.5)	2 (10)	
Triple negative	3 (25)	6 (75)	9 (45)	
Histological subtype				1.00
No special type	12 (100)	8 (100)	20 (100)	
Oestrogen receptor (%)^a^	100 (47.5–100)	0 (0–100)	47.5 (0–100)	<0.01
Progesterone receptor (%)^a^	6 (0–60)	0.5 (0–1.5)	1 (0–42.5)	0.18
HER2 receptor				1.00
0	4 (33)	3 (38)	7 (35)	
0+	1 (8)	0 (0)	1 (5)	
1+	3 (25)	2 (25)	5 (25)	
2+/FISH-	3 (25)	2 (25)	5 (25)	
3+	1 (8)	1 (13)	2 (10)	
Ki67 (%)^a^	43 (35–50)	72.5 (51–80)	49 (40–73)	0.03
Grade				1.00
G2	4 (33)	2 (25)	6 (30)	
G3	8 (67)	6 (75)	14 (70)	
**Treatment**				
Neoadjuvant chemotherapy^b^				0.06
Paclitaxel	1 (8)	0 (0)	1 (5)	
Carboplatin + Paclitaxel	3 (25)	6 (75)	9 (45)	
Epirubicin + Cyclophosphamide	8 (67)	2 (25)	10 (50)	
Neoadjuvant chemotherapy cycle^b^				0.17
Weekly	4 (33)	6 (75)	10 (50)	
Biweekly	8 (67)	2 (25)	10 (50)	
Additional neoadjuvant therapy				0.01
Pembrolizumab (every 3 weeks)	1 (8)	5 (63)	6 (30)	
Pertuzumab (every 3 weeks)	1 (8)	1 (13)	2 (10)	
Trastuzumab (every 3 weeks)	1 (8)	1 (13)	2 (10)	
**Therapy response**				
Primary tumour remaining				
ypT0	1 (8)	8 (100)	9 (45)	
ypT1a	2 (17)	0 (0)	2 (10)	
ypT1b	2 (17)	0 (0)	2 (10)	
ypT1c	2 (17)	0 (0)	2 (10)	
ypT1mi	1 (8)	0 (0)	1 (5)	
ypT2	3 (25)	0 (0)	3 (15)	
ypT3	1 (8)	0 (0)	1 (5)	
Lymph node remaining				
ypN0	7 (58)	7 (88)	14 (70)	
ypN0 (i+)	1 (8)	1 (13)	2 (10)	
ypN1a	4 (33)	0 (0)	4 (20)	

Differences between patients achieving pCR and non-pCR were assessed using Wilcoxon rank-sum test for continuous variables and Fisher's exact test for categorical variables.

a Characterised as median (interquartile range (IQR)).

b Referring to the first three cycles.

### ULM proved practical in clinical routine

All twenty-two examined patients were successfully imaged and processed for ULM using a standard clinical ultrasound system with a frame rate of 10 Hz. No adverse events occurred. Clinical practicability was further ensured by restricting the acquisition time to 4.5 min, while not requiring the patients to hold their breath at any time. This still allowed us to process the large majority of acquired frames (median: 1883 (70%), IQR: 1149 (43%)–2549 (94%)). By using a previously optimised MB injection protocol^32^ in combination with an advanced tracking algorithm enabling ULM imaging at low frame rates,^24^^,^^34^^,^^41^ we obtained ULM parameter maps with an estimated median DOR of 36% (IQR 24–46%), which allowed for an adequate evaluation of the vascular architecture.

### Patients with pCR had a more pronounced vascular network before therapy

**Fig. 2:**
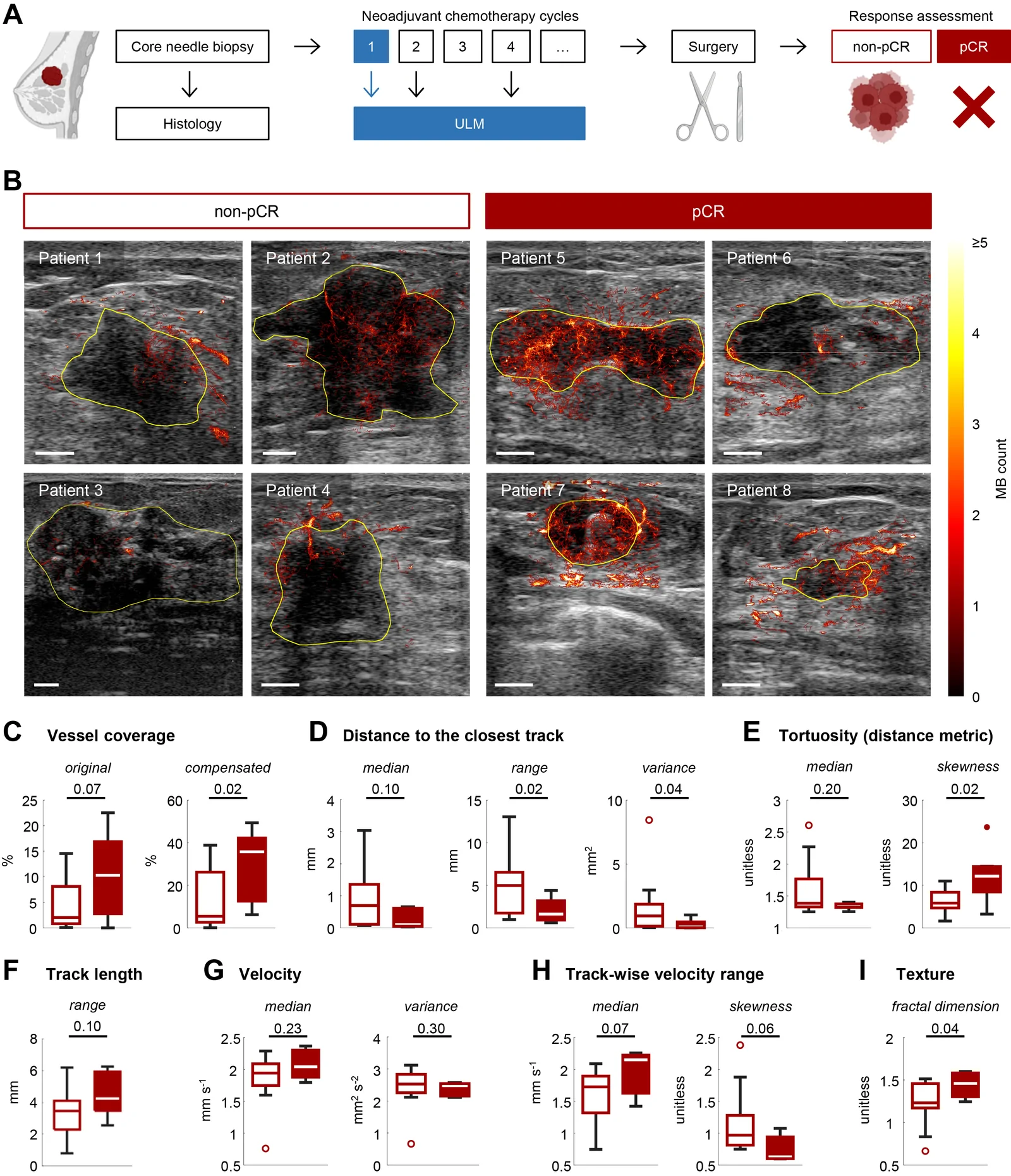
**Vascular differences in patients with non-pCR vs. pCR before therapy. A** Analysis of ULM data acquired right before the first NAC cycle. **B** ULM occurrence maps of primary tumours (outlined in yellow) overlaid on B-mode reference frames. Representative images are shown both for patients with non-pCR (left) and pCR (right). Scale bars: 5 mm. **C–I** Quantitative ULM features of patients with pCR (filled box; n_pCR_ = 8 (original vessel coverage) or 7 (others)∗) and without (unfilled box; n_non-pCR_ = 12), shown as boxplots with median and interquartile range (IQR). p-values were determined via a two-sided Wilcoxon rank sum test. ∗Patient 16 showed no perfused vessels within the tumour at baseline, preventing the computation of some features. Icons were created using BioRender.

Before therapy (Fig. 2A), primary tumours of patients with non-pCR tended to be poorly vascularised, with vessels mostly confined to specific tumour regions, resulting in large non-perfused areas (Fig. 2B). In contrast, tumours of patients with pCR generally displayed a considerably more pronounced and homogeneously distributed vascular network. Despite these noticeable differences, some patients deviated from this trend, for example patients 6 and 16 (Supplementary Fig. S6), who both showed very low vascularisation while achieving pCR.

Tumour vascularisation was extensively quantified within manually delineated tumour ROIs, which showed good agreement among the three annotators (Supplementary Table S7). Among ULM features (Fig. 2C–I; Supplementary Table S8), vessel coverage tended to be higher in patients with pCR (nominal p = 0.07 (two-sided Wilcoxon rank sum test), effect size: Hedges’ g = 0.89), and compensated vessel coverage was significantly higher (p = 0.02, g = 1.08), with a median (IQR) of 35.7% (12.6–42.2%) compared to 5.5% (2.7–26.2%) in patients with non-pCR. To characterise the spatial distribution of vessels, we further analysed distances to the closest track, which highlight non-perfused regions (Supplementary Fig. S7). These showed lower mean (p = 0.18, g = −0.94), median (p = 0.10, g = −0.87), range (p = 0.02, g = −0.93), and variance (p = 0.04, g = −0.67) in patients with pCR, indicating a more homogeneous vessel network with fewer hypovascular regions. Regarding vessel tortuosity, the distance metric (DM) demonstrated significantly higher skewness (p = 0.02, g = 1.25) and kurtosis (p = 0.03, g = 1.22) in patients with pCR, indicating distributions dominated by low tortuosity values. This suggests that patients with non-pCR generally exhibited a larger proportion of highly tortuous vessels. In line with this, the sum-of-angles metric (SOAM), which emphasises angle magnitude, indicated trends toward lower mean (p = 0.20, g = −0.73), variance (p = 0.12, g = −0.79) and IQR (p = 0.14, g = −0.76) in the pCR group. Furthermore the track length tended to be higher in patients with pCR (mean: p = 0.17, g = 0.75; median: p = 0.14, g = 0.76; range: p = 0.10, g = 0.79). The variance of flow direction did not differ between groups, which could have implied a more structured or directed vessel architecture in one of the groups otherwise.

Regarding functional features, both patient groups exhibited comparable flow velocity profiles. Additionally, the range of velocities within individual tracks showed a higher median (p = 0.07, g = 0.88), lower kurtosis (p = 0.04, g = −0.70) and lower skewness (p = 0.06, g = −0.88) in patients with pCR. Therefore, MBs tended to exhibit larger velocity differences along their trajectory, indicating a more hierarchical vascular network ranging from larger vessels to capillaries.

Among texture features, both the fractal dimension and its goodness of fit $R^{2}$ were significantly higher in patients with pCR (p = 0.04, g = 0.90 and p = 0.02, g = 0.46). A higher fractal dimension implies a higher texture complexity and thus, a denser and probably highly branched vascular network, while $R^{2}$ indicates a closer resemblance to a fractal, meaning that the network is similarly developed across all scales, supporting a well-defined vessel hierarchy.

### Patients with pCR showed stronger vascular changes in response to NAC

**Fig. 3:**
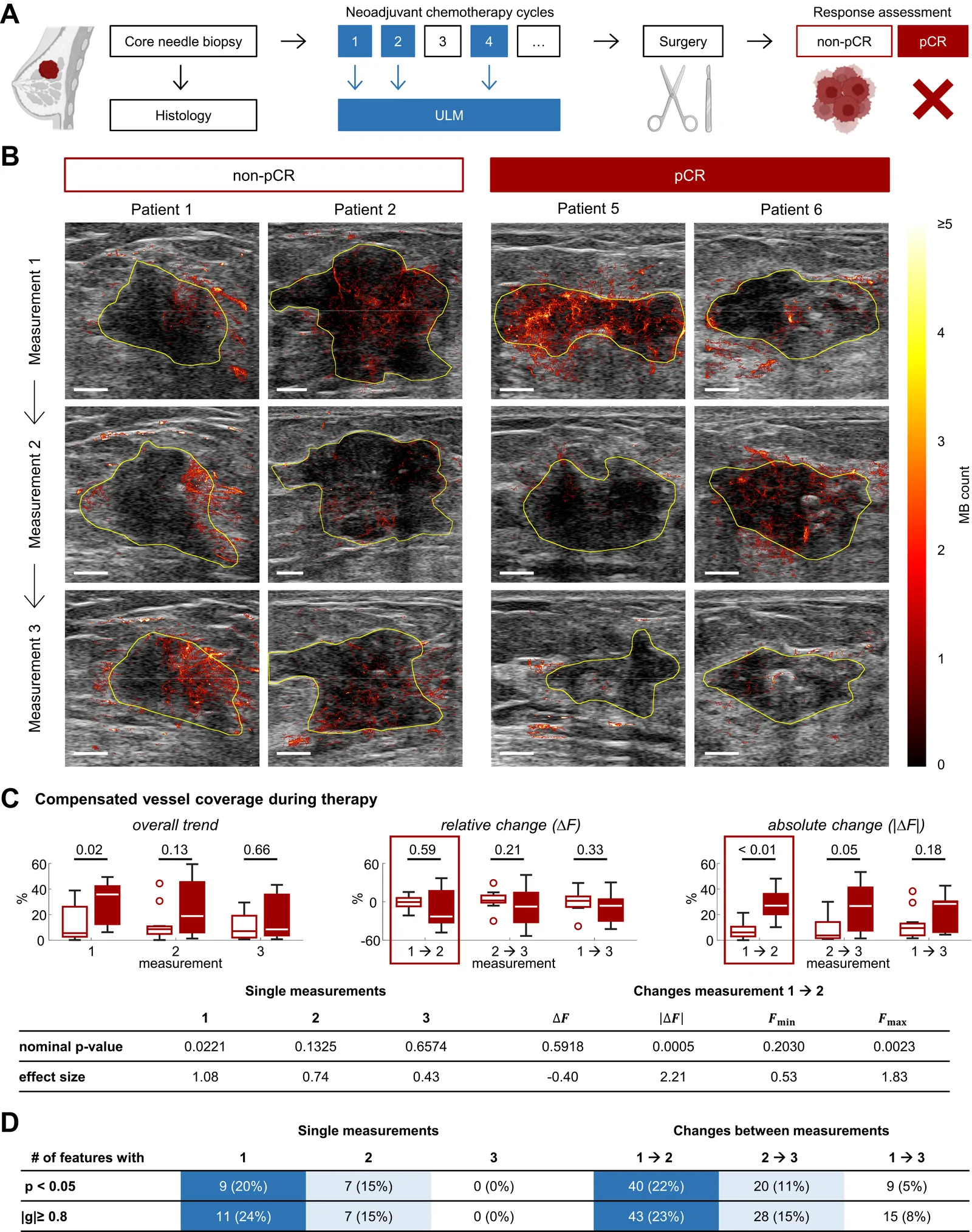
**Vascular changes during therapy in patients with non-pCR vs. pCR. A** Analysis of ULM data acquired right before the first, second, and fourth NAC cycle. **B** ULM occurrence maps of primary tumours (outlined in yellow) during therapy (top to bottom) overlaid on B-mode reference frames. Representative images are shown both for patients with non-pCR (left) and pCR (right). Scale bars: 5 mm. **C** Compensated vessel coverage during therapy (n_non-pCR_ = 11 or 12∗, n_pCR_ = 7 or 8∗), shown as boxplots with median and IQR. Next to the overall trend (left), the relative change (middle) and absolute change (right) between measurements are shown. p-values were determined via a two-sided Wilcoxon rank sum test. **D** Table showing the number of ULM features with significant differences (nominal p < 0.05) or high effect size (|Hedges' g|>0.8) between patients with non-pCR (n_non-pCR_ = 11 or 12∗) and pCR (n_pCR_ = 7 or 8∗), across time points and during therapy (delta radiomics). Statistical significance was determined via a two-sided Wilcoxon rank sum test. ∗Patients 16 and 18 showed no perfused vessels within the tumour during the first and third measurement, respectively, preventing the computation of some features. Icons were created using BioRender.

During early NAC (Fig. 3A), patients with non-pCR exhibited only a slight decrease in vascularisation, sometimes even a slight increase, but rarely showed distinct changes (Fig. 3B). In contrast, the vasculature notably receded already after the first cycle in patients with pCR. However, tumours of patients with pCR that were poorly perfused at baseline first showed an increase in vascularisation, for example in patients 6 and 16 (Supplementary Fig. S6).

ULM quantification confirmed that the compensated vessel coverage remained stable over time in patients with non-pCR (Fig. 3C). In contrast, patients with pCR exhibited a notable decrease, with median (IQR) values declining from 35.7% (12.6–42.2%) at baseline to 8.5% (3.7–35.6%) by the third measurement. While the relative changes in vascularisation between measurements of individual patients only indicated a negligible trend toward a stronger reduction in patients with pCR (p = 0.59, g = −0.40), significant differences between both groups became apparent when considering absolute changes in compensated vessel coverage (p < 0.01). Notably, this feature exhibited a large effect size (g = 2.21), indicating substantially larger vascular changes in patients with pCR in response to chemotherapy compared to patients with non-pCR. Additionally, the absolute change in compensated vessel coverage showed strong individual predictive performance, reaching a cross-validated predictive accuracy of 84% (sensitivity: 75%, specificity: 91%) and an AUC of 0.95. Compared with patients with hormone receptor-positive breast cancer, patients with TNBC exhibited even higher changes (22.21 (13.40–31.94) % vs. 6. 13 (5.70–13.40) %; Supplementary Fig. S8).

These longitudinal variations in radiomic features, referred to as delta radiomics,^42^ were analysed across all ULM-derived features (Supplementary Tables S9 and S10). Metrics included both relative (Δ*F*) and absolute changes (|Δ*F*|) between measurements, as well as minima (*F*_min_) and maxima (*F*_max_). As shown in Fig. 3D, most differences between patients with pCR and non-pCR were present among changes between measurements 1 and 2, followed by features derived from baseline. In contrast, later measurements and delta radiomic features of measurements 2–3 and 1–3 played a comparatively minor role in differentiating both patient groups. Similar findings were obtained after applying the Benjamini-Hochberg procedure to control the FDR at 5 and 10%. After correction for multiple testing, only delta radiomics features of measurements 1 to 2 remained statistically significant, including the absolute change in compensated vessel coverage, among others (Table 2).

**Table 2:** ULM features considered significant after Benjamini-Hochberg correction.

Feature	p	pCR	Non-pCR	Effect size	Accuracy (%)	AUC
Measurement 1 → 2						
Compensated vessel coverage in % (|Δ*F*|)	**0.0005**	**28.39 ± 12.52**	**7.50 ± 6.34**	**2.21 [0.48, 3.43]**	**84**	**0.95 [0.71, 1.00]**
Velocity range—skewness (*F*_min_)	**0.0014**	**0.66 ± 0.11**	**1.14 ± 0.51**	**−1.13 [−1.67, 0.70]**	**85**	**0.94 [0.71, 1.00]**
Tortuosity (SOAM)—mean in rad/mm (*F*_min_)	**0.0018**	**6.07 ± 0.40**	**7.93 ± 1.85**	**−1.21 [−1.99, −0.29]**	**90**	**0.93 [0.65, 1.00]**
Track length—median in mm (*F*_max_)	**0.0018**	**0.57 ± 0.04**	**0.44 ± 0.09**	**1.49 [0.81, 2.19]**	**80**	**0.93 [0.67, 1.00]**
Distances—skewness (|Δ*F*|)	**0.0018**	**1.41 ± 0.52**	**0.58 ± 0.54**	**1.49 [0.15, 2.88]**	**68**	**0.92 [0.62, 1.00]**
Compensated vessel coverage in % (*F*_max_)	**0.0023**	**41.71 ± 11.45**	**16.06 ± 14.57**	**1.83 [0.71, 3.42]**	**80**	**0.92 [0.69, 0.99]**
Tortuosity (SOAM)—median in rad/mm (*F*_min_)	0.0049	3.92 ± 0.56	5.80 ± 2.09	−1.08 [−1.60, −0.33]	75	0.89 [0.60, 0.98]

Given for an FDR of 5% (highlighted in bold) or 10%. From left to right: feature name, nominal p-value, mean ± standard deviation of patients with pCR and non-pCR, effect size (Hedges' g with bootstrapped 95% confidence interval), cross-validated predictive accuracy, AUC with bootstrapped 95% confidence interval.

### Classification based on ULM-derived features accurately predicts pCR

**Fig. 4:**
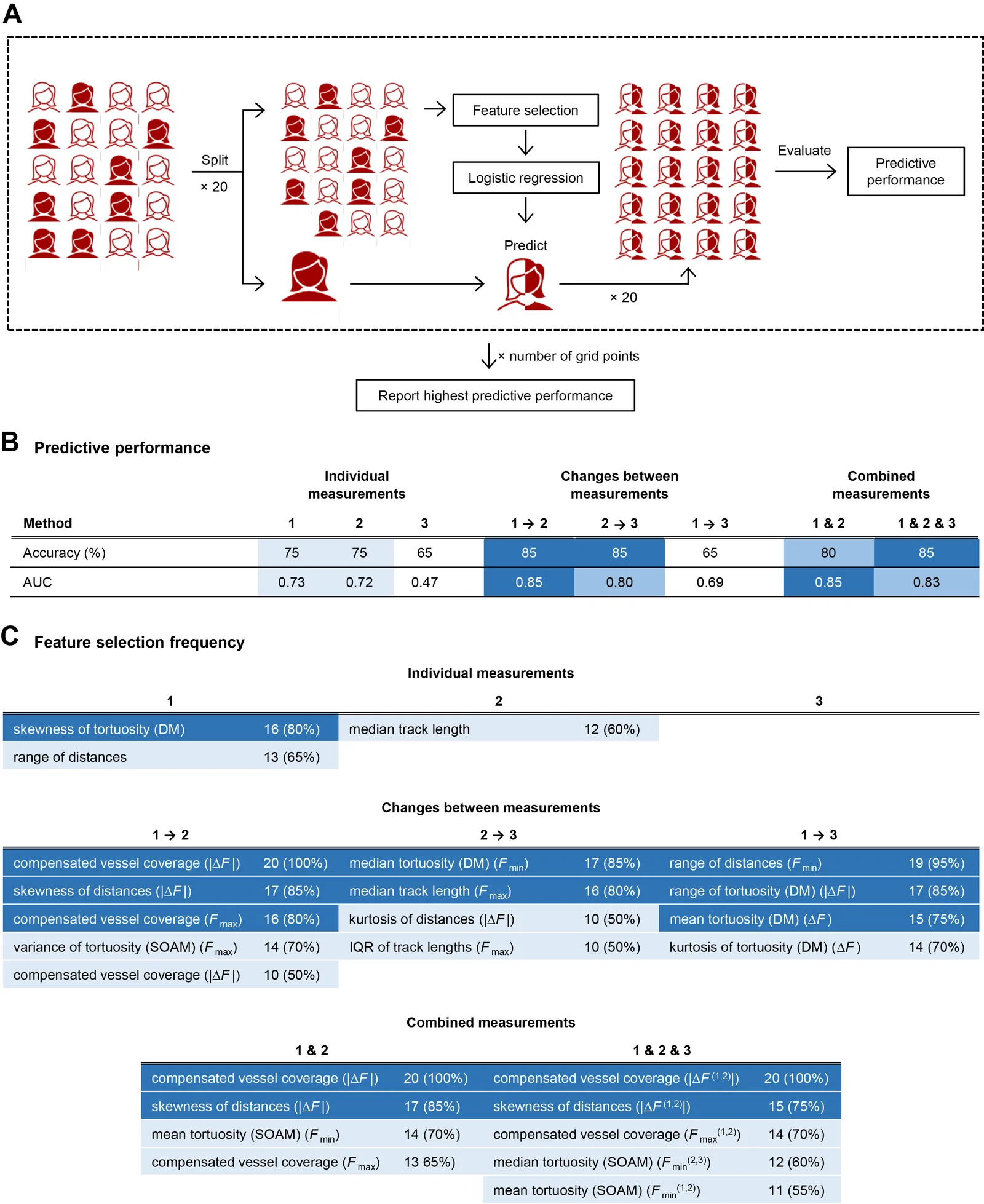
**Classification of patient response in non-pCR and pCR using ULM features. A** Classification workflow: Model performance was evaluated based on LOOCV. Therefore, patients were split into a training group (n = 19) and a validation group (n = 1). Feature selection and classification via logistic regression were performed on the training group. The model was then used to predict the remaining patient. This procedure was repeated 20 times, after which model performance was evaluated based on predictive accuracy and AUC. The model design was optimised via a grid search, and the best predictive performances were reported. **B** Classification results for individual measurements, changes between measurements, and the combination of several measurements. **C** Features with selection frequency ≥50% among the different measurements. The selection frequency of a feature was determined as the percentage of times it was selected for classification among the 20 folds of LOOCV.

Predictive performance was evaluated using logistic regression embedded within an LOOCV framework, combined with a grid search to identify optimal model parameters (Fig. 4A). The highest predictive accuracies (85%) and AUC values (0.85) were achieved based on changes between the first and second measurements (Fig. 4B). When only considering the first measurement performed before the start of therapy, ULM still achieved a predictive accuracy of 75% and an AUC of 0.73. While the second measurement yielded comparable results, accuracy decreased for the third measurement.

The stability of feature selection was assessed through a resampling-based sensitivity analysis (Fig. 4C; Supplementary Tables S11 and S12). Classification based on the first measurement involved one feature with feature selection frequency p_f_ ≥ 50% and one with p_f_ ≥ 75%, while measurements 2 and 3 showed comparatively lower selection stability. The highest selection frequencies were obtained for features derived from changes between measurements 1 and 2 (two features with p_f_ ≥ 0.50 and three with p_f_ ≥ 0.75). In line with previous observations, the change in compensated vessel coverage between measurements 1 and 2 was selected in all LOOCV folds (p_f_ = 100%).

### ULM can outperform alternative clinically available methods

**Fig. 5:**
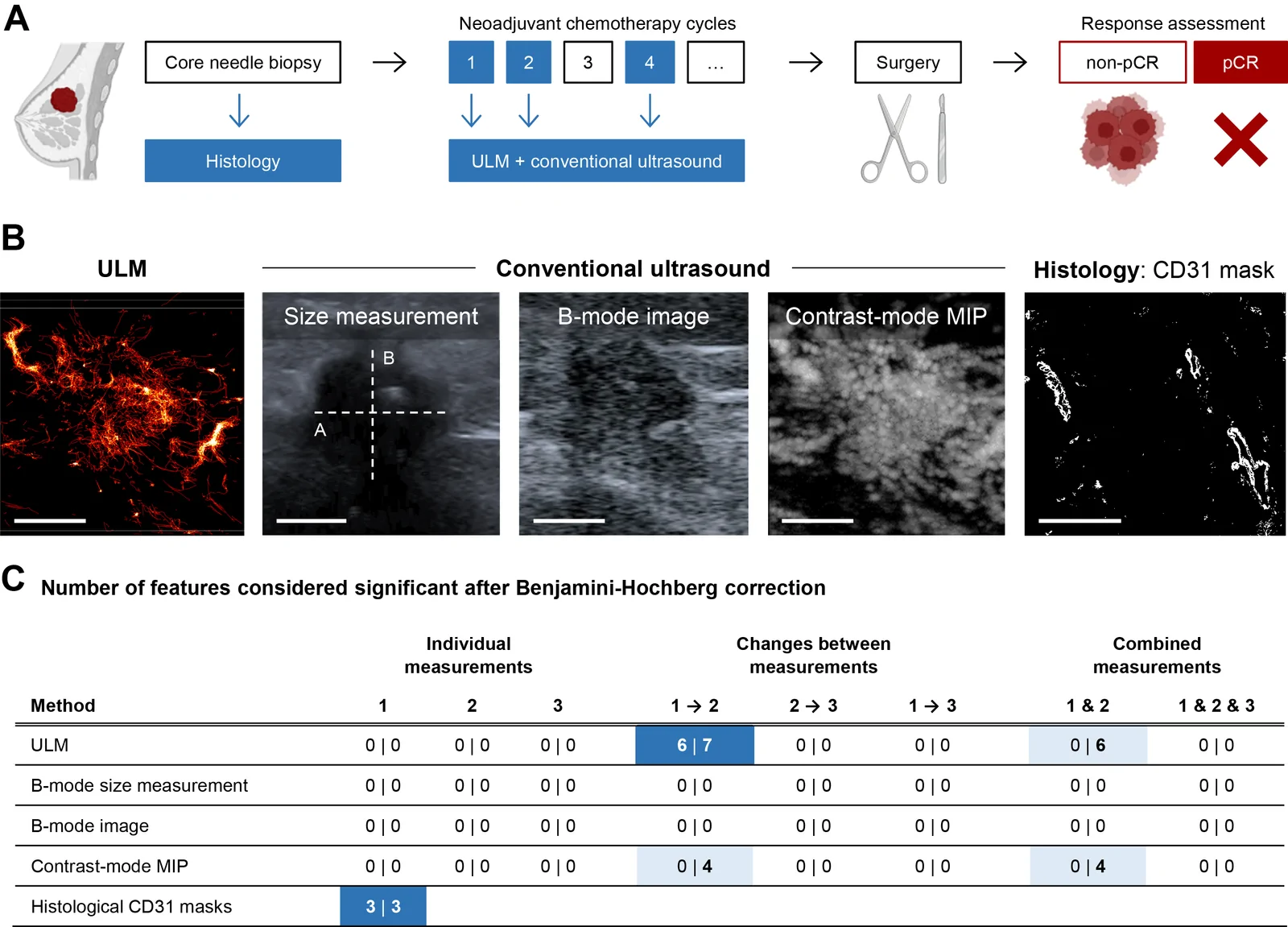
**Comparison of ULM with clinically available methods. A** Analysis of pre-therapy histology data as well as ULM and conventional ultrasound data acquired right before the first, second, and fourth NAC cycles. **B** Exemplary images of all methods. Scale bars: 5 mm (ULM and conventional ultrasound), 100 μm (histology). **C** Number of features considered significant after Benjamini-Hochberg correction to control the FDR at 5% (left value) or 10% (right value) (ultrasound: n_non-pCR_ = 12, n_pCR_ = 8; histology: n_non-pCR_ = 12, n_pCR_ = 7; the biopsy tissue of patient 14 was not available). Icons were created using BioRender.

We further compared the performance of ULM with conventional ultrasound and histology (Fig. 5A and B (Supplementary Tables S13–S23). Across all three time points, neither tumour volumes nor features extracted from B-mode images notably differed between patients with non-pCR and pCR. In contrast, MIPs revealed differences in changes between the first and second measurements. Histological CD31 masks yielded several features with large effect sizes and significant nominal p-values. After correction for multiple testing and controlling the FDR at 5%, only features derived from ULM (n = 6) and histological CD31 masks (n = 3) remained significant (Fig. 5C; Supplementary Table S24). At an FDR threshold of 10%, four significant features were identified for contrast-mode MIPs, whereas none were identified for the remaining methods.

Similar trends were observed concerning predictive performance. Before NAC, the highest accuracy was achieved with ULM and histological CD31 masks (75%), the latter showing additionally the highest AUC (0.83). When including longitudinal information, the performance of ULM notably increased, achieving the best results among all evaluated methods (accuracy 85%, AUC 0.85; Table 3). Notably, contrast-mode MIPs yielded higher AUC values than conventional B-mode images, suggesting that vascular information may be more informative than purely morphological tissue characteristics. B-mode size measurements, the only therapy monitoring approach currently performed in clinical practice, did not allow for any differentiation between patients with pCR and non-pCR.

**Table 3:** Predictive performance of ULM compared with clinically available methods.

Method	Individual measurements			Changes between measurements			Combined measurements	
	1	2	3	1 → 2	2 → 3	1 → 3	1 & 2	1 & 2 & 3
Accuracy (%)								
ULM	***75***	***75***	65	**85**	**85**	65	*80*	**85**
B-mode size measurement	60	60	60	60	60	60	60	60
B-mode image	60	60	60	65	***75***	60	60	***75***
Contrast-mode MIP	65	60	65	***75***	***75***	65	***75***	***75***
Histological CD31 masks	***75***							
AUC								
ULM	***0.73***	***0.72***	0.47	**0.85**	*0.80*	0.69	**0.85**	*0.83*
B-mode size measurement	0.31	0.35	0.53	0.21	0.38	0.32	0.34	0.29
B-mode image	0.00	0.26	0.13	0.13	***0.75***	0.21	0.25	0.76
Contrast-mode MIP	0.57	0.13	0.44	***0.79***	0.63	0.54	0.77	0.73
Histological CD31 masks	*0.83*							
Sensitivity (%)								
ULM	50	63	25	***75***	**88**	38	***75***	***75***
B-mode size measurement	0	0	0	0	0	0	0	0
B-mode image	0	0	0	13	***75***	0	0	***75***
Contrast-mode MIP	38	13	25	50	63	38	50	50
Histological CD31 masks	38							
Specificity (%)								
ULM	**92**	*83*	**92**	**92**	*83*	*83*	*83*	**92**
B-mode size measurement	**100**	**100**	**100**	**100**	**100**	**100**	**100**	**100**
B-mode image	**100**	**100**	**100**	**100**	**75**	**100**	**100**	***75***
Contrast-mode MIP	*83*	**92**	**92**	**92**	*83*	*83*	**92**	**92**
Histological CD31 masks	**100**							
p-value of DeLong's test—AUC of ULM compared to								
B-mode size measurement	0.05	0.02	0.77	<0.01	<0.01	0.10	0.01	<0.01
B-mode image	<0.01	0.02	0.05	<0.01	0.90	<0.01	<0.01	0.81
Contrast-mode MIP	0.25	<0.01	0.90	0.47	0.31	0.33	0.37	0.33
Histological CD31 masks^a^	0.55	0.59	0.04	0.93	0.88	0.28	0.93	1.00

Predictive performance was quantified via accuracy, AUC, sensitivity, and specificity of each method's final model. Additionally, the AUC of the final ULM model was compared with those of the comparative methods using DeLong's test.

a ULM-derived models were compared with the histological CD31 masks obtained before the start of NAC.

Conventional ultrasound-based methods showed lower feature selection frequencies compared with ULM, except for contrast-mode MIPs when combining the first two or three measurements (Supplementary Table S12). Histology-based classification involved two features with p_f_ ≥ 75% and one with p_f_ ≥ 50%, indicating consistent model behaviour and feature tendencies, similarly to ULM. An overview of all selected features is provided in Supplementary Tables S11 and S25–S28.

PCA did not reveal clear separation between both patient groups for any method.

## Discussion

In this study, we evaluated ULM for predicting the response of patients with breast cancer to NAC. Among others, we showed that pCR was linked to distinct vascular characteristics of the primary tumour prior to treatment. Compared to patients with non-pCR, patients with pCR exhibited a more pronounced and homogeneously distributed vascular network, reflected in higher vessel coverage, shorter and less varied distances to the closest track, and a higher fractal dimension. Furthermore, they tended toward a more hierarchical vascular organisation, as indicated by a closer resemblance to a fractal, a trend toward longer tracks, and higher track-wise velocity ranges, which imply a broader spectrum of vessel diameters. In contrast, patients with non-pCR displayed larger non-perfused regions, impeding drug penetration and increasing the risk of intratumoral hypoxia, which is a driver of tumour aggressiveness, metastatic potential, and treatment resistance.^43^ Patients with non-pCR further exhibited higher vessel tortuosity, which can cause pathological blood flow^44^ and is linked to tumour malignancy.^45^, ^46^, ^47^ Blood flow, in turn, did not seem to be impacted. Taken together, our findings suggest the presence of a more dense and functional vascular architecture in patients with pCR, enabling efficient intratumoral blood supply and better drug delivery, thus supporting therapeutic efficacy. This link is even exploited in the context of tumour priming, where vascularisation is enhanced before therapy to improve drug accumulation.^48^

Next, we investigated differences in the early vascular response to NAC. Notably, response patterns were heterogeneous among patients, some showing a progressive decrease in vascularisation, while others exhibited an increase at first, as already observed previously.^24^^,^^25^ In general, vascularisation is expected to decrease in response to NAC over the course of therapy, as chemotherapeutic agents induce cancer cell death, besides killing some endothelial cells directly, thereby reducing the expression of angiogenic factors. We hypothesise that, in some patients, vessel functionality might be impaired by high intratumoral pressure before therapy. Patient 6, for example, had particularly dense breast tissue (ACR density C–D), and patient 16 showed high cancer cell proliferation (Ki67 of 90%), which could both promote high intratumoral pressure.^49^ In such cases, NAC-induced cancer cell death could reduce pressure, enabling collapsed vessels to reopen. Additionally, NAC can also cause the vessels to draw closer as the cancer cell compartment shrinks due to death and degradation of cancer cells.^50^ Despite the broad range of observed responses and in contrast to patients with non-pCR, vascularisation tended to decrease over time in patients with pCR. Most notably, patients with pCR demonstrated a significantly higher absolute change in vessel coverage in response to the first chemotherapy administration. From a mechanistic perspective, this is line with the assumption that better therapeutic efficacy, resulting in a higher percentage of cancer cell death, should provoke a stronger vascular response, no matter if caused by a pronounced decrease in angiogenic factors or intratumoral pressure. We further observed that most differences between patients with non-pCR and pCR occurred early in therapy as well as prior to it, while tumour characteristics aligned more and more between patients over the course of therapy. This is expected as all patients responded to therapy, even those that did not achieve pCR.

This in-depth comparison between both response groups was only feasible through a (delta) radiomics approach, which is why we would like to point out its relevance for future studies. However, measurement conditions typically vary, resulting in differing DOR and impacting the comparability of extracted features. We encountered for this by utilising a compensated form of the vessel coverage.^36^ This compensation was not performed for the remaining features as it would require a detailed investigation of the impact of the DOR on every individual feature and adapted statistical modelling, which exceeded the scope of this study. Furthermore, as the DOR did not differ between the response groups (p = 0.54), an inherent bias is not to be expected in our case. Nevertheless, such investigations will be necessary in the future to enhance the stability of ULM-derived features.

Logistic regression based on ULM-derived features allowed to predict therapy response with 75% accuracy (AUC: 0.73) already before therapy and with 85% accuracy (AUC: 0.85) when combining the first two time points. However, from a clinical perspective, embedding several ULM measurements at defined time points as routine examination is challenging. Indicators of therapy response determined before therapy, in turn, would only require a single measurement and could be easily integrated in clinical decision making when determining the optimal therapy for each patient. Patients with disadvantageous vascular predisposition could then receive an alternative treatment regimen to increase their chances of achieving pCR, for example involving a higher dose of chemotherapeutics or the addition of agents inducing vascular normalisation.^51^^,^^52^ Additionally, de-escalation strategies could be developed to spare patients with a predicted pCR from unnecessary treatments. Here, the classification model design should focus on reaching high specificity, as patients already achieving pCR would be exposed to the higher risk of a more aggressive treatment otherwise, while misclassified patients with non-pCR would receive the current standard of care in the worst case.

We further showed that ULM outperformed conventional contrast-free ultrasound, standard analyses of contrast-enhanced ultrasound in the form of MIPs as well as histological CD31 staining in terms of predictive accuracy. Tumour size, which is regularly checked during NAC, did not allow any deduction on therapy response. Radiomic features extracted from B-mode ultrasound images, in turn, allowed to correctly classify 75% of patients based on longitudinal changes (AUC: 0.76) and the addition of contrast agent further improved response prediction (AUC: 0.79). Notably, histological CD31 masks, which highlight vessel walls, reached the highest predictive performance before NAC (accuracy: 75%, AUC: 0.83) but showed lower performance than longitudinal ULM.

Although our study provides strong evidence for distinct vascular differences between patients with non-pCR and pCR, these findings need to be considered with caution due to the limited number of included patients and the comparatively high number of extracted features. Nevertheless, the patient population and pCR rates were representative, although patients with HER2-positive and patients with invasive lobular carcinoma were slightly underrepresented, while patients with TNBC were slightly overrepresented.^38^ Please note that information on patients' race and ethnicity was not routinely collected, which should be considered when assessing the generalisability of our findings. We further ensured to maximise the comparability between patients by standardising MB injection, CEUS acquisition, and ULM post-processing. Moreover, we employed a conservative classification approach involving LOOCV, nested feature selection, and simple logistic regression, designed to reduce the risk of overfitting. A further limitation of the study is the differing NAC schemes between patients, which involve weekly or biweekly administration, resulting in varying time points (over 3 or 6 weeks). An alternative would have been to monitor patients over the same time period, however resulting in a different number of NAC cycles. Potential confounding arising from treatment regimen differences and clinical factors such as breast cancer subtype should be addressed in larger cohorts using multivariable modelling. Such larger-scale studies would also allow to improve generalisability of the results regarding patient demographics and characteristics.

Finally, we would like to emphasise that this study is exploratory and hypothesis-generating in nature, which is why identified features should be interpreted as candidate markers that require validation in larger patient studies. In this context, we would like to propose the early change in compensated vessel coverage as highly promising biomarker for pCR, which showed particularly high effect size (g = 2.21), accuracy (84%), and AUC (0.95), and the highest feature selection stability among all features (p_f_ = 100%). At the same time, the change in compensated vessel coverage was even more pronounced among patients with TNBC. The analysis of subgroup-specific differences within larger follow-up studies might therefore boost the predictive performance even further.

In conclusion, the extensive characterisation of tumour microvasculature using ULM enabled us to predict pCR already before therapy. Such an early diagnosis would allow for tailoring NAC of individual patients toward escalation or de-escalation and could considerably improve therapeutic outcomes as well as omit unnecessary toxicity. As a considerable proportion of patients currently do not achieve pCR, this would address an urgent clinical need, affecting many patients worldwide. At the same time, ULM proved to be practical in clinical routine, ensuring a total examination time below 10 min while using standard clinical ultrasound systems. Besides this specific application, ULM could also be employed to assess patients’ responses to other treatments, such as neoadjuvant radiotherapy, or to tailor breast surgery following NAC with the potential to fully omit this treatment step.

## Contributors

F.K., E.S., G.S., and P.K. conceptualised the study; M.K. was responsible for patient recruitment; M.K. and B.S.W. examined the patients and performed the ultrasound measurements, assisted by C.P. and Z.A.M.; E.S. supervised the clinical study and was responsible for study legalisation and documentation; A.R., M.K., and F.K. performed tumour segmentation; T.L. performed ULM processing; S.D. and G.S. supervised ULM processing; C.P. processed the final ULM data, performed feature extraction and data analysis, including classification; S.v.S. and P.B. were responsible for the histological staining; S.R.T. contributed to the statistical analysis plan; F.K. supervised the study as a whole, in particular the analysis of ULM data and the interpretation of the results; M.K. and E.S. contributed to setting the results into clinical context; C.P. prepared and drafted the manuscript and figures; all authors read and approved the final manuscript; C.P. and F.K. have direct access to the presented data and verified these.

## Data sharing statement

Upon reasonable request, data might be shared by the corresponding author in compliance with legal regulations and the protection of personal rights.

## Declaration of interests

F.K. and G.S. collaborate with Fujifilm Visualsonics on super-resolution ultrasound and F.K. additionally is advisor of this company and of BRACCO. F.K. and A.R. are co-owners of SonoMAC GmbH. F.K. is the council of the German Society for Biomedical Engineering (DGBMT) and was the president of the European Society for Molecular Imaging (ESMI) in 2023.
