# Supplementary material for: Ultrasound localisation microscopy predicts neoadjuvant chemotherapy response in patients with breast cancer
Source: eBioMedicine. 2026 Aug 24;131:106442. doi: 10.1016/j.ebiom.2026.106442 (PMC13524708; doi:10.1016/j.ebiom.2026.106442)
Supplement: Supplementary file 1 [file mmc1.pdf]

Ultrasound localisation microscopy predicts neoadjuvant chemotherapy response in patients with breast cancer

# – Supplementary material –

Supplementary methods 3

Analysis of biopsy tissue 3

Supplementary figures 4

Supplementary Fig. 1: Workflow of the histological CD31 analysis. 4

Supplementary Fig. 2: Workflow of the correction for multiple testing. 5

Supplementary Fig. 3: Workflow of classification. 6

Supplementary Fig. 4: Workflow of the sensitivity analysis of feature selection. 7

Supplementary Fig. 5: Flow chart of patient recruitment. 8

Supplementary Fig. 6: Occurrence maps of patient 16. 9

Supplementary Fig. 7: Example of a Euclidean distance map. 10

Supplementary Fig. 8: Change in compensated vessel coverage among patients with triple-negative and hormone receptor-positive breast cancer. 11

Supplementary tables 12

Supplementary Table 1: Design of the clinical study. 12

Supplementary Table 2: Overview of features extracted from ULM. 13

Supplementary Table 3: Overview of features extracted from comparative methods. 14

Supplementary Table 4: Overview of features extracted from individual measurements as well as changes between and combinations of several measurements. 15

Supplementary Table 5: Overview of grid search for the classification design. 16

Supplementary Table 6: Clinical information listed for all patients individually. 17

Supplementary Table 7: Dice similarity coefficients of the three annotators. 18

Supplementary Table 8: Summary statistics of ULM-based features for the three individual measurements.. 19

Supplementary Table 9: Summary statistics of ULM-based features for changes between measurements. 23

Supplementary Table 10: Summary statistics of ULM-based radiomic features for the three measurements combined. 37

Supplementary Table 11: Sensitivity analysis of feature selection based on ULM. 39

Supplementary Table 12: Comparison of feature selection probability among ULM and comparative methods. 41

Supplementary Table 13: Number of features with large effect sizes or significant nominal p-values among ULM or comparative methods. 42

Supplementary Table 14: Summary statistics of features extracted from B-mode size measurements for the three individual measurements. 43

Supplementary Table 15: Summary statistics of features extracted from B-mode size measurements for changes between measurements. 44

Supplementary Table 16: Summary statistics of features extracted from B-mode size measurements for the three measurements combined. 45

Supplementary Table 17: Summary statistics of features extracted from B-mode ultrasound images for the three individual measurements. 46

Supplementary Table 18: Summary statistics of features extracted from B-mode ultrasound images for changes between measurements. 47

Supplementary Table 19: Summary statistics of features extracted from B-mode ultrasound images for the three measurements combined. 50

Supplementary Table 20: Summary statistics of features extracted from contrast-mode MIPs for the three individual measurements. 51

Supplementary Table 21: Summary statistics of features extracted from contrast-mode MIPs for changes between measurements. 53

Supplementary Table 22: Summary statistics of features extracted from contrast-mode MIPs for the three measurements combined. 59

Supplementary Table 23: Summary statistics of features extracted from histological CD31 masks. 60

Supplementary Table 24: Features considered significant after Benjamini-Hochberg correction. 61

Supplementary Table 25: Sensitivity analysis of feature selection based on B-mode size measurements. 62

Supplementary Table 26: Sensitivity analysis of feature selection based on B-mode ultrasound images. 63

Supplementary Table 27: Sensitivity analysis of feature selection based on contrast-mode MIPs. 64

Supplementary Table 28: Sensitivity analysis of feature selection based on histological CD31 masks. 65

References 66

Study protocol 67

Supplementary methods

Analysis of biopsy tissue

The extracted biopsy tissue was fixed with formalin and embedded in paraffin. It was then stained with hematoxylin & eosin to identify cancerous regions and stained for CD31 to analyse microvessels.

HE staining: Per patient, a slice of 4–5 µm was cut using a Microtome (Rotationsmikrotom Hyrax M, Microm Laborgeräte GmbH / Carl Zeiss Jena GmbH, Jena, Germany) and mounted on a glass microscopy slide. The slides were stained following a standard protocol in a staining and film coverslipping integrated system (Tissue-Tek Prisma Plus & Tissue-Tek Film, Sakura Finetek Germany GmbH, Umkirch, Germany).

CD31 staining: Per patient, a slice of 2–4 µm was cut using a Microtome (Rotationsmikrotom Hyrax M, Microm Laborgeräte GmbH / Carl Zeiss Jena GbmH, Jena, Germany) and mounted on a glass microscopy slide. Antigen retrieval was carried out using a pre-treatment module (PT Link, Agilent Technologies Germany GmbH, Waldbronn, Germany) with heat-induced epitope retrieval (HIER) and by pre-treating the tissue with EnVision FLEX Target Retrieval Solution, High pH (Code 8004). The slides were then incubated with a monoclonal mouse anti-human CD31 antibody (endothelial cell clone JC70A ready-to-use (link), Code IR610, Dako Denmark A/S, Glostrup, Denmark), a linker (Code SM804), horseradish peroxidase (Code SM802), and DAB (Code SM803) on an Autostainer Link 48 (Agilent Technologies Germany GmbH, Waldbronn, Germany). Counterstaining was performed using hematoxylin (Code SM806). Finally, the slides were covered in an automated coverslipping system (Tissue-Tek Prisma Plus & Tissue-Tek Film, Sakura Finetek Germany GmbH, Umkirch, Germany).

Image acquisition: The slides were scanned with the 40x objective of an Aperio AT2 whole slide scanner (Leica Biosystems, Wetzlar, Germany). Images were taken using the Aperio ImageScope 12.4.6 software (Leica Biosystems, Wetzlar, Germany).

Generation of CD31 masks: Binary CD31 masks were computed within the cancerous regions of the CD31 staining using a pixel width of 0.63 µm. This was done by thresholding the RGB image, including only pixels where all three colour channels show a value below 100, and by subsequently excluding objects smaller than 100 pixels to reduce background.

Supplementary figures

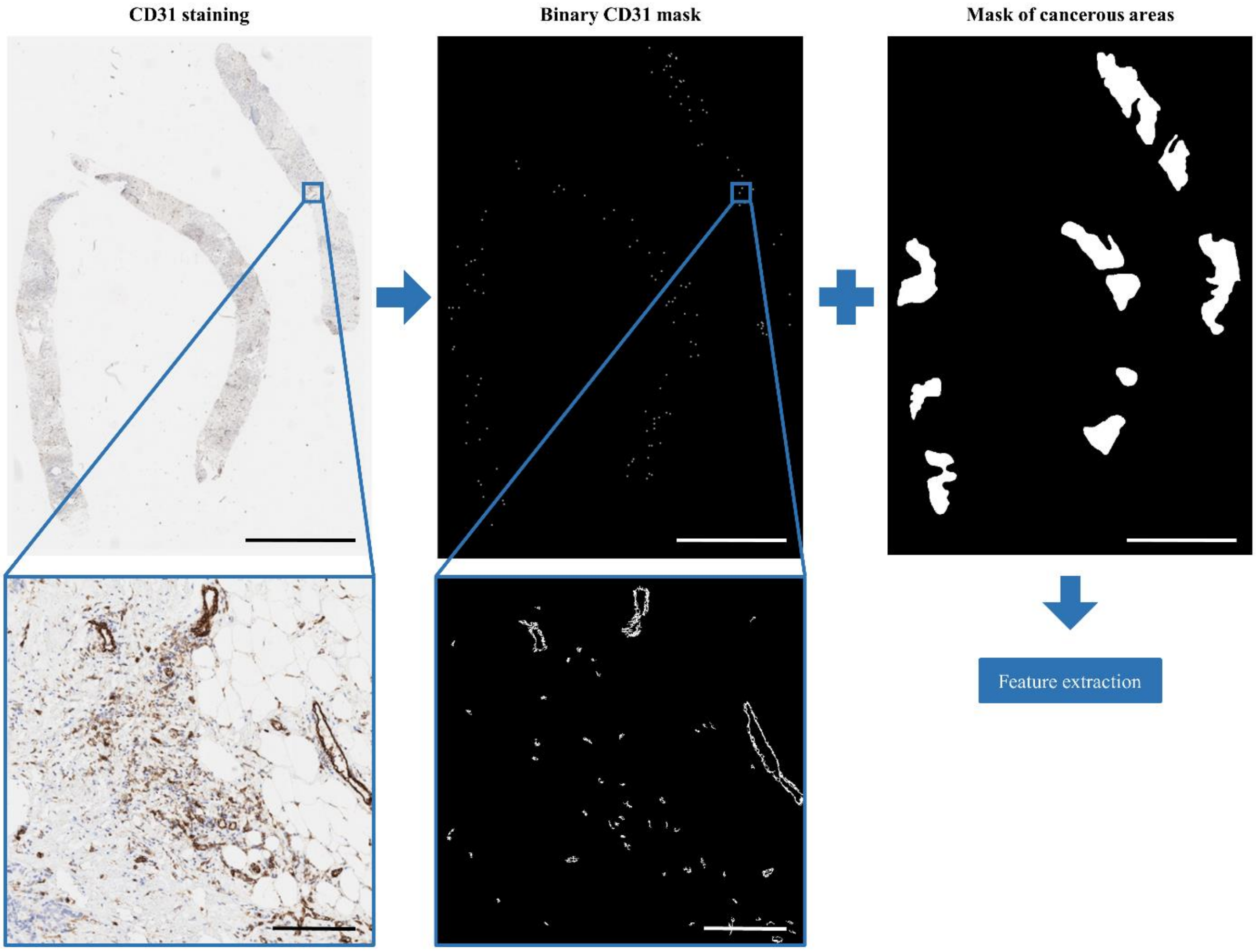

Supplementary Fig. 1: Workflow of the histological CD31 analysis. Scale bars: 5 mm (overview), 200 µm (zoom).

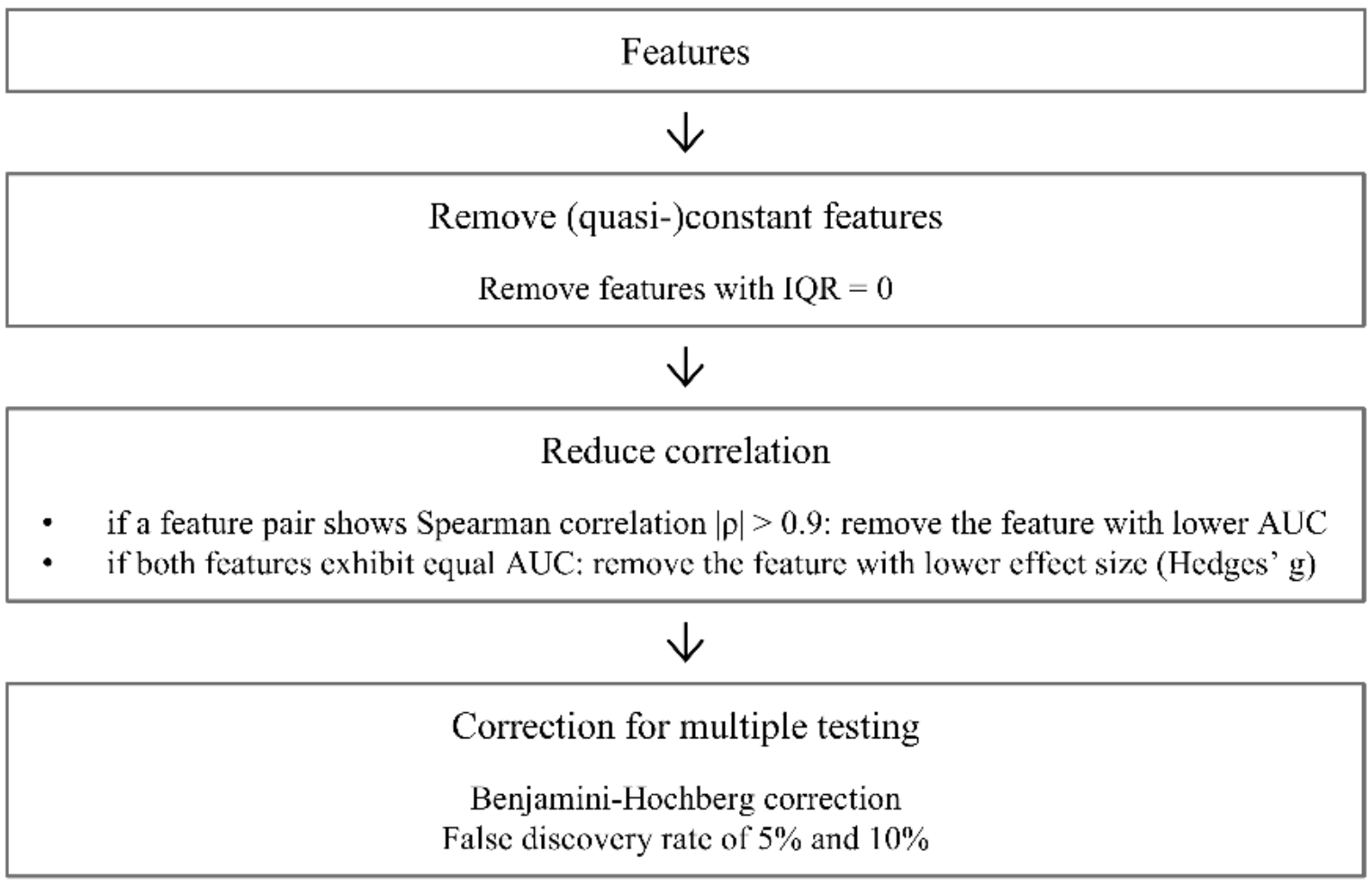

Supplementary Fig. 2: Workflow of the correction for multiple testing. Non-informative (quasi-)constant features with an interquartile range (IQR) of zero were removed. Feature correlation was determined using Spearman's ρ and, for each feature pair with |ρ| > 0.9, the feature with the lower predictive performance (i.e., the lower area under the receiver operating curve, AUC) was removed. If both features exhibited equal AUC, the feature with the lower effect size was removed. The remaining features were subjected to Benjamini-Hochberg correction to control the false discovery rate at 5% or 10%.

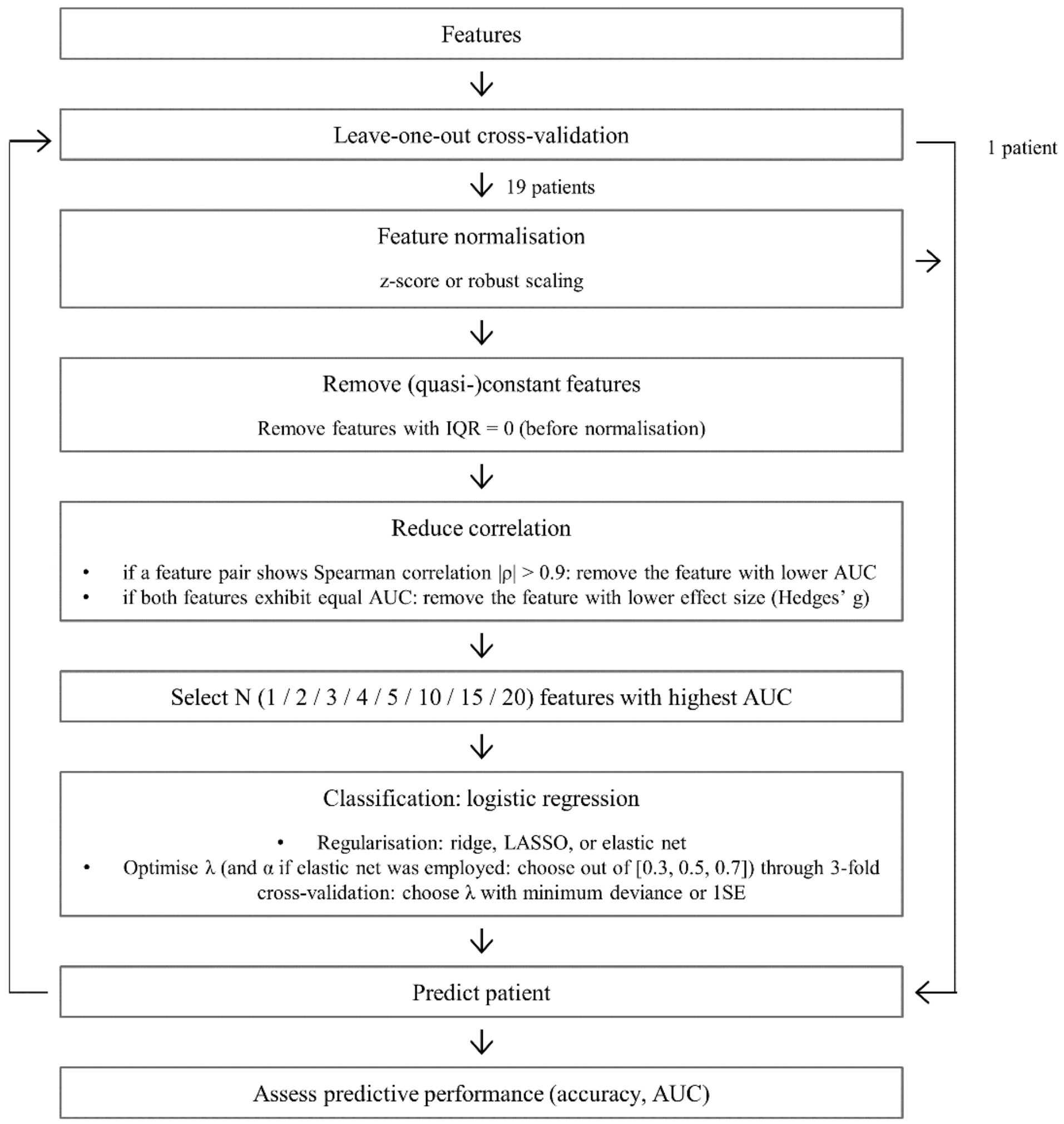

Supplementary Fig. 3: Workflow of classification. Feature selection and classification were embedded within a leave-one out cross-validation framework. For feature selection, features were first normalised using z-score or robust scaling. Non-informative (quasi-)constant features with an interquartile range (IQR) of zero before normalisation were removed. Feature correlation was determined using Spearman's ρ and, for each feature pair with |ρ| > 0.9, the feature with the lower predictive performance (i.e., the lower area under the receiver operating curve, AUC) was removed. If both features exhibited equal AUC, the feature with the lower effect size was removed. Finally, the N features with the highest AUC were selected for classification. Logistic regression was performed using ridge, LASSO, or elastic net regularisation. During model training, the regularisation parameter λ was optimised using three-fold cross-validation, the optimal value being selected either as the one minimising the cross-validated deviance or according to the one-standard-error (1-SE) rule to favour simpler models. For elastic net, the hyperparameter α was additionally optimised by selecting the best-performing value among 0.3, 0.5, and 0.7. Predictive performance was evaluated via the predictive accuracy and the AUC.

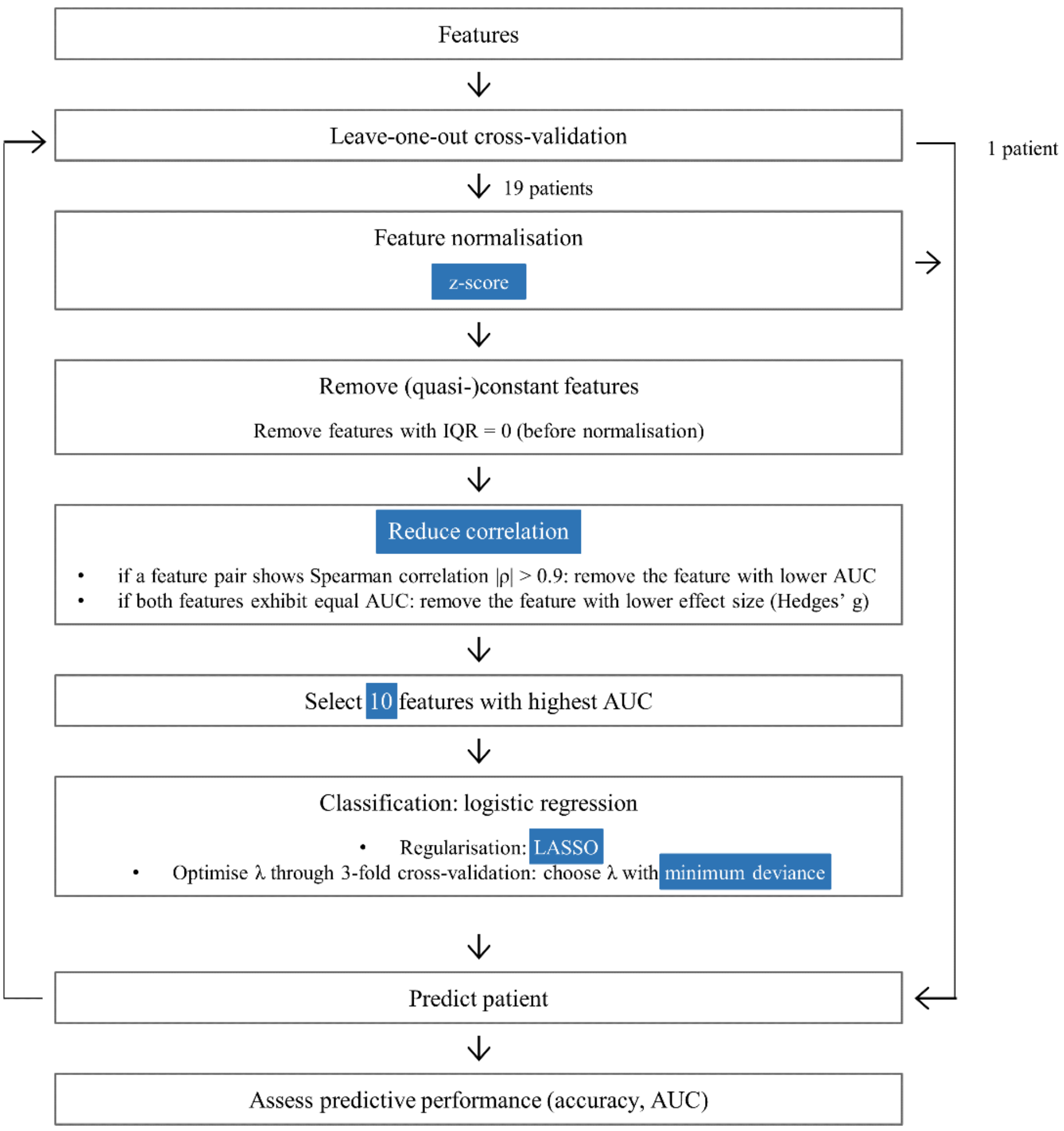

Supplementary Fig. 4: Workflow of the sensitivity analysis of feature selection. Feature selection and classification were embedded within a leave-one out cross-validation framework. For feature selection, features were first normalised using z-score. Non-informative (quasi-)constant features with an interquartile range (IQR) of zero before normalisation were removed. Feature correlation was determined using Spearman's ρ and, for each feature pair with |ρ| > 0.9, the feature with the lower predictive performance (i.e., the lower area under the receiver operating curve, AUC) was removed. If both features exhibited equal AUC, the feature with the lower effect size was removed. Finally, the 10 features with the highest AUC were selected for classification. Logistic regression was performed using LASSO regularisation, which involved further feature selection. During model training, the regularisation parameter λ was optimised using three-fold cross-validation, the optimal value being selected as the one minimising the cross-validated deviance. The features selected by LASSO were saved for each of the 20 LOOCV loops to determine the feature selection frequency.

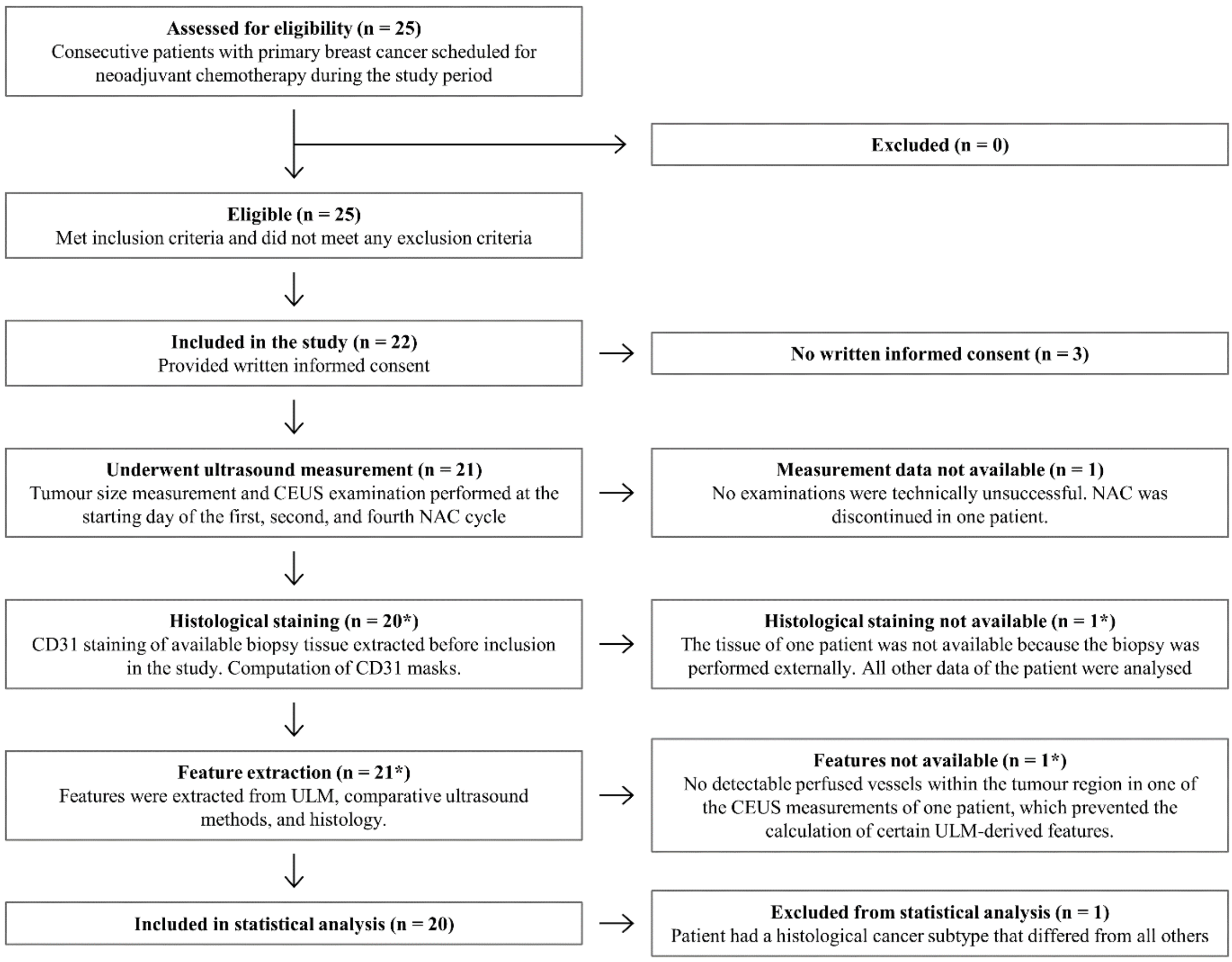

*Patients with missing histological staining or individual missing features were not excluded from statistical analysis.

Supplementary Fig. 5: Flow chart of patient recruitment.

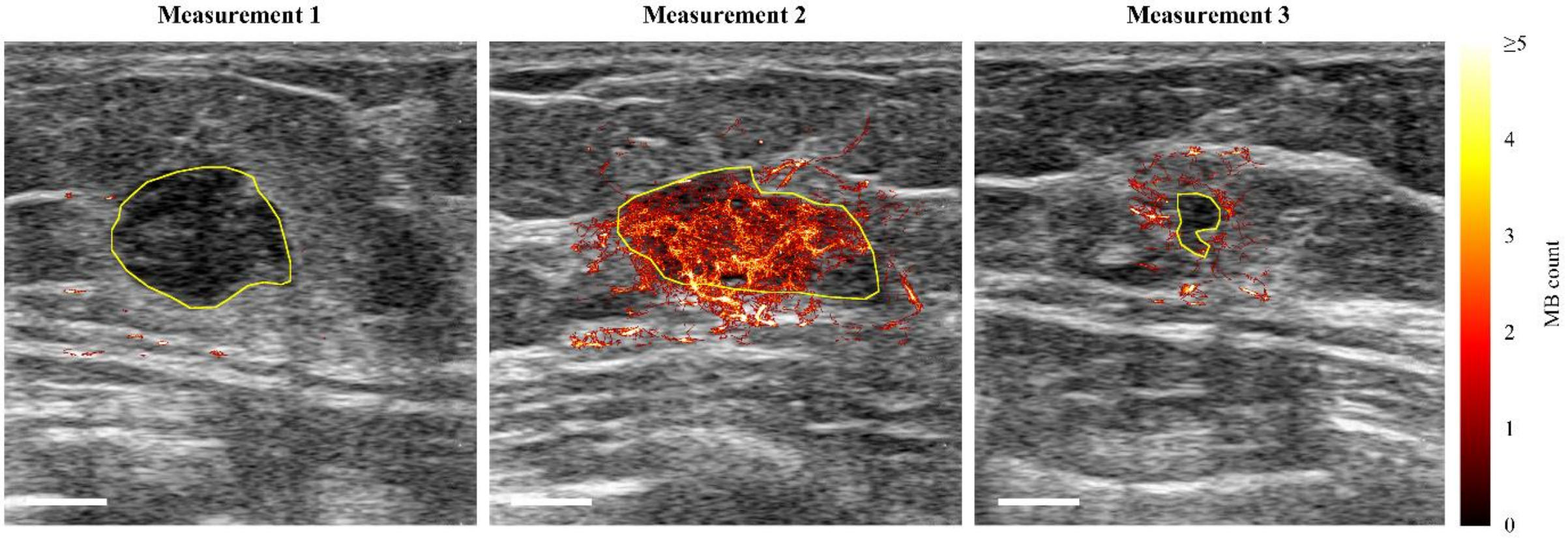

Supplementary Fig. 6: Occurrence maps of patient 16. At baseline, no vascularisation was detectable within the primary tumour. In response to the first chemotherapy cycle, vascularisation drastically increased, followed by a decrease in both tumour size and vascularisation degree after the third chemotherapy cycle. Scale bars: 5 mm.

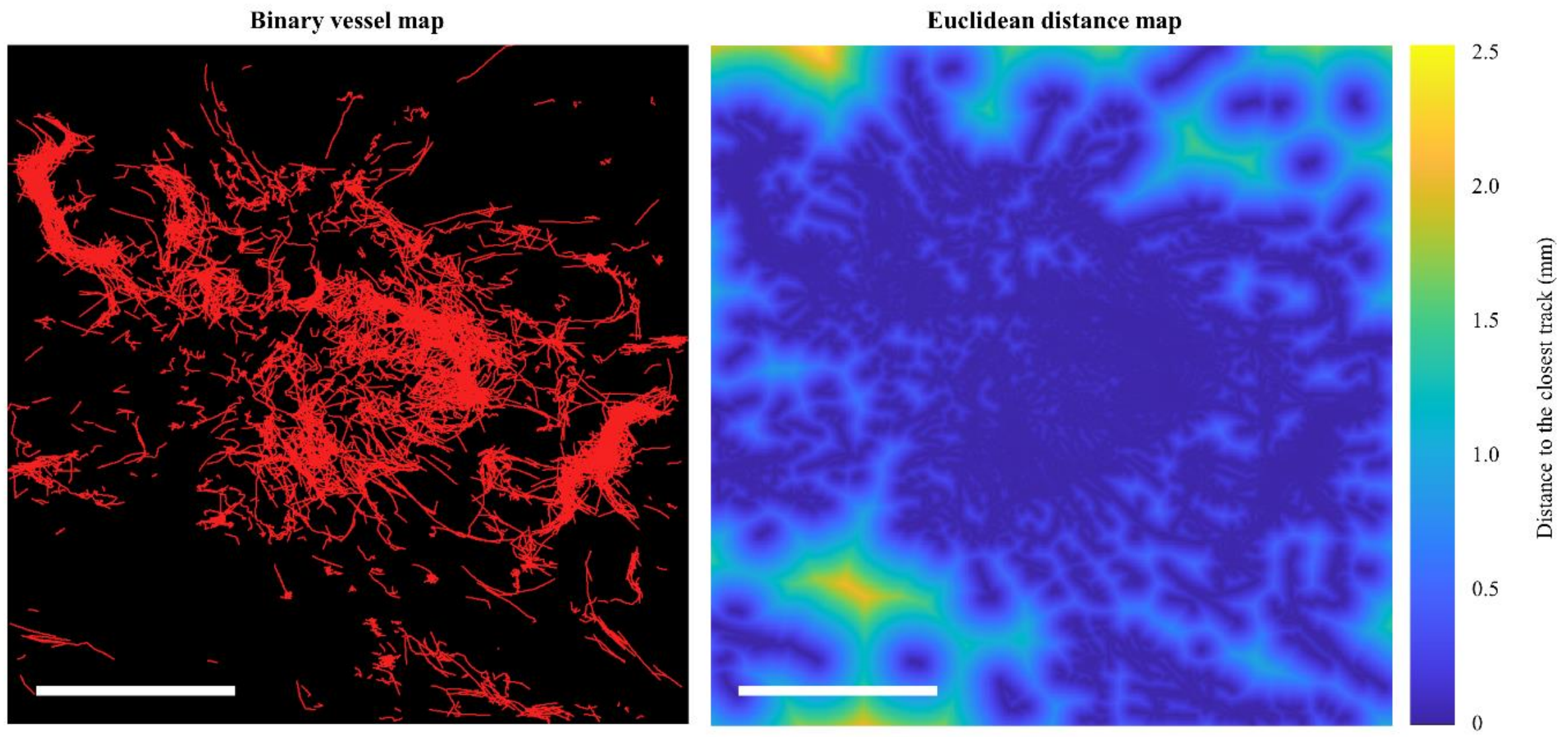

Supplementary Fig. 7: Example of a Euclidean distance map. The distance map represents the distance of each pixel to the closest track in mm and is calculated based on the respective binary map. Scale bars: 5 mm.

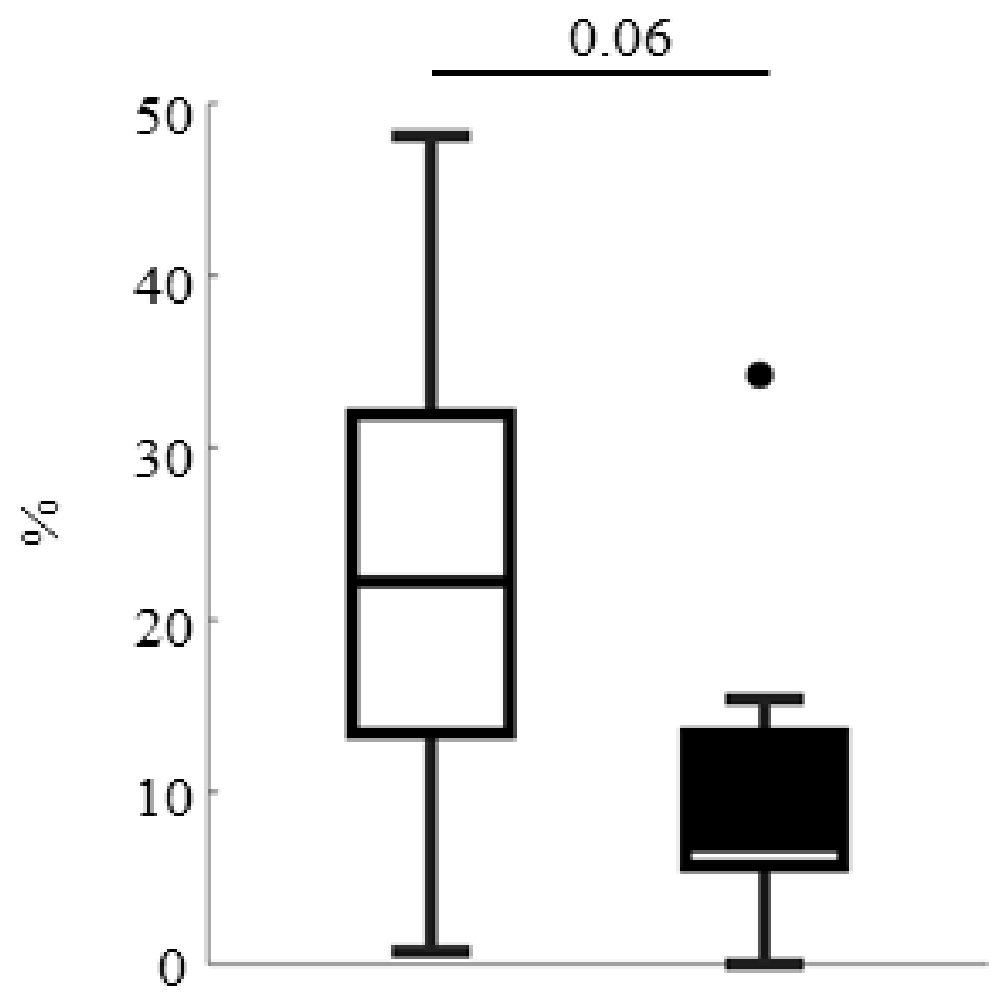

Supplementary Fig. 8: Change in compensated vessel coverage among patients with triple-negative and hormone receptor-positive breast cancer. The boxplot represents the absolute change in compensated vessel coverage between measurement 1 and 2 (n_{TNBC}=8, n_{HR+}=9). The p-value was determined via a two-sided Wilcoxon rank sum test.

Supplementary tables

Supplementary Table 1: Design of the clinical study.

**Study design**	Monocentric open interventional study
**Inclusion criteria**	Female patients<br>- with histologically confirmed primary breast cancer, including all intrinsic subtypes<br>- which receive a neoadjuvant chemotherapy treatment<br>- which gave written consent<br>- which are legally competent and mentally able to follow the instructions of the study staff
**Exclusion criteria**	- Minority (younger than 18 years)<br>- Hypersensitivity/allergy against the ultrasound contrast agent (SonoVue®) or its compounds sulphur hexafluoride, Macrogol 4000, distearoylphosphati-dylcholine, dipalmitoylphosphatidylglycerol-sodium, palmitic acid<br>- Right-left shunt<br>- Signs of cardiovascular instability<br>- Acute endocarditis<br>- Artificial heart valves<br>- Acute systemic inflammations and/or sepsis<br>- Overactive coagulation status and/or recent thromboembolism<br>- End-stage kidney or liver disease<br>- Severe pulmonary hypertension (pulmonary arterial pressure > 90 mmHg)<br>- Uncontrolled systemic hypertension<br>- Acute respiratory distress syndrome<br>- Pregnancy<br>- Alcohol or drug abuse<br>- Commitment to any resident institution by order of any court or authority<br>- Persons who are accommodated in an institution on official or judicial instruction<br>- Persons who are in a dependent or employment relationship with the sponsor or investigator<br>- Expected non-compliance
**Primary outcome measure**	- Relative blood volume in % (i.e., its estimation based on ULM, meaning the vessel coverage)<br>- Blood flow in mm/s<br>- Change of relative blood volume in %<br>- Change of blood flow in mm/s
**Secondary outcome measure**	- Change of tumour size determined based on ultrasound B-mode imaging

Supplementary Table 2: Overview of features extracted from ULM.

Category	Parameters	Subparameters	n	Definition	Source data
morphology	vessel coverage	• uncompensated (listed for the sake of completeness; not considered a part of the ULM feature set)<br>• compensated	1	• uncompensated:<br>$VC_{\text{uncomp}} = \frac{n_{\text{signal}}}{N_{\text{pixels}}}$<br>$n_{\text{signal}}$: number of pixels with signal<br>N_{pixels}: total number of pixels<br>• compensated:<br>$VC_{\text{comp}} = \frac{n_{\text{signal}}}{N_{\text{pixels}}(1-e)^{-\hat{\Lambda}}}$<br>$\hat{\Lambda} = W_0\left(-\frac{C}{n_{\text{signal}}} e^{-\frac{C}{n_{\text{signal}}}}\right) + \frac{C}{n_{\text{signal}}}$<br>$C$: sum of the MB counts of all pixels<br>$W_0(x)$: main branch of Lambert's W-function<br>• compensated (case $VC_{\text{comp}} > 1$):<br>$VC_{\text{comp}} = 1$<br>$\hat{\Lambda} = \frac{C}{N_{\text{pixels}}}$	occurrence map
	distance to the closest track	statistical features*	7	intensity-based statistical features of a Euclidean distance map, which represents pixel-wise the distance to the closest track in mm	binary track map
	flow direction	variance	1	variance of the flow direction map in rad	direction map
	track length	statistical features*	7	statistical features of the track lengths, which were calculated track-wise as the sum of individual track segment lengths:<br>$l_i = \sum_{n=1}^{N_i-1} \lvert \boldsymbol{x}_{n+1,i} - \boldsymbol{x}_{n,i} \rvert$<br>$l_i$: length of track i<br>N_i: number of MB positions within track i<br>$\boldsymbol{x}_{n,i}$: position vector of the nth MB position within track i in m	raw track data**
	tortuosity	statistical features* of<br>• distance metric (DM)<br>• sum of angles metric (SOAM)	14	statistical features of the tortuosity, which was calculated for each track i using different metrics:<br>• distance metric<br>$DM_i = \frac{l_i}{\lvert \boldsymbol{x}_{N_i} - \boldsymbol{x}_1 \rvert}$<br>$l_i$: length of track i<br>N_i: number of MB positions within track i<br>$\boldsymbol{x}_{n,i}$: position vector of the nth MB position within track i in m<br>• sum of angles metric<br>$SOAM_i = \frac{\sum_{n=1}^{N_i-1} \varphi_{n,n+1}}{l_i}$<br>$\varphi_{n,n+1}$: angle between the nth and $n+1$th MB position	raw track data**
function	velocity	statistical features*	7	intensity-based statistical features of the velocity map in mm/s (in case tracks overlapped in a pixel, the mean velocity was depicted in the map)	velocity map
	track velocity range	statistical features*	7	statistical features of the velocity range, which was calculated track-wise as:<br>$VR_i = \max_n \boldsymbol{v}_{n,i} - \min_n \boldsymbol{v}_{n,i}$<br>$\boldsymbol{v}_{n,i}$: velocity vector at the nth MB position within track i in m/s	raw track data**
texture	fractal analysis	Hausdorff fractal dimension, R^2 (goodness of fit)	2	calculated using the box-counting method[2], R^2 being the squared correlation coefficient of the box width and box count	binary track map

* mean, variance, skewness, kurtosis, median, interquartile range, range; defined according to Zwanenburg et al.[1]
** set of all tracks, i.e., MB positions and velocities of each single track

Supplementary Table 3: Overview of features extracted from comparative methods.

Data	Category	Name	n	Definition
Tumour size measurement		tumour volume	1	$V = {}^{1}/_{6}\,\pi \cdot D_1 \cdot D_2 \cdot D_3$<br>where D_1 represents the largest tumour diameter and D_2 and D_3 the two diameters perpendicular to it
B-mode ultrasound image	morphological	histogram features*	7	histogram analysis of the grey levels
	texture	fractal analysis	2	see Supplementary Table 2
	**total**		**9**	
Contrast-mode MIP	morphological	vessel coverage**	1	see Supplementary Table 2, uncompensated vessel coverage
		distance to the closest vessel**	7	see Supplementary Table 2
		histogram features*	7	histogram analysis of the grey levels
	texture	fractal analysis**	2	see Supplementary Table 2
	**total**		**17**	
Histological CD31 mask	morphological	vessel coverage	1	see Supplementary Table 2, uncompensated vessel coverage
		distance to the closest vessel	7	see Supplementary Table 2
	texture	fractal analysis	2	see Supplementary Table 2
	**total**		**10**	

* mean, variance, skewness, kurtosis, median, interquartile range, range; defined according to Zwanenburg et al.[1]
** calculated after binarising the contrast-mode MIP using a grey-level threshold of 85

Supplementary Table 4: Overview of features extracted from individual measurements as well as changes between and combinations of several measurements.

Measurement	Sub-features	# of features
individual measurements (1 | 2 | 3)	$F^{(1)}$ | $F^{(2)}$ | $F^{(3)}$	*N*
changes between two measurements	• relative change (ΔF)<br>• absolute change ($\|\Delta F\|$)<br>• minimum (F_{min})<br>• maximum (F_{max})	*4.N*
combination of two measurements	• features of individual measurement 1 ($F^{(1)}$)<br>• features of individual measurement 2 ($F^{(2)}$)<br>• changes between both measurements ($\Delta F^{(1,2)}$, $\|\Delta F^{(1,2)}\|$, $F_{\mathrm{min}}^{(1,2)}$, $F_{\mathrm{max}}^{(1,2)}$)	*6.N*
combination of three measurements	• features of individual measurement 1 ($F^{(1)}$)<br>• features of individual measurement 2 ($F^{(2)}$)<br>• features of individual measurement 3 ($F^{(3)}$)<br>• changes between measurement 1 and 2 ($\Delta F^{(1,2)}$, $\|\Delta F^{(1,2)}\|$, $F_{\mathrm{min}}^{(1,2)}$, $F_{\mathrm{max}}^{(1,2)}$)<br>• changes between measurement 2 and 3 ($\Delta F^{(2,3)}$, $\|\Delta F^{(2,3)}\|$, $\Delta F_{\mathrm{min}}^{(2,3)}$, $\Delta F_{\mathrm{max}}^{(2,3)}$)<br>• changes between measurement 1 and 3 ($\Delta F^{(1,3)}$, $\|\Delta F^{(1,3)}\|$, $\Delta F_{\mathrm{min}}^{(1,3)}$, $\Delta F_{\mathrm{max}}^{(1,3)}$)<br>• minimum of the three measurements ($F_{\mathrm{min}}^{(1,2,3)}$)<br>• maximum of the three measurements ($F_{\mathrm{max}}^{(1,2,3)}$)	*17.N*

Supplementary Table 5: Overview of grid search for the classification design.

Design choice	Option
feature normalisation	• z-score<br>• robust scaling
regularisation – method	• ridge<br>• LASSO<br>• elastic net
regularisation – choice of optimal λ	• minimum deviance<br>• one-standard-error (1-SE) rule
reduction of feature correlation	• yes<br>• no (only if LASSO or elastic net regularisation)
number of selected features	• 1<br>• 2<br>• 3<br>• 4<br>• 5<br>• 10 (only if LASSO or elastic net regularisation)<br>• 15 (only if LASSO or elastic net regularisation)<br>• 20 (only if LASSO or elastic net regularisation)

Supplementary Table 6: Clinical information listed for all patients individually.

Patient	Age (y)	Tumour type	Histological subtype	ER (%)	PR (%)	HER2	Ki67 (%)	Grade	NAC*	NAC cycle*	Additional therapy	Primary tumour remaining‡	Lymph node remaining‡
1	49	TNBC	no special type	0	0	0	90	G3	CP	weekly	/	ypT1a	ypN0
2	73	TNBC	no special type	5	0	2+/FISH-	41	G2	CP	weekly	pembrolizumab†	ypT1c	ypN0
3	71	HER2+	no special type	100	25	3+	35	G2	P	weekly	trastuzumab, pertuzumab†	ypT1mi	ypN0
4	68	HR+	no special type	100	2	0	50	G3	EC	biweekly	/	ypT3	ypN1a
5	62	HR+	no special type	100	99	1+	68	G3	EC	biweekly	/	ypT0	ypN0
6	48	TNBC	no special type	0	< 1	0	48	G2	CP	weekly	pembrolizumab†	ypT0	ypN0
7	34	TNBC	no special type	2	2	1+	80	G3	CP	weekly	/	ypT0	ypN0
8	61	HER2+	no special type	0	0	3+	54	G3	EC	biweekly	trastuzumab, pertuzumab†	ypT0	ypN0
9	31	TNBC	no special type	0	< 1	2+/FISH-	77	G3	CP	weekly	pembrolizumab†	ypT0	ypN0
10	60	HR+	no special type	90	10	0	50	G2	EC	biweekly	/	ypT2	ypN0(i+)
11	51	TNBC	no special type	0	0	0	40	G2	CP	weekly	pembrolizumab†	ypT0	ypN0
12	50	HR+	no special type	100	100	0	45	G2	EC	biweekly	/	ypT2	ypN1a
13	67	HR+	no special type	100	0	1+	45	G3	EC	biweekly	/	ypT1c	ypN0
14	35	TNBC	no special type	0	0	0	> 80	G3	CP	weekly	pembrolizumab†	ypT0	ypN0 (i+)
15	38	HR+	no special type	90	90	2+/FISH-	40	G3	EC	biweekly	/	ypT2	ypN1a
16	45	TNBC	no special type	0	0	2+/FISH-	90	G3	CP	weekly	pembrolizumab†	ypT0	ypN0
17	55	HR+	no special type	100	60	2+/FISH -	25	G3	EC	biweekly	/	ypT1b	ypN0
18	54	HR+	no special type	100	0	1+	30	G3	EC	biweekly	/	ypT1b	ypN0
19	37	HR+	no special type	100	60	1+	70	G3	EC	biweekly	/	ypT0	ypN1a
20	55	TNBC	no special type	0	0	0+	35	G3	CP	weekly	/	ypT1a	ypN0

Patients with pathological complete response are marked in red.
*referring to the first three NAC cycles
†every 3 weeks
‡according to the TNM classification
CP: carboplatin + paclitaxel, EC: epirubicin + cyclophosphamide, ER: oestrogen receptor, FISH-: negative fluorescence in situ hybridisation test, HER2: human epidermal growth factor receptor 2, HER2+: HER2 positive, HR+: hormone receptor positive, NAC: neoadjuvant chemotherapy, P: paclitaxel, PR: progesterone receptor, TNBC: triple-negative breast cancer, y: years

Supplementary Table 7: Dice similarity coefficients of the three annotators. Coefficients were calculated for the tumour ROIs of all patients and measurements. Values are reported as median values with interquartile ranges.

	**Annotator 1 vs. 2**	**Annotator 2 vs. 3**	**Annotator 1 vs. 3**	**Total**
**Dice similarity coefficient**	0.83 [0.73, 0.89]	0.85 [0.76, 0.89]	0.80 [0.65, 0.89]	0.83 [0.72, 0.89]

Supplementary Table 8: Summary statistics of ULM-based features for the three individual measurements. From left to right: feature name, p-value for a statistical significance between patients with pCR and non-pCR, mean ± standard deviation of patients with pCR, mean ± standard deviation of patients with non-pCR, effect size expressed by Hedges' g with bootstrapped 95% confidence interval, cross-validated predictive accuracy, area under the receiver operating characteristic curve (AUC).

	Measurement 1						Measurement 2						Measurement 3					
**Feature**	**p**	**pCR**	**non-pCR**	**Effect size**	**Acc (%)**	**AUC**	**p**	**pCR**	**non-pCR**	**Effect size**	**Acc (%)**	**AUC**	**p**	**pCR**	**non-pCR**	**Effect size**	**Acc (%)**	**AUC**
compensated vessel coverage (%)	0.0221	29.69 ± 16.98	12.75 ± 13.75	1.08 [-0.01, 2.48]	58	0.82	0.1325	25.17 ± 22.32	11.87 ± 12.92	0.74 [-0.46, 1.87]	55	0.71	0.6574	17.44 ± 17.65	11.22 ± 10.38	0.43 [-0.67, 1.67]	47	0.57
distances – mean (mm)	0.1770	0.36 ± 0.39	1.22 ± 1.05	-0.94 [-1.62, -0.27]	60	0.79	0.2976	0.73 ± 0.70	1.38 ± 1.49	-0.50 [-1.12, 0.30]	60	0.65	0.3348	0.91 ± 1.19	1.01 ± 0.83	-0.10 [-1.00, 1.79]	60	0.60
distances – variance (mm^2)	0.0449	0.26 ± 0.39	1.60 ± 2.35	-0.67 [-1.02, 0.17]	68	0.79	0.3749	0.62 ± 0.66	2.19 ± 2.81	-0.67 [-1.53, -0.12]	60	0.63	0.5999	1.40 ± 3.04	1.41 ± 1.94	-0.01 [-0.81, 1.43]	58	0.58
distances – skewness	0.1956	1.97 ± 0.78	1.42 ± 1.06	0.53 [-0.54, 1.69]	47	0.69	0.2318	2.17 ± 1.44	1.31 ± 0.79	0.76 [-0.14, 1.84]	65	0.67	0.8404	1.39 ± 0.70	1.53 ± 0.82	-0.16 [-1.09, 0.84]	58	0.53
distances – kurtosis	0.2614	4.84 ± 4.18	3.16 ± 5.75	0.31 [-0.78, 1.30]	53	0.67	0.1535	7.25 ± 8.47	2.15 ± 3.43	0.82 [-0.30, 1.85]	60	0.70	0.7168	2.12 ± 2.64	3.09 ± 4.69	-0.23 [-1.04, 0.67]	58	0.56
distances – median (mm)	0.0972	0.22 ± 0.27	0.95 ± 0.97	-0.87 [-1.42, -0.23]	60	0.83	0.3749	0.57 ± 0.66	1.04 ± 1.41	-0.38 [-1.00, 0.55]	60	0.63	0.3347	0.69 ± 0.96	0.69 ± 0.63	0.00 [-0.97, 1.25]	60	0.60
distances – interquartile range (mm)	0.0683	0.48 ± 0.57	1.34 ± 1.15	-0.84 [-1.53, -0.05]	53	0.76	0.2976	0.78 ± 0.76	1.48 ± 1.39	-0.57 [-1.26, 0.38]	60	0.65	0.5281	1.17 ± 1.68	1.14 ± 1.02	0.02 [-0.93, 1.34]	58	0.59
distances – range (mm)	0.0221	2.07 ± 1.44	4.85 ± 3.40	-0.93 [-1.66, -0.15]	68	0.82	0.5628	3.46 ± 1.91	5.21 ± 3.95	-0.51 [-1.41, 0.33]	60	0.58	0.3950	3.48 ± 3.39	4.71 ± 3.40	-0.35 [-1.18, 0.66]	53	0.63
flow direction – variance (rad^2)	0.9018	3.42 ± 0.23	3.43 ± 0.35	-0.03 [-0.87, 0.95]	58	0.52	0.7285	3.35 ± 0.31	3.29 ± 0.53	0.13 [-0.84, 0.94]	60	0.55	0.8404	3.46 ± 0.35	3.68 ± 0.54	-0.44 [-1.24, 0.43]	58	0.53
track length – mean (mm)	0.1673	0.65 ± 0.11	0.52 ± 0.17	0.75 [-0.12, 1.45]	58	0.70	0.0587	0.63 ± 0.08	0.53 ± 0.11	0.97 [0.28, 1.82]	60	0.76	0.3511	0.59 ± 0.13	0.52 ± 0.16	0.43 [-0.55, 1.27]	58	0.64
track length – variance (mm^2)	0.3402	0.30 ± 0.11	0.22 ± 0.13	0.63 [-0.27, 1.54]	58	0.64	0.1770	0.29 ± 0.07	0.22 ± 0.12	0.62 [-0.28, 1.48]	60	0.69	0.6574	0.28 ± 0.13	0.25 ± 0.14	0.23 [-0.71, 1.24]	58	0.57
track length – skewness	0.7732	1.99 ± 0.31	1.96 ± 0.47	0.07 [-0.87, 0.94]	47	0.55	0.6574	2.19 ± 0.42	2.00 ± 0.48	0.39 [-0.58, 1.28]	58	0.57	0.7780	2.11 ± 0.46	2.32 ± 0.67	-0.34 [-1.22, 0.51]	58	0.55

Feature	Measurement 1						Measurement 2						Measurement 3					
	p	pCR	non-pCR	Effect size	Acc (%)	AUC	p	pCR	non-pCR	Effect size	Acc (%)	AUC	p	pCR	non-pCR	Effect size	Acc (%)	AUC
track length – kurtosis	0.5918	6.19 ± 2.12	5.78 ± 3.18	0.14 [-0.83, 1.19]	47	0.58	0.3511	7.43 ± 3.25	5.80 ± 3.36	0.47 [-0.48, 1.37]	42	0.64	0.7168	6.22 ± 2.63	8.33 ± 5.96	-0.41 [-1.09, 0.55]	53	0.56
track length – median (mm)	0.1422	0.50 ± 0.10	0.40 ± 0.14	0.76 [-0.23, 1.51]	58	0.71	0.0279	0.50 ± 0.07	0.40 ± 0.08	1.23 [0.29, 2.05]	75	0.80	0.3100	0.45 ± 0.11	0.37 ± 0.13	0.60 [-0.29, 1.51]	58	0.65
track length – interquartile range (mm)	0.2614	0.59 ± 0.07	0.48 ± 0.18	0.66 [-0.06, 1.26]	58	0.67	0.1325	0.57 ± 0.07	0.47 ± 0.18	0.66 [-0.12, 1.20]	60	0.71	0.4421	0.54 ± 0.13	0.48 ± 0.15	0.41 [-0.66, 1.27]	58	0.61
track length – range (mm)	0.1003	4.56 ± 1.43	3.28 ± 1.59	0.79 [-0.23, 1.77]	47	0.74	0.1770	3.92 ± 1.11	3.01 ± 1.55	0.63 [-0.17, 1.39]	55	0.69	0.9039	3.62 ± 1.55	3.67 ± 1.78	-0.03 [-1.00, 0.82]	58	0.52
tortuosity (DM) – mean	0.2991	2.23 ± 0.26	2.67 ± 0.84	-0.61 [-1.13, 0.20]	58	0.65	0.0409	2.25 ± 0.67	2.75 ± 1.30	-0.43 [-1.04, 1.14]	55	0.78	0.3511	2.57 ± 0.52	2.29 ± 0.26	0.69 [-0.35, 1.64]	63	0.64
tortuosity (DM) – variance	0.6504	13.48 ± 10.01	12.09 ± 7.56	0.16 [-0.89, 1.30]	47	0.57	0.5628	8.78 ± 8.05	11.50 ± 11.22	-0.26 [-1.03, 0.76]	60	0.58	0.1774	19.22 ± 18.38	8.31 ± 5.23	0.84 [-0.15, 1.85]	68	0.69
tortuosity (DM) – skewness	0.0221	12.23 ± 6.38	6.37 ± 2.92	1.25 [-0.09, 2.40]	63	0.82	0.7168	5.77 ± 2.35	7.09 ± 6.19	-0.25 [-1.16, 0.68]	58	0.56	0.5999	6.56 ± 4.20	5.63 ± 3.28	0.24 [-0.82, 1.24]	47	0.58
tortuosity (DM) – kurtosis	0.0283	272.71 ± 259.09	67.35 ± 57.71	1.22 [-0.20, 2.21]	63	0.81	0.7168	52.49 ± 51.70	110.89 ± 178.29	-0.40 [-1.11, 0.53]	58	0.56	0.7780	75.46 ± 87.93	58.90 ± 81.85	0.19 [-0.74, 1.11]	47	0.55
tortuosity (DM) – median	0.1956	1.34 ± 0.05	1.60 ± 0.44	-0.70 [-1.26, -0.29]	53	0.69	0.0279	1.33 ± 0.17	1.83 ± 1.54	-0.40 [-1.00, 0.55]	50	0.80	0.7168	1.45 ± 0.19	1.40 ± 0.18	0.25 [-0.70, 1.28]	47	0.56
tortuosity (DM) – interquartile range	0.1673	1.10 ± 0.18	1.72 ± 1.15	-0.64 [-1.06, 0.03]	58	0.70	0.0491	1.05 ± 0.54	1.25 ± 0.51	-0.36 [-1.71, 0.79]	55	0.77	1.0000	1.33 ± 0.49	1.26 ± 0.36	0.17 [-0.93, 1.25]	58	0.50
tortuosity (DM) – range	0.0449	81.80 ± 48.42	39.93 ± 16.73	1.26 [-0.61, 2.58]	63	0.79	0.7871	30.90 ± 20.91	43.77 ± 49.76	-0.30 [-0.95, 0.66]	60	0.54	0.4421	46.95 ± 34.97	31.47 ± 22.54	0.52 [-0.56, 1.43]	42	0.61
tortuosity (SOAM) – mean (rad/mm)	0.1956	7.16 ± 1.23	9.57 ± 3.82	-0.73 [-1.31, -0.13]	58	0.69	0.0972	7.19 ± 1.80	8.70 ± 1.82	-0.80 [-1.61, 0.64]	75	0.73	0.5999	8.25 ± 1.66	8.55 ± 1.65	-0.17 [-1.16, 0.91]	58	0.58
tortuosity (SOAM) – variance (rad^2/mm^2)	0.1198	54.50 ± 14.71	75.27 ± 29.26	-0.79 [-1.42, 0.09]	58	0.73	0.2633	54.79 ± 14.34	65.00 ± 30.05	-0.39 [-1.35, 0.51]	60	0.66	0.6574	65.03 ± 26.49	70.39 ± 20.40	-0.22 [-1.21, 0.91]	58	0.57
tortuosity (SOAM) – skewness	0.1956	1.94 ± 0.33	1.58 ± 0.72	0.56 [-0.46, 1.17]	53	0.69	0.1087	1.89 ± 0.53	1.46 ± 0.54	0.78 [-0.17, 1.80]	63	0.73	0.9039	1.59 ± 0.47	1.52 ± 0.42	0.16 [-0.80, 1.19]	58	0.52

Feature	Measurement 1						Measurement 2						Measurement 3					
	p	pCR	non-pCR	Effect size	Acc (%)	AUC	p	pCR	non-pCR	Effect size	Acc (%)	AUC	p	pCR	non-pCR	Effect size	Acc (%)	AUC
tortuosity (SOAM) – kurtosis	0.4320	5.05 ± 2.07	3.88 ± 2.87	0.43 [-0.54, 1.36]	53	0.62	0.1518	4.79 ± 3.09	2.73 ± 2.71	0.68 [-0.28, 1.82]	68	0.70	1.0000	3.05 ± 2.31	2.73 ± 1.91	0.15 [-0.76, 1.20]	58	0.50
tortuosity (SOAM) – median (rad/mm)	0.1673	4.89 ± 0.97	7.64 ± 5.08	-0.64 [-1.13, 0.12]	58	0.70	0.0409	4.99 ± 2.20	6.64 ± 2.02	-0.76 [-1.70, 0.43]	65	0.78	0.5999	5.70 ± 1.38	5.97 ± 1.50	-0.18 [-1.04, 0.73]	58	0.58
tortuosity (SOAM) – interquartile range (rad/mm)	0.1422	8.00 ± 1.85	10.09 ± 2.96	-0.76 [-1.43, 0.34]	58	0.71	0.0972	7.90 ± 2.04	9.21 ± 3.39	-0.43 [-1.56, 0.52]	60	0.73	0.4421	8.91 ± 2.31	10.40 ± 3.12	-0.48 [-1.19, 0.61]	58	0.61
tortuosity (SOAM) – range (rad/mm)	0.7108	51.50 ± 3.22	50.38 ± 6.69	0.19 [-0.67, 1.01]	58	0.56	0.5120	44.70 ± 8.15	44.58 ± 19.92	0.01 [-0.89, 0.84]	60	0.59	0.9039	44.38 ± 12.15	46.06 ± 8.37	-0.16 [-1.65, 0.72]	58	0.52
velocity – mean (mm/s)	0.4824	2.41 ± 0.18	2.24 ± 0.42	0.44 [-0.48, 1.11]	58	0.61	0.0826	2.41 ± 0.31	2.14 ± 0.29	0.85 [-0.16, 1.64]	60	0.74	1.0000	2.19 ± 0.39	2.18 ± 0.35	0.02 [-1.09, 1.00]	58	0.50
velocity – variance (mm^2/s^2)	0.2991	2.37 ± 0.21	2.43 ± 0.64	-0.12 [-1.01, 0.96]	58	0.65	0.2030	2.48 ± 0.27	2.09 ± 0.72	0.62 [-0.28, 1.14]	60	0.68	0.2375	2.11 ± 0.45	2.31 ± 0.48	-0.41 [-1.45, 0.57]	58	0.67
velocity – skewness	0.2614	0.73 ± 0.12	0.94 ± 0.41	-0.57 [-1.09, 0.28]	42	0.67	0.3348	0.76 ± 0.24	0.87 ± 0.40	-0.32 [-1.26, 0.63]	55	0.64	1.0000	0.84 ± 0.20	0.89 ± 0.29	-0.19 [-1.13, 0.77]	58	0.50
velocity – kurtosis	0.6504	-0.18 ± 0.20	0.33 ± 1.62	-0.37 [-0.94, 0.34]	58	0.57	0.2030	-0.19 ± 0.42	0.10 ± 0.82	-0.40 [-1.34, 0.50]	55	0.68	0.7780	0.03 ± 0.39	0.11 ± 0.69	-0.14 [-0.95, 0.89]	58	0.55
velocity – median (mm/s)	0.2268	2.09 ± 0.23	1.86 ± 0.40	0.63 [-0.28, 1.38]	58	0.68	0.2030	2.04 ± 0.41	1.77 ± 0.37	0.67 [-0.31, 1.62]	60	0.68	0.7780	1.87 ± 0.46	1.78 ± 0.39	0.20 [-0.83, 1.27]	58	0.55
velocity – interquartile range (mm/s)	1.0000	2.25 ± 0.13	2.17 ± 0.59	0.16 [-0.72, 0.92]	58	0.50	0.1535	2.26 ± 0.24	1.88 ± 0.59	0.74 [-0.02, 1.36]	60	0.70	0.5448	2.02 ± 0.32	2.12 ± 0.33	-0.30 [-1.20, 0.55]	58	0.59
velocity – range (mm/s)	0.7732	6.94 ± 0.09	6.63 ± 0.82	0.45 [-0.70, 0.93]	58	0.55	0.6160	6.79 ± 0.33	6.04 ± 1.93	0.47 [-0.60, 0.88]	60	0.57	0.6574	6.41 ± 0.96	6.69 ± 0.48	-0.38 [-1.65, 0.74]	58	0.57
velocity range – mean (mm/s)	0.1673	2.23 ± 0.30	1.91 ± 0.51	0.70 [-0.31, 1.29]	58	0.70	0.1137	2.17 ± 0.34	1.90 ± 0.35	0.75 [-0.26, 1.64]	60	0.72	0.5999	1.99 ± 0.36	1.84 ± 0.37	0.39 [-0.67, 1.30]	58	0.58
velocity range – variance (mm^2/s^2)	0.5358	2.11 ± 0.31	1.83 ± 0.71	0.45 [-0.52, 1.05]	58	0.60	0.2030	2.09 ± 0.44	1.65 ± 0.70	0.69 [-0.23, 1.42]	60	0.68	0.5999	1.85 ± 0.65	1.76 ± 0.55	0.14 [-0.99, 1.11]	58	0.58

Feature	Measurement 1						Measurement 2						Measurement 3					
	p	pCR	non-pCR	Effect size	Acc (%)	AUC	p	pCR	non-pCR	Effect size	Acc (%)	AUC	p	pCR	non-pCR	Effect size	Acc (%)	AUC
velocity range – skewness	0.0556	0.77 ± 0.20	1.15 ± 0.50	-0.88 [-1.42, -0.17]	53	0.77	0.0259	0.88 ± 0.25	1.26 ± 0.40	-1.05 [-1.80, -0.19]	74	0.81	0.3950	0.91 ± 0.40	1.07 ± 0.29	-0.46 [-1.44, 0.68]	37	0.63
velocity range – kurtosis	0.0449	-0.02 ± 0.51	1.31 ± 2.21	-0.70 [-1.17, 0.05]	58	0.79	0.0259	0.17 ± 0.62	1.53 ± 1.67	-0.97 [-1.64, -0.22]	47	0.81	0.3950	0.23 ± 1.05	0.68 ± 1.00	-0.42 [-1.46, 0.54]	42	0.63
velocity range – median (mm/s)	0.0683	1.94 ± 0.34	1.57 ± 0.43	0.88 [-0.15, 1.69]	58	0.76	0.1535	1.82 ± 0.38	1.54 ± 0.42	0.68 [-0.39, 1.58]	60	0.70	0.4920	1.64 ± 0.38	1.49 ± 0.34	0.39 [-0.59, 1.38]	58	0.60
velocity range – interquartile range (mm/s)	0.2268	2.09 ± 0.25	1.74 ± 0.61	0.65 [-0.19, 1.22]	58	0.68	0.2030	2.00 ± 0.39	1.57 ± 0.64	0.74 [-0.09, 1.36]	60	0.68	0.3950	1.85 ± 0.50	1.66 ± 0.43	0.38 [-0.73, 1.49]	58	0.63
velocity range – range (mm/s)	0.5918	6.81 ± 0.23	6.31 ± 1.17	0.50 [-0.23, 1.06]	58	0.58	0.6713	6.47 ± 0.70	5.87 ± 1.94	0.36 [-0.46, 1.03]	60	0.56	0.3511	5.85 ± 1.45	6.11 ± 1.34	-0.18 [-1.24, 0.57]	58	0.64
fractal dimension	0.0449	1.44 ± 0.15	1.23 ± 0.26	0.90 [0.04, 1.59]	37	0.79	0.2976	1.29 ± 0.21	1.16 ± 0.30	0.47 [-0.43, 1.21]	50	0.65	0.9039	1.24 ± 0.25	1.23 ± 0.22	0.04 [-0.93, 1.00]	58	0.52
fractal R^2	0.0221	1.00 ± $2.77.10^{-3}$	0.98 ± 0.04	0.46 [-0.37, 0.88]	37	0.82	0.9079	0.99 ± $2.03.10^{-3}$	0.97 ± 0.09	0.37 [-0.63, 0.75]	60	0.52	1.0000	0.99 ± $4.08.10^{-3}$	0.99 ± 0.01	0.36 [-0.76, 1.07]	58	0.50

Supplementary Table 9: Summary statistics of ULM-based features for changes between measurements. Delta radiomic features of changes include relative change, absolute change, minimum, and maximum of the two measurements in question. From left to right: feature name, p-value for a statistical significance between patients with pCR and non-pCR (significant features are highlighted in light blue, significant features after Benjamini-Hochberg correction with a false discovery rate of 5% or 10% are highlighted in dark or medium blue, respectively), mean ± standard deviation of patients with pCR, mean ± standard deviation of patients with non-pCR, effect size expressed by Hedges' g with bootstrapped 95% confidence interval, cross-validated predictive accuracy, area under the receiver operating characteristic curve (AUC).

	Measurement 1 → 2						Measurement 2 → 3						Measurement 1 → 3					
**Feature**	**p**	**pCR**	**non-pCR**	**Effect size**	**Acc (%)**	**AUC**	**p**	**pCR**	**non-pCR**	**Effect size**	**Acc (%)**	**AUC**	**p**	**pCR**	**non-pCR**	**Effect size**	**Acc (%)**	**AUC**
relative change (ΔF)																		
compensated vessel coverage (%)	0.5918	-9.42 ± 31.52	-0.88 ± 10.03	-0.40 [-2.58, 1.25]	53	0.58	0.2060	-7.74 ± 32.34	2.30 ± 14.62	-0.41 [-1.41, 1.07]	53	0.68	0.3283	-10.65 ± 25.04	-0.06 ± 16.91	-0.50 [-1.58, 0.95]	53	0.65
distances – mean (mm)	0.9079	0.46 ± 0.99	0.16 ± 1.12	0.26 [-0.73, 1.28]	60	0.60	0.6160	0.18 ± 1.40	-0.50 ± 1.67	0.41 [-0.72, 1.21]	60	0.63	0.9079	0.55 ± 1.47	-0.31 ± 1.18	0.64 [-0.27, 1.69]	55	0.65
distances – variance (mm^2)	0.7108	0.43 ± 0.96	0.59 ± 1.87	-0.09 [-1.01, 0.89]	58	0.56	0.3100	0.78 ± 2.99	-0.98 ± 2.14	0.66 [-0.21, 1.46]	56	0.65	0.4252	1.28 ± 3.40	-0.33 ± 2.00	0.59 [-0.35, 1.57]	58	0.62
distances – skewness	0.8369	-0.16 ± 1.60	-0.12 ± 0.81	-0.03 [-1.40, 1.41]	58	0.54	0.3511	-0.78 ± 1.97	0.30 ± 0.96	-0.70 [-1.81, 0.45]	68	0.64	0.2854	-0.43 ± 1.11	0.14 ± 1.30	-0.44 [-1.52, 0.54]	68	0.66
distances – kurtosis	0.9018	0.13 ± 8.98	-1.00 ± 5.51	0.16 [-1.06, 1.41]	53	0.52	0.2723	-5.13 ± 10.23	1.37 ± 3.42	-0.88 [-1.97, 0.23]	68	0.66	0.5962	-2.30 ± 5.55	-0.02 ± 7.23	-0.33 [-1.28, 0.75]	68	0.58
distances – median (mm)	0.7871	0.43 ± 0.84	0.10 ± 1.16	0.30 [-0.60, 1.21]	60	0.62	0.7285	0.12 ± 1.25	-0.44 ± 1.67	0.35 [-0.60, 1.09]	60	0.60	0.8471	0.46 ± 1.15	-0.33 ± 1.14	0.66 [-0.20, 1.64]	55	0.66
distances – interquartile range (mm)	0.4320	0.40 ± 1.19	0.14 ± 0.88	0.25 [-0.80, 1.47]	58	0.62	0.4421	0.39 ± 1.83	-0.47 ± 1.36	0.52 [-0.49, 1.49]	47	0.61	0.3283	0.71 ± 2.09	-0.30 ± 1.07	0.63 [-0.27, 1.71]	47	0.65
distances – range (mm)	0.3402	1.57 ± 3.01	0.36 ± 2.40	0.44 [-0.83, 1.48]	53	0.64	0.4920	0.02 ± 3.44	-0.92 ± 3.03	0.28 [-0.80, 1.16]	58	0.60	0.3749	1.57 ± 4.25	-0.49 ± 3.11	0.55 [-0.41, 1.64]	58	0.64
flow direction – variance (rad^2)	0.5358	-0.10 ± 0.34	-0.14 ± 0.60	0.07 [-0.93, 1.05]	58	0.60	0.3511	0.11 ± 0.45	0.42 ± 0.65	-0.51 [-1.35, 0.43]	53	0.64	1.0000	0.11 ± 0.34	0.21 ± 0.74	-0.16 [-0.90, 0.88]	50	0.51
track length – mean (mm)	0.3845	-0.04 ± 0.16	0.01 ± 0.11	-0.35 [-1.64, 0.87]	58	0.63	0.6574	-0.05 ± 0.20	0.00 ± 0.15	-0.27 [-1.26, 0.72]	56	0.57	0.4252	-0.03 ± 0.18	0.01 ± 0.20	-0.22 [-1.25, 0.80]	58	0.62
track length – variance (mm^2)	0.7732	-0.03 ± 0.15	-1.72.10^{-3} ± 0.08	-0.27 [-1.54, 0.96]	58	0.55	0.9039	-0.01 ± 0.17	0.04 ± 0.15	-0.30 [-1.38, 0.63]	56	0.52	0.5360	3.66.10^{-3} ± 0.17	0.04 ± 0.17	-0.21 [-1.39, 0.70]	58	0.60

Feature	Measurement 1 → 2						Measurement 2 → 3						Measurement 1 → 3					
	p	pCR	non-pCR	Effect size	Acc (%)	AUC	p	pCR	non-pCR	Effect size	Acc (%)	AUC	p	pCR	non-pCR	Effect size	Acc (%)	AUC
track length – skewness	0.4789	0.22 ± 0.66	0.03 ± 0.58	0.29 [-0.81, 1.32]	44	0.61	0.1728	-0.08 ± 0.58	0.37 ± 0.94	-0.54 [-1.26, 0.61]	44	0.70	0.6590	0.14 ± 0.66	0.42 ± 0.66	-0.41 [-1.51, 0.48]	39	0.57
track length – kurtosis	0.5360	1.44 ± 4.65	-0.09 ± 3.96	0.34 [-0.70, 1.50]	44	0.60	0.2370	-1.21 ± 3.79	3.04 ± 7.47	-0.66 [-1.37, 0.37]	56	0.68	0.3749	0.32 ± 3.97	3.11 ± 5.78	-0.51 [-1.31, 0.57]	56	0.64
track length – median (mm)	0.6504	-0.02 ± 0.14	$-4.95.10^{-3}$ ± 0.12	-0.13 [-1.10, 1.10]	58	0.57	0.9039	-0.05 ± 0.16	-0.01 ± 0.11	-0.29 [-1.39, 0.67]	56	0.52	0.7242	-0.04 ± 0.16	-0.02 ± 0.17	-0.11 [-1.08, 0.98]	58	0.56
track length – interquartile range (mm)	0.5918	-0.03 ± 0.11	-0.01 ± 0.11	-0.17 [-1.13, 0.97]	58	0.58	0.7168	-0.03 ± 0.19	0.02 ± 0.21	-0.26 [-1.24, 0.66]	56	0.56	0.7242	-0.02 ± 0.16	$4.18.10^{-3}$± 0.19	-0.13 [-1.18, 0.76]	58	0.56
track length – range (mm)	0.4824	-0.81 ± 2.13	-0.27 ± 1.03	-0.34 [-1.73, 1.15]	58	0.61	0.3100	-0.30 ± 2.20	0.84 ± 1.79	-0.55 [-1.58, 0.45]	44	0.65	0.2109	-0.62 ± 2.06	0.65 ± 1.58	-0.68 [-1.81, 0.39]	47	0.69
tortuosity (DM) – mean	0.3402	0.03 ± 0.87	0.07 ± 0.86	-0.05 [-1.04, 1.08]	53	0.64	0.0620	0.32 ± 1.00	-0.51 ± 1.38	0.64 [-0.64, 1.31]	63	0.76	0.0556	0.36 ± 0.49	-0.43 ± 0.95	0.93 [0.29, 1.68]	63	0.78
tortuosity (DM) – variance	0.4824	-5.01 ± 14.64	-0.58 ± 16.16	-0.27 [-1.12, 0.77]	53	0.61	0.0409	10.44 ± 18.11	-3.73 ± 13.43	0.87 [0.09, 1.65]	58	0.78	0.3283	7.88 ± 22.86	-4.22 ± 10.01	0.72 [-0.57, 1.93]	63	0.65
tortuosity (DM) – skewness	0.0268	-7.15 ± 7.16	0.42 ± 6.12	-1.10 [-1.94, 0.20]	61	0.82	0.5726	0.79 ± 5.86	-1.62 ± 6.03	0.39 [-0.76, 1.23]	56	0.59	0.1042	-4.98 ± 7.49	-0.75 ± 4.55	-0.69 [-1.63, 0.44]	50	0.74
tortuosity (DM) – kurtosis	0.0204	$-2.37.10^{2}$ ± $2.70.10^{2}$	38.69 ± $1.77.10^{2}$	-1.21 [-1.99, -0.41]	72	0.83	0.4598	22.97 ± 120.58	-57.51 ± 174.56	0.50 [-0.63, 1.16]	61	0.61	0.0556	$-1.87.10^{2}$ ± $2.73.10^{2}$	-9.53 ± $1.08.10^{2}$	-0.90 [-1.62, 0.50]	61	0.78
tortuosity (DM) – median	0.2991	-0.01 ± 0.22	0.23 ± 1.27	-0.22 [-1.66, 0.62]	53	0.65	0.0908	0.12 ± 0.30	-0.47 ± 1.64	0.45 [-0.61, 0.91]	56	0.74	0.4252	0.08 ± 0.18	-0.22 ± 0.55	0.64 [-0.09, 1.37]	47	0.62
tortuosity (DM) – interquartile range	0.3845	-0.04 ± 0.67	-0.48 ± 1.52	0.32 [-0.58, 1.01]	53	0.63	0.2723	0.28 ± 0.86	0.01 ± 0.66	0.34 [-0.81, 1.46]	56	0.66	0.2854	0.28 ± 0.41	-0.52 ± 1.35	0.69 [-0.11, 1.28]	50	0.66
tortuosity (DM) – range	0.0171	-57.76 ± 50.82	3.84 ± 51.10	-1.15 [-1.91, -0.10]	79	0.83	0.1288	16.05 ± 44.88	-14.36 ± 50.35	0.60 [-0.56, 1.31]	56	0.72	0.3749	-29.14 ± 63.29	-9.02 ± 29.07	-0.43 [-1.77, 0.90]	56	0.64
tortuosity (SOAM) – mean (rad/mm)	0.9671	0.23 ± 2.81	-0.87 ± 4.15	0.28 [-0.76, 1.06]	58	0.51	0.3100	1.06 ± 2.90	-0.20 ± 2.77	0.43 [-0.68, 1.41]	47	0.65	0.4789	1.20 ± 2.02	-1.24 ± 4.85	0.58 [-0.37, 1.27]	58	0.61
tortuosity (SOAM) – variance (rad^2/mm^2)	0.7108	2.31 ± 26.39	-10.27 ± 47.66	0.29 [-0.66, 1.04]	58	0.56	0.9678	10.25 ± 27.24	6.01 ± 36.70	0.12 [-0.80, 1.07]	58	0.51	0.2109	16.70 ± 28.64	-6.75 ± 43.23	0.58 [-0.35, 1.51]	56	0.69

	**Measurement 1 → 2**						**Measurement 2 → 3**						**Measurement 1 → 3**					
**Feature**	**p**	**pCR**	**non-pCR**	**Effect size**	**Acc (%)**	**AUC**	**p**	**pCR**	**non-pCR**	**Effect size**	**Acc (%)**	**AUC**	**p**	**pCR**	**non-pCR**	**Effect size**	**Acc (%)**	**AUC**
tortuosity (SOAM) – skewness	0.4789	-0.12 ± 0.76	-0.29 ± 0.52	0.26 [-1.09, 1.52]	44	0.61	0.3599	-0.30 ± 0.93	0.07 ± 0.67	-0.44 [-1.56, 0.80]	56	0.64	0.8601	-0.24 ± 0.31	-0.02 ± 0.95	-0.27 [-1.13, 0.59]	56	0.53
tortuosity (SOAM) – kurtosis	0.7914	-0.77 ± 4.38	-1.58 ± 3.48	0.20 [-1.26, 1.23]	56	0.55	0.3599	-1.74 ± 4.83	-0.03 ± 2.91	-0.42 [-1.52, 0.61]	56	0.64	0.9298	-1.62 ± 1.83	-1.03 ± 3.79	-0.18 [-0.99, 0.77]	56	0.52
tortuosity (SOAM) – median (rad/mm)	0.6504	0.27 ± 3.00	-0.99 ± 4.74	0.29 [-0.79, 1.13]	58	0.57	0.2723	0.71 ± 3.21	-0.79 ± 2.89	0.47 [-0.65, 1.43]	42	0.66	0.6590	0.66 ± 1.47	-1.92 ± 6.12	0.50 [-0.22, 1.24]	63	0.57
tortuosity (SOAM) – interquartile range (rad/mm)	0.9671	0.19 ± 3.58	-0.88 ± 5.41	0.21 [-0.85, 0.94]	58	0.51	0.5448	1.02 ± 3.22	1.12 ± 4.68	-0.02 [-0.94, 0.99]	58	0.59	0.6590	1.39 ± 2.70	0.02 ± 5.19	0.29 [-0.74, 1.06]	56	0.57
tortuosity (SOAM) – range (rad/mm)	0.5358	-7.74 ± 10.30	-5.80 ± 20.12	-0.11 [-1.04, 0.78]	53	0.60	0.9039	-0.32 ± 15.86	2.66 ± 18.46	-0.16 [-0.98, 0.72]	58	0.52	0.8601	-3.35 ± 6.27	-4.44 ± 1.22	0.10 [-0.79, 0.85]	56	0.53
velocity – mean (mm/s)	0.3845	-0.04 ± 0.38	-0.10 ± 0.49	0.13 [-0.73, 1.18]	58	0.63	0.3511	-0.22 ± 0.57	0.06 ± 0.39	-0.56 [-1.56, 0.54]	58	0.64	0.9298	-0.15 ± 0.41	-0.05 ± 0.65	-0.15 [-0.96, 0.80]	58	0.52
velocity – variance (mm^2/s^2)	0.0449	0.15 ± 0.25	-0.34 ± 0.49	1.11 [0.29, 1.87]	58	0.79	0.0754	-0.36 ± 0.38	0.25 ± 0.91	-0.78 [-1.36, 0.00]	58	0.75	0.5962	-0.13 ± 0.29	-0.13 ± 0.86	0.00 [-0.94, 0.91]	58	0.58
velocity – skewness	0.8369	0.06 ± 0.29	-0.06 ± 0.75	0.19 [-0.81, 1.06]	58	0.54	0.9678	0.08 ± 0.41	0.01 ± 0.43	0.17 [-0.81, 1.19]	56	0.51	0.6590	0.08 ± 0.23	-0.06 ± 0.62	0.25 [-0.63, 1.08]	56	0.57
velocity – kurtosis	0.2991	0.02 ± 0.48	-0.23 ± 2.26	0.13 [-1.03, 0.95]	58	0.65	0.9039	0.22 ± 0.73	-0.01 ± 0.95	0.26 [-0.72, 1.21]	50	0.52	0.7242	0.15 ± 0.46	-0.25 ± 2.00	0.23 [-0.65, 0.98]	56	0.56
velocity – median (mm/s)	0.7732	-0.10 ± 0.52	-0.08 ± 0.59	-0.03 [-1.00, 0.94]	58	0.55	0.5999	-0.17 ± 0.72	0.03 ± 0.45	-0.34 [-1.46, 0.83]	50	0.58	0.9298	-0.15 ± 0.51	-0.07 ± 0.71	-0.12 [-1.01, 0.96]	58	0.52
velocity – interquartile range (mm/s)	0.1422	0.02 ± 0.22	-0.28 ± 0.39	0.83 [0.10, 1.63]	58	0.71	0.0754	-0.24 ± 0.41	0.27 ± 0.71	-0.80 [-1.45, 0.04]	68	0.75	0.7914	-0.13 ± 0.24	-0.05 ± 0.68	-0.15 [-1.00, 0.78]	58	0.55
velocity – range (mm/s)	0.5358	-0.17 ± 0.41	-0.59 ± 1.18	0.40 [-0.51, 0.89]	58	0.60	0.2060	-0.38 ± 1.09	0.73 ± 2.03	-0.62 [-1.28, 0.13]	56	0.68	0.7242	-0.33 ± 0.85	0.09 ± 0.90	-0.45 [-1.70, 0.79]	58	0.56
velocity range – mean (mm/s)	0.9671	-0.13 ± 0.49	-0.01 ± 0.58	-0.21 [-1.07, 0.81]	58	0.51	0.5999	-0.18 ± 0.60	-0.03 ± 0.39	-0.29 [-1.27, 0.83]	56	0.58	0.5962	-0.20 ± 0.52	-0.04 ± 0.67	-0.25 [-1.16, 0.82]	58	0.58

Feature	Measurement 1 → 2						Measurement 2 → 3						Measurement 1 → 3					
	p	pCR	non-pCR	Effect size	Acc (%)	AUC	p	pCR	non-pCR	Effect size	Acc (%)	AUC	p	pCR	non-pCR	Effect size	Acc (%)	AUC
velocity range – variance (mm^2/s^2)	0.6504	-0.07 ± 0.52	-0.18 ± 0.41	0.23 [-0.85, 1.28]	58	0.57	0.7168	-0.25 ± 0.92	0.16 ± 0.99	-0.41 [-1.22, 0.50]	56	0.56	0.9298	-0.10 ± 0.70	-0.05 ± 0.94	-0.06 [-0.95, 0.94]	58	0.52
velocity range – skewness	0.7242	0.17 ± 0.38	0.22 ± 0.32	-0.15 [-1.31, 1.01]	56	0.56	0.5726	0.02 ± 0.45	-0.19 ± 0.53	0.42 [-0.59, 1.34]	56	0.59	0.3749	0.23 ± 0.37	-0.11 ± 0.74	0.52 [-0.31, 1.23]	56	0.64
velocity range – kurtosis	0.4252	0.30 ± 0.92	0.76 ± 1.15	-0.42 [-1.21, 0.55]	56	0.62	0.4082	0.06 ± 1.19	-0.91 ± 2.15	0.51 [-0.40, 1.38]	56	0.63	0.5360	0.47 ± 1.06	-0.75 ± 2.96	0.48 [-0.33, 1.23]	56	0.60
velocity range – median (mm/s)	0.9671	-0.20 ± 0.55	-0.03 ± 0.62	-0.27 [-1.22, 0.88]	58	0.51	0.7168	-0.19 ± 0.66	-0.03 ± 0.37	-0.30 [-1.34, 0.93]	56	0.56	0.5962	-0.25 ± 0.56	-0.05 ± 0.60	-0.33 [-1.14, 0.72]	58	0.58
velocity range – interquartile range (mm/s)	0.7732	-0.15 ± 0.47	-0.17 ± 0.34	0.06 [-1.15, 1.23]	58	0.55	0.7168	-0.15 ± 0.74	0.14 ± 0.93	-0.32 [-1.15, 0.57]	50	0.56	0.8601	-0.18 ± 0.64	-0.05 ± 0.93	-0.15 [-1.10, 0.84]	58	0.53
velocity range – range (mm/s)	0.9018	-0.43 ± 0.85	-0.44 ± 1.02	0.00 [-1.11, 0.78]	58	0.52	0.4920	-0.62 ± 1.88	0.32 ± 2.39	-0.41 [-1.21, 0.54]	56	0.60	0.6590	-0.50 ± 0.84	-0.17 ± 1.55	-0.24 [-1.09, 0.79]	50	0.57
fractal dimension	0.4320	-0.19 ± 0.32	-0.06 ± 0.18	-0.49 [-1.90, 1.02]	42	0.62	0.3100	-0.06 ± 0.38	0.10 ± 0.36	-0.40 [-1.29, 0.69]	53	0.65	0.1791	-0.16 ± 0.32	0.03 ± 0.34	-0.54 [-1.47, 0.43]	53	0.70
fractal R^2	0.7732	-2.05.10^{-3} ± 2.08.10^{-3}	-0.01 ± 0.08	0.15 [-0.63, 0.86]	47	0.55	0.8404	-7.78.10^{-4} ± 3.12.10^{-3}	0.02 ± 0.09	-0.34 [-1.53, 0.57]	53	0.53	0.3749	-1.96.10^{-3} ± 2.51.10^{-3}	0.01 ± 0.04	-0.42 [-1.02, 0.50]	53	0.64
absolute change (|ΔF|)																		
compensated vessel coverage (%)	0.0005	28.39 ± 12.52	7.50 ± 6.34	2.21 [0.48, 3.43]	84	0.95	0.0506	25.67 ± 19.00	9.50 ± 10.96	1.04 [0.01, 2.29]	68	0.77	0.1791	21.25 ± 15.25	11.49 ± 11.86	0.70 [-0.36, 1.96]	68	0.70
distances – mean (mm)	0.2030	0.89 ± 0.55	0.73 ± 0.84	0.19 [-0.77, 1.24]	65	0.63	0.7285	1.02 ± 0.90	0.95 ± 1.44	0.05 [-0.92, 1.14]	60	0.60	0.5369	0.88 ± 1.27	0.92 ± 0.75	-0.04 [-1.18, 1.57]	55	0.64
distances – variance (mm^2)	0.8369	0.85 ± 0.53	1.22 ± 1.50	-0.28 [-1.05, 0.53]	58	0.54	0.9678	1.55 ± 2.62	1.47 ± 1.80	0.03 [-0.84, 1.19]	58	0.51	0.4789	1.62 ± 3.23	1.34 ± 1.46	0.11 [-1.00, 2.79]	58	0.61
distances – skewness	0.0018	1.41 ± 0.52	0.58 ± 0.54	1.49 [0.15, 2.88]	68	0.92	0.0409	1.59 ± 1.31	0.64 ± 0.76	0.89 [-0.34, 1.85]	68	0.78	0.9298	0.99 ± 0.57	1.03 ± 0.74	-0.06 [-0.89, 0.98]	68	0.52
distances – kurtosis	0.0072	7.67 ± 3.47	3.12 ± 4.57	1.03 [-0.24, 2.74]	63	0.87	0.0259	8.05 ± 7.78	2.44 ± 2.68	0.99 [0.03, 1.94]	68	0.81	0.3749	5.04 ± 2.72	4.94 ± 5.04	0.02 [-0.89, 0.94]	74	0.64

Feature	Measurement 1 → 2						Measurement 2 → 3						Measurement 1 → 3					
	p	pCR	non-pCR	Effect size	Acc (%)	AUC	p	pCR	non-pCR	Effect size	Acc (%)	AUC	p	pCR	non-pCR	Effect size	Acc (%)	AUC
distances – median (mm)	0.2318	0.72 ± 0.56	0.71 ± 0.90	0.01 [-0.70, 1.19]	60	0.62	0.6160	0.91 ± 0.80	0.85 ± 1.49	0.04 [-0.87, 1.18]	60	0.63	0.4402	0.67 ± 1.02	0.87 ± 0.77	-0.22 [-1.17, 1.31]	55	0.66
distances – interquartile range (mm)	0.1198	1.05 ± 0.56	0.58 ± 0.66	0.72 [-0.41, 2.26]	58	0.73	0.3950	1.29 ± 1.27	0.91 ± 1.10	0.32 [-0.68, 1.28]	56	0.63	0.7242	1.25 ± 1.76	0.90 ± 0.59	0.28 [-1.04, 2.69]	58	0.56
distances – range (mm)	0.1956	2.75 ± 1.77	1.78 ± 1.56	0.56 [-0.59, 1.62]	58	0.69	1.0000	2.35 ± 2.36	1.96 ± 2.42	0.15 [-0.94, 1.22]	56	0.50	0.7914	2.82 ± 3.42	2.52 ± 1.73	0.12 [-1.00, 2.09]	53	0.55
flow direction – variance (rad^2)	0.7732	0.28 ± 0.18	0.38 ± 0.48	-0.22 [-0.97, 0.74]	58	0.55	0.9678	0.37 ± 0.25	0.54 ± 0.54	-0.37 [-1.28, 0.49]	58	0.51	1.0000	0.30 ± 0.16	0.49 ± 0.58	-0.39 [-1.07, 0.51]	58	0.51
track length – mean (mm)	0.0556	0.14 ± 0.04	0.08 ± 0.07	0.99 [0.08, 2.08]	58	0.77	0.3950	0.16 ± 0.11	0.11 ± 0.09	0.45 [-0.52, 1.45]	56	0.63	0.8601	0.12 ± 0.13	0.14 ± 0.14	-0.10 [-0.91, 1.01]	58	0.53
track length – variance (mm^2)	0.1956	0.11 ± 0.08	0.06 ± 0.04	0.80 [-0.41, 2.22]	58	0.69	0.3511	0.14 ± 0.09	0.11 ± 0.10	0.29 [-0.62, 1.29]	56	0.64	1.0000	0.12 ± 0.10	0.13 ± 0.10	-0.11 [-0.96, 0.90]	58	0.51
track length – skewness	0.5962	0.57 ± 0.34	0.46 ± 0.32	0.32 [-0.83, 1.24]	50	0.58	0.4598	0.39 ± 0.41	0.66 ± 0.74	-0.42 [-1.22, 0.59]	56	0.61	1.0000	0.49 ± 0.41	0.53 ± 0.57	-0.07 [-0.98, 1.08]	56	0.51
track length – kurtosis	0.3749	4.16 ± 1.96	3.31 ± 1.90	0.42 [-0.66, 1.38]	39	0.64	0.5148	2.96 ± 2.46	5.21 ± 6.00	-0.45 [-1.19, 0.51]	56	0.60	1.0000	2.96 ± 2.37	3.85 ± 5.27	-0.19 [-1.07, 1.00]	50	0.51
track length – median (mm)	0.1003	0.13 ± 0.03	0.09 ± 0.07	0.65 [-0.38, 1.56]	58	0.74	0.3511	0.13 ± 0.09	0.09 ± 0.06	0.55 [-0.42, 1.58]	56	0.64	0.7914	0.12 ± 0.11	0.13 ± 0.11	-0.14 [-1.07, 0.89]	58	0.55
track length – interquartile range (mm)	0.3845	0.10 ± 0.04	0.08 ± 0.06	0.22 [-0.66, 1.20]	58	0.63	0.4920	0.16 ± 0.09	0.14 ± 0.15	0.11 [-0.85, 1.03]	58	0.60	0.5360	0.11 ± 0.11	0.14 ± 0.12	-0.29 [-1.10, 0.87]	58	0.60
track length – range (mm)	0.0171	1.94 ± 0.96	0.91 ± 0.48	1.43 [0.23, 2.46]	74	0.83	0.9678	1.66 ± 1.34	1.48 ± 1.25	0.14 [-0.78, 1.20]	56	0.51	0.8601	1.69 ± 1.18	1.41 ± 0.88	0.26 [-0.83, 1.32]	58	0.53
tortuosity (DM) – mean	0.8369	0.53 ± 0.65	0.60 ± 0.60	-0.11 [-0.89, 1.18]	58	0.54	0.5999	0.75 ± 0.68	0.72 ± 1.26	0.02 [-0.93, 1.15]	56	0.58	0.9298	0.44 ± 0.41	0.65 ± 0.79	-0.30 [-0.96, 0.77]	53	0.52
tortuosity (DM) – variance	0.5358	11.98 ± 8.73	10.99 ± 11.39	0.09 [-0.77, 1.17]	47	0.60	0.9678	12.32 ± 16.71	9.34 ± 9.98	0.22 [-0.73, 1.28]	61	0.51	0.1042	17.31 ± 15.67	7.33 ± 7.78	0.83 [-0.15, 1.86]	61	0.74
tortuosity (DM) – skewness	0.0693	8.08 ± 5.89	4.24 ± 4.23	0.74 [-0.21, 1.87]	39	0.77	0.5148	3.57 ± 4.53	3.97 ± 4.66	-0.08 [-0.96, 1.08]	56	0.60	0.2854	6.47 ± 6.01	3.69 ± 2.52	0.63 [-0.41, 1.53]	56	0.66

Feature	Measurement 1 → 2						Measurement 2 → 3						Measurement 1 → 3					
	p	pCR	non-pCR	Effect size	Acc (%)	AUC	p	pCR	non-pCR	Effect size	Acc (%)	AUC	p	pCR	non-pCR	Effect size	Acc (%)	AUC
tortuosity (DM) – kurtosis	0.0693	$2.50.10^2$ ± $2.57.10^2$	$1.07.10^2$ ± $1.42.10^2$	0.70 [-0.38, 1.73]	61	0.77	0.8286	73.76 ± 94.47	90.89 ± $1.58.10^2$	-0.12 [-1.02, 0.85]	61	0.54	0.1042	$2.06.10^2$ ± $2.56.10^2$	75.98 ± 73.78	0.74 [-0.29, 1.63]	50	0.74
tortuosity (DM) – median	0.9671	0.15 ± 0.15	0.53 ± 1.16	-0.39 [-0.87, 0.67]	58	0.51	0.5448	0.24 ± 0.20	0.62 ± 1.58	-0.30 [-1.17, 0.92]	56	0.59	0.1791	0.11 ± 0.15	0.39 ± 0.44	-0.71 [-1.33, 0.13]	53	0.70
tortuosity (DM) – interquartile range	0.8369	0.46 ± 0.46	0.77 ± 1.38	-0.27 [-0.90, 0.84]	58	0.54	0.7168	0.66 ± 0.58	0.48 ± 0.43	0.34 [-0.72, 1.32]	58	0.56	0.3283	0.36 ± 0.33	0.90 ± 1.11	-0.57 [-1.15, 0.25]	56	0.65
tortuosity (DM) – range	0.0831	63.21 ± 42.60	34.52 ± 36.43	0.71 [-0.39, 1.73]	47	0.75	0.7780	34.22 ± 31.15	30.60 ± 41.62	0.09 [-0.89, 1.12]	67	0.55	0.0441	53.17 ± 41.20	23.16 ± 18.56	0.98 [-0.13, 1.84]	67	0.79
tortuosity (SOAM) – mean (mm^{-1})	0.2614	2.33 ± 1.28	2.42 ± 3.42	-0.03 [-0.71, 1.07]	58	0.67	0.1518	2.58 ± 1.46	1.84 ± 2.00	0.39 [-0.67, 1.55]	50	0.70	0.3283	1.80 ± 1.41	3.59 ± 3.33	-0.62 [-1.30, 0.27]	56	0.65
tortuosity (SOAM) – mean (rad/mm)	0.7732	22.48 ± 10.61	35.76 ± 31.49	-0.49 [-1.14, 0.57]	58	0.55	0.5999	25.06 ± 12.02	27.37 ± 23.72	-0.11 [-0.90, 0.96]	58	0.58	0.3283	24.27 ± 21.41	33.85 ± 25.66	-0.38 [-1.21, 0.73]	58	0.65
tortuosity (SOAM) – variance (rad^2/mm^2)	0.4789	0.58 ± 0.46	0.43 ± 0.40	0.34 [-0.60, 1.54]	56	0.61	0.3154	0.79 ± 0.50	0.55 ± 0.35	0.55 [-0.48, 1.58]	44	0.65	0.4252	0.33 ± 0.18	0.70 ± 0.61	-0.70 [-1.44, 0.06]	44	0.62
tortuosity (SOAM) – skewness	0.2109	3.55 ± 2.27	2.50 ± 2.82	0.38 [-0.69, 1.42]	50	0.69	0.1728	4.18 ± 2.62	2.39 ± 1.46	0.83 [-0.16, 2.00]	61	0.70	0.1791	2.05 ± 1.24	3.24 ± 2.00	-0.64 [-1.61, 0.22]	67	0.70
tortuosity (SOAM) – kurtosis	0.4320	2.18 ± 1.88	2.67 ± 3.97	-0.14 [-0.82, 1.05]	58	0.62	0.7168	2.55 ± 1.86	2.17 ± 1.96	0.19 [-0.76, 1.35]	56	0.56	0.3283	1.26 ± 0.91	4.08 ± 4.82	-0.70 [-1.30, 0.04]	53	0.65
tortuosity (SOAM) – median (rad/mm)	0.3402	2.98 ± 1.58	3.18 ± 4.36	-0.05 [-0.96, 0.95]	58	0.64	0.7780	2.73 ± 1.76	3.28 ± 3.38	-0.19 [-0.98, 0.82]	58	0.55	0.2463	2.25 ± 1.91	4.03 ± 3.02	-0.64 [-1.34, 0.38]	58	0.68
tortuosity (SOAM) – interquartile range (rad/mm)	0.1003	8.66 ± 9.41	15.82 ± 12.98	-0.58 [-1.36, 0.48]	58	0.74	0.9678	10.80 ± 10.87	11.60 ± 14.16	-0.06 [-0.92, 1.01]	58	0.51	0.0346	4.32 ± 5.53	9.98 ± 7.73	-0.77 [-1.54, 0.28]	53	0.81
velocity – mean (mm/s)	0.9671	0.31 ± 0.19	0.36 ± 0.34	-0.16 [-0.97, 0.77]	58	0.51	0.0908	0.51 ± 0.30	0.28 ± 0.27	0.78 [-0.33, 1.97]	74	0.74	0.5962	0.34 ± 0.23	0.48 ± 0.42	-0.34 [-1.06, 0.61]	58	0.58
velocity – variance (mm^2/s^2)	0.2991	0.22 ± 0.17	0.44 ± 0.39	-0.64 [-1.33, 0.11]	58	0.65	0.9678	0.39 ± 0.36	0.55 ± 0.76	-0.25 [-1.17, 0.73]	56	0.51	0.3283	0.25 ± 0.16	0.58 ± 0.62	-0.61 [-1.19, 0.06]	58	0.65

Feature	Measurement 1 → 2						Measurement 2 → 3						Measurement 1 → 3					
	p	pCR	non-pCR	Effect size	Acc (%)	AUC	p	pCR	non-pCR	Effect size	Acc (%)	AUC	p	pCR	non-pCR	Effect size	Acc (%)	AUC
velocity – skewness	0.8369	0.23 ± 0.16	0.40 ± 0.63	-0.31 [-1.01, 0.61]	53	0.54	0.9678	0.33 ± 0.23	0.30 ± 0.28	0.09 [-0.88, 1.11]	67	0.51	0.1509	0.19 ± 0.12	0.44 ± 0.41	-0.71 [-1.25, 0.16]	67	0.71
velocity – kurtosis	0.4320	0.37 ± 0.27	1.10 ± 1.97	-0.44 [-0.80, 0.43]	58	0.62	0.9039	0.58 ± 0.46	0.64 ± 0.66	-0.11 [-1.02, 0.91]	78	0.52	0.1259	0.38 ± 0.27	1.14 ± 1.63	-0.56 [-0.95, 0.36]	78	0.73
velocity – median (mm/s)	0.2991	0.43 ± 0.25	0.40 ± 0.42	0.09 [-0.73, 1.15]	58	0.65	0.0506	0.63 ± 0.32	0.30 ± 0.32	0.99 [-0.36, 2.46]	68	0.77	0.7914	0.43 ± 0.27	0.55 ± 0.42	-0.32 [-1.07, 0.74]	58	0.55
velocity – interquartile range (mm/s)	0.1673	0.15 ± 0.15	0.35 ± 0.33	-0.68 [-1.36, 0.05]	58	0.70	0.8404	0.34 ± 0.32	0.40 ± 0.64	-0.10 [-0.99, 0.95]	58	0.53	0.1042	0.20 ± 0.18	0.47 ± 0.48	-0.67 [-1.29, 0.41]	58	0.74
velocity – range (mm/s)	0.6504	0.30 ± 0.31	0.63 ± 1.15	-0.33 [-0.94, 0.51]	58	0.57	0.7780	0.73 ± 0.87	0.89 ± 1.96	-0.09 [-0.96, 0.94]	56	0.55	0.8601	0.41 ± 0.81	0.45 ± 0.77	-0.04 [-0.83, 1.09]	56	0.53
velocity range – mean (mm/s)	0.2991	0.42 ± 0.24	0.35 ± 0.44	0.16 [-0.76, 1.28]	58	0.65	0.1774	0.50 ± 0.33	0.31 ± 0.23	0.67 [-0.36, 1.82]	56	0.69	1.0000	0.44 ± 0.29	0.51 ± 0.40	-0.18 [-1.04, 0.76]	58	0.51
velocity range – variance (mm^2/s^2)	0.7108	0.41 ± 0.29	0.36 ± 0.26	0.18 [-0.77, 1.40]	58	0.56	0.4920	0.77 ± 0.49	0.69 ± 0.71	0.13 [-0.91, 1.05]	56	0.60	0.9298	0.55 ± 0.38	0.68 ± 0.61	-0.23 [-1.02, 0.74]	58	0.52
velocity range – skewness	0.2854	0.34 ± 0.21	0.26 ± 0.28	0.31 [-0.68, 1.59]	44	0.66	0.8286	0.34 ± 0.26	0.41 ± 0.37	-0.19 [-1.07, 0.82]	56	0.54	0.2109	0.33 ± 0.28	0.58 ± 0.43	-0.64 [-1.43, 0.39]	56	0.69
velocity range – kurtosis	0.9298	0.76 ± 0.52	0.94 ± 0.99	-0.20 [-1.05, 0.81]	50	0.52	0.3599	0.88 ± 0.73	1.66 ± 1.57	-0.59 [-1.44, 0.33]	33	0.64	0.2463	0.84 ± 0.76	2.12 ± 2.10	-0.71 [-1.39, 0.05]	33	0.68
velocity range – median (mm/s)	0.1198	0.49 ± 0.27	0.37 ± 0.48	0.28 [-0.75, 1.54]	58	0.73	0.1774	0.55 ± 0.37	0.31 ± 0.17	0.83 [-0.37, 1.88]	68	0.69	0.8601	0.50 ± 0.31	0.49 ± 0.31	0.00 [-1.12, 1.12]	58	0.53
velocity range – interquartile range (mm/s)	0.6504	0.37 ± 0.29	0.28 ± 0.24	0.32 [-0.68, 1.46]	58	0.57	0.4920	0.63 ± 0.33	0.63 ± 0.68	0.01 [-0.93, 1.03]	56	0.60	0.5962	0.53 ± 0.34	0.73 ± 0.53	-0.40 [-1.34, 0.47]	58	0.58
velocity range – range (mm/s)	0.9671	0.63 ± 0.70	0.66 ± 0.88	-0.04 [-0.82, 0.96]	58	0.51	0.8404	1.29 ± 1.44	1.35 ± 1.95	-0.03 [-0.91, 0.96]	58	0.53	0.4789	0.63 ± 0.74	1.00 ± 1.15	-0.35 [-1.01, 0.88]	58	0.61
fractal dimension	0.0556	0.31 ± 0.18	0.15 ± 0.11	1.09 [-0.18, 2.44]	58	0.77	0.0908	0.31 ± 0.20	0.22 ± 0.30	0.32 [-0.76, 1.55]	56	0.74	0.6590	0.27 ± 0.22	0.25 ± 0.23	0.09 [-0.75, 1.29]	56	0.57
fractal R^2	0.7732	$2.42.10^{-3} \pm 1.55.10^{-3}$	0.03 ± 0.08	-0.48 [-0.99, 0.16]	58	0.55	0.4920	$2.30.10^{-3} \pm 2.09.10^{-3}$	0.03 ± 0.09	-0.42 [-0.67, 0.91]	37	0.60	0.5360	$2.46.10^{-3} \pm 1.92.10^{-3}$	0.02 ± 0.03	-0.56 [-0.99, 0.48]	42	0.60

	Measurement 1 $\rightarrow$ 2						Measurement 2 $\rightarrow$ 3						Measurement 1 $\rightarrow$ 3					
**Feature**	**p**	**pCR**	**non-pCR**	**Effect size**	**Acc (%)**	**AUC**	**p**	**pCR**	**non-pCR**	**Effect size**	**Acc (%)**	**AUC**	**p**	**pCR**	**non-pCR**	**Effect size**	**Acc (%)**	**AUC**
minimum (F_{min})																		
compensated vessel coverage (%)	0.2030	16.87 ± 20.28	8.56 ± 10.65	0.53 [-0.44, 1.57]	50	0.68	0.9692	8.47 ± 12.53	8.57 ± 12.09	-0.01 [-0.80, 1.07]	60	0.51	0.2976	12.79 ± 14.61	7.46 ± 9.40	0.44 [-0.49, 1.49]	60	0.65
distances – mean (mm)	0.0078	0.14 ± 0.08	0.93 ± 0.84	-1.16 [-1.85, -0.67]	75	0.86	0.0972	0.31 ± 0.36	0.72 ± 0.70	-0.66 [-1.37, 0.29]	60	0.73	0.0409	0.28 ± 0.32	0.65 ± 0.63	-0.68 [-1.37, 0.11]	60	0.78
distances – variance (mm^2)	0.0228	0.06 ± 0.05	1.28 ± 2.02	-0.74 [-1.07, -0.38]	70	0.81	0.2030	0.24 ± 0.37	1.07 ± 1.68	-0.60 [-1.34, 0.10]	60	0.68	0.0491	0.13 ± 0.19	0.84 ± 1.37	-0.62 [-1.14, -0.12]	60	0.77
distances – skewness	0.6713	1.62 ± 1.33	1.08 ± 0.71	0.52 [-0.34, 1.89]	60	0.56	0.7285	0.99 ± 0.55	1.15 ± 0.81	-0.22 [-1.01, 0.76]	60	0.55	0.9079	1.15 ± 0.58	1.02 ± 0.65	0.21 [-0.66, 1.14]	60	0.52
distances – kurtosis	0.4636	3.84 ± 7.99	1.09 ± 1.98	0.50 [-0.41, 2.05]	60	0.60	0.7285	0.66 ± 1.74	1.66 ± 3.49	-0.33 [-0.92, 0.72]	60	0.55	0.7285	0.92 ± 1.85	0.89 ± 1.88	0.02 [-0.90, 1.11]	60	0.55
distances – median (mm)	0.0038	0.07 ± 0.04	0.64 ± 0.66	-1.06 [-1.58, -0.53]	70	0.90	0.0639	0.18 ± 0.24	0.45 ± 0.43	-0.70 [-1.36, 0.26]	60	0.76	0.0205	0.20 ± 0.29	0.40 ± 0.40	-0.52 [-1.27, 0.41]	60	0.82
distances – interquartile range (mm)	0.0062	0.14 ± 0.09	1.12 ± 1.12	-1.07 [-1.71, -0.63]	70	0.88	0.0697	0.33 ± 0.40	0.85 ± 0.90	-0.68 [-1.26, 0.22]	60	0.75	0.0408	0.31 ± 0.37	0.79 ± 0.86	-0.65 [-1.22, 0.30]	60	0.78
distances – range (mm)	0.0491	1.57 ± 0.89	4.14 ± 2.86	-1.07 [-1.88, -0.31]	45	0.77	0.3749	2.30 ± 1.75	3.89 ± 3.44	-0.53 [-1.18, 0.31]	45	0.63	0.0228	1.55 ± 1.06	3.47 ± 2.57	-0.87 [-1.57, -0.23]	55	0.81
flow direction – variance (rad^2)	0.7871	3.27 ± 0.22	3.17 ± 0.51	0.23 [-0.74, 0.97]	60	0.54	0.4179	3.22 ± 0.25	3.23 ± 0.49	-0.03 [-1.04, 0.81]	60	0.61	0.9692	3.29 ± 0.23	3.30 ± 0.26	-0.05 [-0.99, 0.97]	60	0.51
track length – mean (mm)	0.4179	0.59 ± 0.10	0.49 ± 0.16	0.67 [-0.06, 1.38]	60	0.61	0.5628	0.53 ± 0.08	0.48 ± 0.15	0.36 [-0.56, 1.16]	60	0.58	0.4636	0.55 ± 0.12	0.47 ± 0.18	0.49 [-0.41, 1.28]	60	0.60
track length – variance (mm^2)	0.2318	0.25 ± 0.08	0.19 ± 0.11	0.60 [-0.26, 1.49]	60	0.67	0.5120	0.21 ± 0.07	0.19 ± 0.13	0.22 [-0.57, 1.07]	60	0.59	0.4179	0.23 ± 0.09	0.18 ± 0.12	0.40 [-0.51, 1.32]	60	0.61
track length – skewness	0.8471	1.84 ± 0.25	1.77 ± 0.43	0.20 [-0.67, 1.12]	60	0.53	0.7285	1.95 ± 0.38	1.89 ± 0.39	0.16 [-0.83, 1.01]	60	0.55	0.9692	1.83 ± 0.34	1.91 ± 0.44	-0.19 [-1.03, 0.70]	60	0.51
track length – kurtosis	0.6160	4.98 ± 1.73	4.22 ± 2.50	0.32 [-0.51, 1.16]	55	0.57	0.7285	5.34 ± 2.33	4.88 ± 2.57	0.18 [-0.73, 1.08]	60	0.55	0.9692	4.78 ± 1.95	5.44 ± 3.23	-0.23 [-1.02, 0.59]	60	0.51
track length – median (mm)	0.1137	0.45 ± 0.09	0.35 ± 0.11	0.91 [0.11, 1.67]	60	0.72	0.2318	0.41 ± 0.07	0.35 ± 0.12	0.53 [-0.30, 1.37]	60	0.67	0.2633	0.41 ± 0.10	0.33 ± 0.13	0.67 [-0.17, 1.57]	60	0.66

Feature	Measurement 1 → 2						Measurement 2 → 3						Measurement 1 → 3					
	p	pCR	non-pCR	Effect size	Acc (%)	AUC	p	pCR	non-pCR	Effect size	Acc (%)	AUC	p	pCR	non-pCR	Effect size	Acc (%)	AUC
track length – interquartile range (mm)	0.3749	0.55 ± 0.07	0.44 ± 0.20	0.65 [-0.05, 1.28]	60	0.63	0.7285	0.48 ± 0.08	0.42 ± 0.19	0.36 [-0.50, 1.15]	60	0.55	0.3348	0.50 ± 0.11	0.42 ± 0.17	0.52 [-0.29, 1.51]	60	0.64
track length – range (mm)	0.2318	3.43 ± 0.92	2.69 ± 1.43	0.56 [-0.30, 1.36]	60	0.67	0.7285	2.94 ± 0.93	2.72 ± 1.59	0.16 [-0.70, 1.01]	60	0.55	0.7871	3.15 ± 1.29	2.94 ± 1.68	0.14 [-0.78, 1.03]	60	0.54
tortuosity (DM) – mean	0.0409	2.01 ± 0.15	2.41 ± 0.84	-0.58 [-1.04, 0.25]	55	0.78	0.1325	2.04 ± 0.16	2.18 ± 0.29	-0.57 [-1.49, 0.39]	60	0.71	0.9692	2.22 ± 0.26	2.18 ± 0.21	0.18 [-0.68, 1.25]	60	0.51
tortuosity (DM) – variance	0.7871	5.73 ± 2.94	6.30 ± 4.14	-0.15 [-0.93, 0.85]	55	0.54	0.9692	7.84 ± 8.13	5.51 ± 3.33	0.39 [-0.53, 2.01]	60	0.51	0.7871	8.20 ± 5.95	6.79 ± 2.82	0.31 [-0.70, 1.54]	60	0.54
tortuosity (DM) – skewness	0.4636	5.36 ± 2.48	4.62 ± 2.34	0.29 [-0.54, 1.38]	60	0.60	0.6713	4.38 ± 1.72	4.51 ± 2.92	-0.05 [-0.90, 0.83]	55	0.56	0.5628	5.91 ± 4.07	4.34 ± 1.49	0.54 [-0.64, 1.77]	60	0.58
tortuosity (DM) – kurtosis	0.6160	47.06 ± 53.38	35.81 ± 36.14	0.25 [-0.60, 1.51]	60	0.57	0.7871	27.10 ± 22.22	41.69 ± 74.91	-0.23 [-0.91, 0.80]	60	0.54	0.8471	66.95 ± 90.48	28.15 ± 20.01	0.63 [-0.53, 1.84]	60	0.53
tortuosity (DM) – median	0.0186	1.26 ± 0.04	1.45 ± 0.37	-0.61 [-0.86, -0.25]	70	0.82	0.0228	1.27 ± 0.04	1.33 ± 0.08	-0.94 [-1.80, 0.11]	60	0.81	0.6713	1.36 ± 0.12	1.32 ± 0.08	0.39 [-0.60, 1.32]	60	0.56
tortuosity (DM) – interquartile range	0.0228	0.87 ± 0.10	1.10 ± 0.46	-0.60 [-1.57, 0.32]	50	0.81	0.0279	0.86 ± 0.10	1.03 ± 0.39	-0.51 [-1.82, 0.54]	60	0.80	0.5628	1.05 ± 0.16	1.07 ± 0.19	-0.12 [-1.06, 0.83]	60	0.58
tortuosity (DM) – range	1.0000	28.51 ± 21.81	24.59 ± 17.19	0.20 [-0.65, 1.30]	60	0.50	0.8471	21.81 ± 9.92	23.16 ± 20.31	-0.08 [-0.90, 0.87]	60	0.53	0.4636	36.44 ± 24.91	25.18 ± 12.75	0.58 [-0.51, 1.95]	60	0.60
tortuosity (SOAM) – mean (rad/mm)	0.0018	6.07± 0.40	7.93 ± 1.85	-1.21 [-1.99, -0.29]	90	0.93	0.0097	6.44 ± 0.81	7.77 ± 0.95	-1.42 [-2.52, -0.32]	70	0.85	0.4636	6.94 ± 0.82	7.36 ± 0.86	-0.47 [-1.46, 0.48]	60	0.60
tortuosity (SOAM) – variance (rad^2/mm^2)	0.1137	43.94 ± 3.60	52.25 ± 26.81	-0.38 [-1.11, 0.62]	60	0.72	0.2976	47.38 ± 14.02	55.20 ± 24.00	-0.36 [-1.38, 0.61]	60	0.65	0.0972	47.11 ± 12.93	56.66 ± 9.79	-0.82 [-1.84, 0.07]	60	0.73
tortuosity (SOAM) – skewness	0.1325	1.69 ± 0.42	1.25 ± 0.63	0.76 [-0.09, 1.44]	45	0.71	0.7285	1.35 ± 0.31	1.29 ± 0.47	0.14 [-0.70, 1.00]	60	0.55	0.4179	1.55 ± 0.46	1.25 ± 0.61	0.52 [-0.41, 1.27]	60	0.61
tortuosity (SOAM) – kurtosis	0.2318	3.57 ± 2.35	2.02 ± 1.85	0.72 [-0.11, 1.68]	60	0.67	0.9079	1.83 ± 1.12	1.84 ± 1.95	-0.01 [-0.80, 0.86]	60	0.52	0.5628	2.86 ± 2.27	1.93 ± 1.94	0.43 [-0.44, 1.44]	60	0.58
tortuosity (SOAM) –	0.0049	3.92 ± 0.56	5.80 ± 2.09	-1.08 [-1.60, -0.33]	75	0.89	0.0049	4.07 ± 0.66	5.29 ± 0.82	-1.53 [-2.32, -0.59]	75	0.89	0.8471	4.86 ± 0.99	4.88 ± 0.77	-0.02 [-0.93, 1.00]	60	0.53

	Measurement 1 → 2						Measurement 2 → 3						Measurement 1 → 3					
**Feature**	**p**	**pCR**	**non-pCR**	**Effect size**	**Acc (%)**	**AUC**	**p**	**pCR**	**non-pCR**	**Effect size**	**Acc (%)**	**AUC**	**p**	**pCR**	**non-pCR**	**Effect size**	**Acc (%)**	**AUC**
median (rad/mm)																		
tortuosity (SOAM) – interquartile range (rad/mm)	0.0122	6.51 ± 0.33	8.06 ± 3.03	-0.62 [-2.20, 0.54]	60	0.84	0.0587	7.04 ± 1.29	8.22 ± 2.97	-0.46 [-1.78, 0.53]	60	0.76	0.1535	7.33 ± 1.35	8.25 ± 1.27	-0.69 [-1.71, 0.28]	60	0.70
tortuosity (SOAM) – range (rad/mm)	0.7285	44.30 ± 7.60	39.57 ± 16.01	0.34 [-0.54, 1.06]	60	0.55	0.6160	39.14 ± 10.78	40.48 ± 17.22	-0.09 [-0.93, 0.83]	60	0.57	0.6713	43.95 ± 11.88	43.77 ± 7.00	0.02 [-1.32, 0.97]	60	0.56
velocity – mean (mm/s)	0.1770	2.29 ± 0.23	2.01 ± 0.40	0.76 [-0.20, 1.45]	60	0.69	0.9692	2.04 ± 0.29	2.04 ± 0.29	0.00 [-1.03, 0.95]	60	0.51	0.7285	2.10 ± 0.32	2.00 ± 0.38	0.27 [-0.62, 1.16]	60	0.55
velocity – variance (mm^2/s^2)	0.4179	2.31 ± 0.19	2.04 ± 0.69	0.47 [-0.32, 1.04]	60	0.61	0.7871	2.10 ± 0.44	1.96 ± 0.73	0.22 [-0.68, 0.98]	60	0.54	0.3749	2.06 ± 0.41	2.11 ± 0.61	-0.09 [-1.19, 0.74]	60	0.63
velocity – skewness	0.1535	0.63 ± 0.14	0.70 ± 0.32	-0.27 [-1.41, 0.67]	55	0.70	0.1137	0.63 ± 0.15	0.74 ± 0.35	-0.34 [-1.30, 0.75]	60	0.72	0.6160	0.72 ± 0.15	0.71 ± 0.14	0.08 [-0.85, 1.10]	60	0.57
velocity – kurtosis	0.7285	-0.36 ± 0.26	-0.33 ± 0.59	-0.05 [-0.91, 0.79]	60	0.55	0.5628	-0.37 ± 0.22	-0.20 ± 0.71	-0.29 [-1.09, 0.64]	60	0.58	0.1325	-0.20 ± 0.29	-0.30 ± 0.37	0.29 [-0.49, 1.32]	60	0.71
velocity – median (mm/s)	0.2633	1.90 ± 0.32	1.62 ± 0.41	0.72 [-0.06, 1.51]	60	0.66	0.9692	1.64 ± 0.29	1.65 ± 0.37	-0.03 [-0.88, 0.91]	60	0.51	0.4636	1.75 ± 0.36	1.57 ± 0.34	0.48 [-0.41, 1.47]	60	0.60
velocity – interquartile range (mm/s)	0.1137	2.18 ± 0.16	1.85 ± 0.57	0.70 [-0.14, 1.23]	60	0.72	0.7871	1.97 ± 0.30	1.82 ± 0.56	0.29 [-0.65, 1.05]	60	0.54	0.9692	1.99 ± 0.31	1.93 ± 0.46	0.15 [-0.85, 0.81]	60	0.51
velocity – range (mm/s)	1.0000	6.73 ± 0.29	6.02 ± 1.92	0.45 [-0.45, 0.91]	60	0.50	0.7285	6.23 ± 0.90	5.97 ± 1.90	0.16 [-0.87, 0.84]	60	0.55	0.5120	6.37 ± 0.94	6.47 ± 0.83	-0.11 [-1.23, 0.78]	60	0.59
velocity range – mean (mm/s)	0.2633	2.04 ± 0.34	1.73 ± 0.42	0.77 [0.08, 1.62]	60	0.66	0.7285	1.83 ± 0.25	1.74 ± 0.39	0.24 [-0.59, 1.06]	60	0.55	0.4179	1.88 ± 0.32	1.66 ± 0.42	0.55 [-0.24, 1.39]	60	0.61
velocity range – variance (mm^2/s^2)	0.2633	1.95 ± 0.42	1.56 ± 0.70	0.60 [-0.21, 1.33]	60	0.66	0.7285	1.58 ± 0.48	1.41 ± 0.69	0.27 [-0.76, 1.05]	60	0.55	0.6713	1.65 ± 0.53	1.50 ± 0.60	0.25 [-0.82, 1.04]	60	0.56
velocity range – skewness	0.0014	0.66 ± 0.11	1.14 ± 0.51	-1.13 [-1.67, -0.70]	85	0.94	0.0339	0.73 ± 0.25	0.97 ± 0.21	-1.04 [-1.84, 0.06]	80	0.79	0.0826	0.66 ± 0.21	0.84 ± 0.13	-1.00 [-2.00, 0.07]	75	0.74
velocity range – kurtosis	0.0023	-0.29 ± 0.24	1.23 ± 2.22	-0.84 [-1.19, -0.45]	80	0.92	0.0339	-0.24 ± 0.63	0.32 ± 0.63	-0.85 [-1.71, 0.22]	70	0.79	0.0698	-0.34 ± 0.45	-0.01 ± 0.37	-0.80 [-1.57, 0.22]	70	0.75

Feature	Measurement 1 → 2						Measurement 2 → 3						Measurement 1 → 3					
	p	pCR	non-pCR	Effect size	Acc (%)	AUC	p	pCR	non-pCR	Effect size	Acc (%)	AUC	p	pCR	non-pCR	Effect size	Acc (%)	AUC
velocity range – median (mm/s)	0.1535	1.70 ± 0.37	1.37 ± 0.35	0.88 [0.00, 1.71]	65	0.70	0.6160	1.46 ± 0.24	1.38 ± 0.36	0.22 [-0.74, 1.15]	60	0.57	0.2976	1.53 ± 0.33	1.32 ± 0.35	0.59 [-0.32, 1.47]	60	0.65
velocity range – interquartile range (mm/s)	0.2633	1.90 ± 0.37	1.51 ± 0.64	0.68 [-0.14, 1.41]	60	0.66	0.4179	1.61 ± 0.32	1.35 ± 0.59	0.50 [-0.35, 1.28]	60	0.61	0.2318	1.69 ± 0.39	1.39 ± 0.50	0.64 [-0.23, 1.51]	60	0.67
velocity range – range (mm/s)	0.6160	6.38 ± 0.66	5.76 ± 1.93	0.38 [-0.47, 0.98]	60	0.57	0.3749	5.51 ± 1.35	5.40 ± 2.09	0.06 [-0.86, 0.82]	60	0.63	0.7285	5.79 ± 1.42	5.77 ± 1.46	0.01 [-1.13, 0.76]	60	0.55
fractal dimension	0.4179	1.24 ± 0.18	1.12 ± 0.29	0.46 [-0.52, 1.16]	55	0.61	0.7285	1.11 ± 0.18	1.11 ± 0.31	0.02 [-0.97, 0.83]	60	0.55	0.5628	1.19 ± 0.20	1.13 ± 0.25	0.27 [-0.59, 1.07]	60	0.58
fractal R^2	0.6713	0.99 ± $2.29.10^{-3}$	0.96 ± 0.09	0.49 [-0.28, 0.99]	60	0.56	0.7285	0.99 ± $3.59.10^{-3}$	0.97 ± 0.08	0.41 [-1.07, 0.71]	60	0.55	0.5628	0.99 ± $4.20.10^{-3}$	0.98 ± 0.04	0.47 [-0.50, 0.88]	55	0.58
maximum (F_{max})																		
compensated vessel coverage (%)	0.0023	41.71 ± 11.45	16.06 ± 14.57	1.83 [0.71, 3.42]	80	0.92	0.0339	34.14 ± 17.61	17.28 ± 13.09	1.08 [-0.09, 2.36]	70	0.79	0.0587	31.39 ± 16.33	17.99 ± 12.82	0.90 [-0.28, 2.14]	70	0.76
distances – mean (mm)	0.9079	1.04 ± 0.55	1.67 ± 1.53	-0.48 [-1.24, 0.20]	60	0.55	0.5120	1.33 ± 1.10	1.73 ± 1.44	-0.29 [-1.06, 0.80]	60	0.56	0.4402	1.08 ± 1.21	1.62 ± 0.97	-0.48 [-1.46, 0.91]	60	0.66
distances – variance (mm^2)	0.4636	0.80 ± 0.57	2.51 ± 2.96	-0.70 [-1.38, -0.02]	60	0.60	0.9692	1.79 ± 2.92	2.42 ± 2.85	-0.21 [-1.00, 0.94]	60	0.51	0.3749	1.55 ± 2.99	2.06 ± 2.54	-0.18 [-1.01, 0.97]	60	0.63
distances – skewness	0.0151	2.86 ± 0.95	1.66 ± 1.03	1.15 [0.10, 2.17]	65	0.83	0.0826	2.58 ± 1.08	1.74 ± 0.69	0.93 [-0.07, 1.93]	60	0.74	0.5628	2.01 ± 0.80	1.96 ± 0.92	0.06 [-0.92, 1.04]	60	0.58
distances – kurtosis	0.0186	10.55 ± 6.30	4.22 ± 6.01	0.99 [-0.07, 2.04]	65	0.82	0.1137	8.71 ± 7.32	3.90 ± 4.35	0.81 [-0.22, 1.73]	65	0.72	0.5628	5.33 ± 3.71	5.41 ± 6.19	-0.02 [-0.84, 0.88]	65	0.58
distances – median (mm)	0.9079	0.80 ± 0.57	1.35 ± 1.50	-0.42 [-1.10, 0.39]	60	0.55	0.6160	1.09 ± 0.91	1.34 ± 1.42	-0.20 [-0.99, 0.76]	60	0.53	0.3157	0.78 ± 0.98	1.29 ± 0.92	-0.51 [-1.47, 1.21]	61	0.70
distances – interquartile range (mm)	0.4179	1.06 ± 0.68	1.70 ± 1.34	-0.54 [-1.20, 0.39]	60	0.61	0.9079	1.62 ± 1.55	1.68 ± 1.37	-0.04 [-0.85, 1.25]	60	0.52	0.4179	1.40 ± 1.59	1.62 ± 1.13	-0.15 [-1.13, 1.42]	60	0.61
distances – range (mm)	0.3749	3.98 ± 1.55	5.92 ± 4.16	-0.55 [-1.26, 0.30]	60	0.63	0.7285	4.64 ± 2.99	5.69 ± 3.79	-0.29 [-1.07, 0.77]	60	0.55	0.1325	4.02 ± 3.12	5.78 ± 3.72	-0.48 [-1.26, 0.62]	60	0.71

	Measurement 1 → 2						Measurement 2 → 3						Measurement 1 → 3					
**Feature**	**p**	**pCR**	**non-pCR**	**Effect size**	**Acc (%)**	**AUC**	**p**	**pCR**	**non-pCR**	**Effect size**	**Acc (%)**	**AUC**	**p**	**pCR**	**non-pCR**	**Effect size**	**Acc (%)**	**AUC**
flow direction – variance (rad^2)	0.8471	3.52 ± 0.26	3.55 ± 0.26	-0.11 [-1.07, 0.86]	60	0.53	0.9692	3.59 ± 0.29	3.73 ± 0.50	-0.31 [-1.00, 0.73]	60	0.51	0.6160	3.55 ± 0.32	3.75 ± 0.52	-0.43 [-1.19, 0.44]	60	0.57
track length – mean (mm)	0.0023	0.71 ± 0.06	0.57 ± 0.12	1.42 [0.82, 2.05]	75	0.92	0.0122	0.69 ± 0.07	0.59 ± 0.09	1.25 [0.27, 1.98]	70	0.84	0.1535	0.65 ± 0.13	0.59 ± 0.12	0.49 [-0.81, 1.39]	60	0.70
track length – variance (mm^2)	0.1137	0.35 ± 0.08	0.25 ± 0.13	0.87 [0.22, 1.68]	60	0.72	0.1770	0.36 ± 0.07	0.29 ± 0.11	0.63 [-0.27, 1.64]	60	0.69	0.5120	0.33 ± 0.13	0.30 ± 0.11	0.24 [-0.76, 1.31]	60	0.59
track length – skewness	0.2976	2.34 ± 0.28	2.19 ± 0.39	0.42 [-0.51, 1.20]	60	0.65	0.9079	2.35 ± 0.41	2.44 ± 0.59	-0.17 [-1.05, 0.71]	60	0.52	0.7871	2.27 ± 0.29	2.40 ± 0.63	-0.25 [-1.03, 0.56]	60	0.54
track length – kurtosis	0.2318	8.62 ± 2.18	7.26 ± 3.04	0.48 [-0.28, 1.57]	45	0.67	0.9692	8.30 ± 2.80	9.22 ± 5.40	-0.19 [-0.96, 0.82]	60	0.51	0.6160	7.37 ± 1.92	8.97 ± 5.54	-0.34 [-1.01, 0.54]	60	0.57
track length – median (mm)	0.0018	0.57 ± 0.04	0.44 ± 0.09	1.49 [0.81, 2.19]	80	0.93	0.0049	0.54 ± 0.06	0.43 ± 0.07	1.54 [0.54, 2.47]	80	0.89	0.1770	0.52 ± 0.09	0.45 ± 0.11	0.58 [-0.47, 1.34]	65	0.69
track length – interquartile range (mm)	0.0186	0.63 ± 0.04	0.52 ± 0.15	0.87 [0.48, 1.45]	60	0.82	0.0186	0.64 ± 0.04	0.55 ± 0.09	1.14 [0.38, 1.82]	70	0.82	0.2318	0.60 ± 0.11	0.55 ± 0.12	0.37 [-0.98, 1.07]	60	0.67
track length – range (mm)	0.0122	5.12 ± 0.90	3.60 ± 1.57	1.08 [0.15, 1.90]	65	0.84	0.3749	4.60 ± 1.12	4.07 ± 1.49	0.37 [-0.52, 1.34]	60	0.63	0.4179	4.63 ± 1.63	4.23 ± 1.54	0.24 [-0.92, 1.11]	60	0.61
tortuosity (DM) – mean	0.0826	2.47 ± 0.60	3.01 ± 1.23	-0.50 [-1.05, 0.85]	45	0.74	0.7871	2.79 ± 0.66	2.85 ± 1.24	-0.06 [-0.96, 1.05]	60	0.54	0.7871	2.61 ± 0.48	2.78 ± 0.79	-0.24 [-0.97, 0.69]	60	0.54
tortuosity (DM) – variance	0.7871	16.22 ± 9.53	17.29 ± 10.01	-0.10 [-1.04, 0.98]	60	0.54	0.7285	20.16 ± 17.67	14.07 ± 10.32	0.43 [-0.60, 1.48]	60	0.55	0.1535	23.35 ± 17.18	13.51 ± 7.59	0.77 [-0.25, 1.88]	60	0.70
tortuosity (DM) – skewness	0.0826	12.43 ± 5.33	8.51 ± 5.56	0.69 [-0.35, 1.64]	50	0.74	0.4636	7.95 ± 3.64	7.82 ± 5.66	0.02 [-0.82, 1.09]	60	0.60	0.1137	11.57 ± 6.50	7.72 ± 3.18	0.77 [-0.59, 1.71]	65	0.72
tortuosity (DM) – kurtosis	0.0409	265.55 ± 236.61	134.35 ± 164.61	0.64 [-0.32, 1.58]	60	0.78	0.4179	100.86 ± 84.28	117.44 ± 168.17	-0.11 [-0.84, 0.90]	75	0.61	0.0972	247.34 ± 250.85	97.80 ± 80.11	0.85 [-0.20, 1.74]	70	0.73
tortuosity (DM) – median	0.0409	1.40 ± 0.15	1.98 ± 1.52	-0.47 [-0.76, 0.52]	45	0.78	0.9079	1.51 ± 0.20	1.90 ± 1.52	-0.31 [-1.09, 0.75]	60	0.52	0.2030	1.46 ± 0.18	1.68 ± 0.41	-0.60 [-1.26, 0.15]	60	0.68
tortuosity (DM) – interquartile range	0.0409	1.27 ± 0.47	1.87 ± 1.08	-0.64 [-1.20, 0.42]	50	0.78	0.8471	1.52 ± 0.57	1.47 ± 0.35	0.10 [-0.91, 1.29]	60	0.53	0.1535	1.37 ± 0.47	1.89 ± 1.08	-0.56 [-1.08, 0.38]	60	0.70

Feature	Measurement 1 → 2						Measurement 2 → 3						Measurement 1 → 3					
	p	pCR	non-pCR	Effect size	Acc (%)	AUC	p	pCR	non-pCR	Effect size	Acc (%)	AUC	p	pCR	non-pCR	Effect size	Acc (%)	AUC
tortuosity (DM) – range	0.1535	83.82 ± 41.03	59.11 ± 42.65	0.56 [-0.46, 1.70]	55	0.70	0.4179	56.03 ± 32.25	51.21 ± 46.90	0.11 [-0.75, 1.02]	80	0.61	0.1137	82.96 ± 49.46	46.41 ± 19.26	1.02 [-0.64, 2.12]	80	0.72
tortuosity (SOAM) – mean (rad/mm)	0.2030	8.11 ± 1.51	10.35 ± 3.41	-0.76 [-1.39, 0.03]	60	0.68	0.7871	9.01 ± 1.48	9.46 ± 1.83	-0.25 [-1.04, 0.73]	60	0.54	0.1535	8.52 ± 1.61	10.65 ± 3.36	-0.73 [-1.36, 0.11]	60	0.70
tortuosity (SOAM) – variance (rad^2/mm^2)	0.0097	63.61 ± 13.54	88.01 ± 20.01	-1.31 [-2.02, -0.60]	65	0.85	0.4636	72.44 ± 20.29	80.30 ± 19.47	-0.38 [-1.25, 0.72]	60	0.60	0.2030	68.35 ± 26.77	87.69 ± 25.59	-0.71 [-1.67, 0.23]	60	0.68
tortuosity (SOAM) – skewness	0.0491	2.20 ± 0.31	1.64 ± 0.77	0.85 [-0.18, 1.54]	75	0.77	0.0279	2.14 ± 0.33	1.74 ± 0.34	1.13 [0.09, 2.17]	70	0.80	0.8471	1.84 ± 0.49	1.89 ± 0.32	-0.11 [-1.13, 1.01]	70	0.53
tortuosity (SOAM) – kurtosis	0.0698	6.68 ± 1.96	4.31 ± 3.28	0.80 [-0.12, 1.95]	65	0.75	0.0279	6.01 ± 2.32	3.83 ± 2.07	0.96 [-0.32, 2.12]	75	0.80	0.9079	4.65 ± 2.41	4.89 ± 2.00	-0.11 [-1.04, 0.97]	75	0.52
tortuosity (SOAM) – median (rad/mm)	0.1770	5.83 ± 1.85	8.48 ± 4.71	-0.66 [-1.16, 0.13]	60	0.69	0.2976	6.62 ± 1.69	7.28 ± 1.89	-0.35 [-1.27, 0.78]	60	0.65	0.1770	5.96 ± 1.29	8.62 ± 4.63	-0.69 [-1.27, -0.05]	60	0.69
tortuosity (SOAM) – interquartile range (rad/mm)	0.0826	9.12 ± 1.92	11.24 ± 2.45	-0.90 [-1.77, -0.07]	60	0.74	0.2976	9.77 ± 2.05	11.23 ± 2.76	-0.56 [-1.32, 0.27]	60	0.65	0.0698	9.29 ± 2.42	11.95 ± 3.10	-0.89 [-1.72, -0.15]	65	0.75
tortuosity (SOAM) – range (rad/mm)	0.1770	51.87 ± 3.31	55.39 ± 8.20	-0.50 [-1.34, 0.48]	60	0.69	0.9079	49.94 ± 5.59	51.12 ± 10.79	-0.12 [-0.91, 0.70]	60	0.52	0.6160	47.73 ± 12.35	52.92 ± 4.84	-0.58 [-1.86, 0.17]	60	0.57
velocity – mean (mm/s)	0.0491	2.56 ± 0.19	2.37 ± 0.18	0.96 [0.13, 1.91]	70	0.77	0.0491	2.55 ± 0.21	2.30 ± 0.28	0.96 [0.11, 1.80]	60	0.77	0.7871	2.40 ± 0.32	2.44 ± 0.19	-0.12 [-1.39, 0.79]	60	0.54
velocity – variance (mm^2/s^2)	0.4636	2.51 ± 0.26	2.49 ± 0.64	0.04 [-0.95, 0.86]	60	0.60	0.9692	2.49 ± 0.26	2.46 ± 0.28	0.10 [-0.83, 1.02]	60	0.51	0.0698	2.28 ± 0.45	2.63 ± 0.32	-0.90 [-1.79, -0.10]	60	0.75
velocity – skewness	0.1137	0.83 ± 0.18	1.11 ± 0.39	-0.81 [-1.39, -0.10]	45	0.72	0.8471	0.96 ± 0.13	1.01 ± 0.27	-0.22 [-1.05, 0.66]	60	0.53	0.1770	0.89 ± 0.17	1.11 ± 0.37	-0.69 [-1.28, 0.13]	60	0.69
velocity – kurtosis	0.0491	-0.04 ± 0.30	0.77 ± 1.52	-0.64 [-1.10, 0.18]	65	0.77	0.7285	0.21 ± 0.34	0.39 ± 0.65	-0.32 [-1.11, 0.56]	60	0.55	0.4179	0.13 ± 0.32	0.74 ± 1.54	-0.48 [-0.96, 0.54]	60	0.61
velocity – median (mm/s)	0.0409	2.28 ± 0.23	2.02 ± 0.22	1.13 [0.24, 2.01]	65	0.78	0.0228	2.27 ± 0.29	1.93 ± 0.32	1.08 [-0.05, 1.94]	65	0.81	0.5120	2.12 ± 0.34	2.08 ± 0.23	0.15 [-1.05, 1.12]	60	0.59

Feature	Measurement 1 → 2						Measurement 2 → 3						Measurement 1 → 3					
	p	pCR	non-pCR	Effect size	Acc (%)	AUC	p	pCR	non-pCR	Effect size	Acc (%)	AUC	p	pCR	non-pCR	Effect size	Acc (%)	AUC
velocity – interquartile range (mm/s)	0.8471	2.32 ± 0.20	2.20 ± 0.59	0.24 [-0.68, 0.97]	60	0.53	0.9079	2.31 ± 0.19	2.19 ± 0.30	0.42 [-0.35, 1.23]	60	0.52	0.1770	2.16 ± 0.34	2.36 ± 0.36	-0.53 [-1.42, 0.38]	60	0.69
velocity – range (mm/s)	0.2976	7.00 ± 0.08	6.65 ± 0.83	0.51 [-0.24, 0.94]	60	0.65	0.5628	6.96 ± 0.08	6.78 ± 0.45	0.48 [-0.48, 0.98]	60	0.58	0.6713	6.73 ± 0.71	6.88 ± 0.34	-0.27 [-1.37, 0.58]	60	0.56
velocity range – mean (mm/s)	0.0186	2.41 ± 0.16	2.08 ± 0.37	1.05 [0.38, 1.69]	75	0.82	0.0228	2.33 ± 0.24	2.02 ± 0.25	1.19 [0.04, 2.28]	80	0.81	0.2030	2.27 ± 0.29	2.12 ± 0.31	0.45 [-0.80, 1.25]	60	0.68
velocity range – variance (mm^2/s^2)	0.3348	2.30 ± 0.22	1.92 ± 0.66	0.68 [-0.20, 1.31]	60	0.64	0.0279	2.36 ± 0.28	2.04 ± 0.33	0.97 [-0.19, 1.82]	75	0.80	0.5628	2.13 ± 0.59	2.13 ± 0.45	0.01 [-1.27, 0.83]	60	0.58
velocity range – skewness	0.0698	0.96 ± 0.23	1.37 ± 0.48	-0.98 [-1.65, -0.29]	55	0.75	0.1535	1.06 ± 0.30	1.31 ± 0.39	-0.65 [-1.48, 0.22]	70	0.70	0.0491	0.95 ± 0.40	1.37 ± 0.41	-0.99 [-1.65, 0.00]	70	0.77
velocity range – kurtosis	0.0587	0.38 ± 0.61	2.09 ± 2.25	-0.91 [-1.49, -0.35]	45	0.76	0.1325	0.64 ± 0.80	1.70 ± 1.60	-0.76 [-1.53, 0.03]	65	0.71	0.0491	0.39 ± 1.05	1.94 ± 1.97	-0.89 [-1.44, -0.14]	60	0.77
velocity range – median (mm/s)	0.0228	2.12 ± 0.21	1.74 ± 0.41	1.06 [0.19, 1.76]	80	0.81	0.0587	2.00 ± 0.28	1.67 ± 0.34	1.01 [-0.18, 2.11]	80	0.76	0.0826	1.96 ± 0.34	1.77 ± 0.28	0.60 [-0.62, 1.62]	65	0.74
velocity range – interquartile range (mm/s)	0.0491	2.23 ± 0.17	1.80 ± 0.59	0.87 [0.19, 1.34]	70	0.77	0.0078	2.24 ± 0.29	1.92 ± 0.28	1.09 [-0.34, 2.09]	80	0.86	0.0826	2.16 ± 0.32	2.05 ± 0.26	0.36 [-1.10, 1.33]	60	0.74
velocity range – range (mm/s)	0.4179	6.94 ± 0.16	6.42 ± 1.13	0.55 [-0.23, 1.01]	60	0.61	0.6160	6.81 ± 0.23	6.64 ± 0.50	0.39 [-0.43, 1.04]	60	0.57	0.8471	6.35 ± 1.50	6.69 ± 0.67	-0.31 [-1.29, 0.57]	60	0.53
fractal dimension	0.0078	1.51 ± 0.08	1.27 ± 0.25	1.15 [0.54, 1.75]	65	0.86	0.1137	1.42 ± 0.15	1.31 ± 0.17	0.67 [-0.31, 1.50]	55	0.72	0.1325	1.43 ± 0.22	1.35 ± 0.18	0.36 [-1.10, 1.22]	55	0.71
fractal R^2	0.2030	1.00 ± 0.00	0.99 ± 0.01	0.45 [-0.62, 0.97]	60	0.68	1.0000	1.00 ± 0.00	0.99 ± 0.01	0.21 [-0.75, 1.02]	60	0.50	0.2633	1.00 ± 0.00	0.99 ± 0.01	0.19 [-0.85, 1.33]	60	0.66

Supplementary Table 10: Summary statistics of ULM-based radiomic features for the three measurements combined. Delta radiomic features include the minimum and maximum of the three measurements. From left to right: feature name, p-value for a statistical significance between patients with pCR and non-pCR, mean ± standard deviation of patients with pCR, mean ± standard deviation of patients with non-pCR, effect size expressed by Hedges' g with bootstrapped 95% confidence interval, cross-validated predictive accuracy, area under the receiver operating characteristic curve (AUC).

Feature	p	pCR	non-pCR	Effect size	Acc (%)	AUC
minimum ($F_{min}^{(1,2,3)}$)						
compensated vessel coverage (%)	0.6160	46.73 ± 35.58	59.80 ± 83.12	-0.18 [-0.98, 0.71]	60	0.57
distances – mean (mm)	0.0097	0.12 ± 0.07	0.62 ± 0.62	-0.97 [-1.61, -0.52]	70	0.85
distances – variance (mm^2)	0.0409	0.05 ± 0.05	0.80 ± 1.39	-0.67 [-1.19, -0.37]	60	0.78
distances – skewness	0.4636	0.82 ± 0.34	0.94 ± 0.70	-0.20 [-1.13, 0.67]	60	0.60
distances – kurtosis	0.6160	-0.06 ± 0.77	0.66 ± 1.84	-0.45 [-1.13, 0.46]	60	0.57
distances – median (mm)	0.0038	0.05 ± 0.03	0.38 ± 0.38	-1.05 [-1.87, -0.62]	75	0.90
distances – interquartile range (mm)	0.0048	0.12 ± 0.06	0.76 ± 0.87	-0.90 [-1.37, -0.49]	70	0.89
distances – range (mm)	0.0587	1.47 ± 0.93	3.32 ± 2.66	-0.82 [-1.48, -0.14]	40	0.76
flow direction – variance (rad^2)	0.9079	3.18 ± 0.20	3.14 ± 0.48	0.10 [-0.75, 0.91]	60	0.52
track length – mean (mm)	0.7285	0.51 ± 0.07	0.45 ± 0.17	0.41 [-0.39, 1.23]	60	0.55
track length – variance (mm^2)	0.4179	0.20 ± 0.06	0.16 ± 0.11	0.33 [-0.64, 1.27]	60	0.61
track length – skewness	0.9079	1.76 ± 0.31	1.72 ± 0.40	0.09 [-0.92, 0.94]	60	0.52
track length – kurtosis	0.6160	4.30 ± 1.82	3.95 ± 2.33	0.15 [-0.88, 1.05]	60	0.57
track length – median (mm)	0.2030	0.38 ± 0.06	0.32 ± 0.12	0.62 [-0.28, 1.49]	60	0.68
track length – interquartile range (mm)	0.7871	0.46 ± 0.06	0.40 ± 0.19	0.40 [-0.45, 1.16]	60	0.54
track length – range (mm)	0.6160	2.72 ± 0.76	2.45 ± 1.44	0.21 [-0.76, 1.04]	60	0.57
tortuosity (DM) – mean	0.1535	2.00 ± 0.13	2.11 ± 0.24	-0.50 [-1.48, 0.45]	50	0.70
tortuosity (DM) – variance	0.8471	4.79 ± 1.85	4.97 ± 3.04	-0.07 [-0.99, 0.77]	60	0.53
tortuosity (DM) – skewness	0.8471	3.97 ± 1.53	3.77 ± 1.39	0.14 [-0.83, 1.14]	60	0.53
tortuosity (DM) – kurtosis	0.9692	21.67 ± 18.85	20.71 ± 15.49	0.05 [-0.81, 1.25]	60	0.51
tortuosity (DM) – median	0.0826	1.26 ± 0.04	1.31 ± 0.07	-0.73 [-1.56, 0.19]	45	0.74
tortuosity (DM) – interquartile range	0.0972	0.86 ± 0.10	0.95 ± 0.35	-0.30 [-1.44, 0.76]	55	0.73
tortuosity (DM) – range	0.9692	19.43 ± 9.37	18.77 ± 12.48	0.06 [-0.89, 0.94]	60	0.51
tortuosity (SOAM) – mean (rad/mm)	0.0049	6.07 ± 0.40	7.22 ± 0.79	-1.64 [-2.75, -0.67]	75	0.89
tortuosity (SOAM) – variance (rad^2/mm^2)	0.1325	41.60 ± 8.62	45.81 ± 18.36	-0.26 [-1.28, 0.62]	60	0.71
tortuosity (SOAM) – skewness	0.4179	1.31 ± 0.24	1.09 ± 0.59	0.43 [-0.45, 1.18]	60	0.61
tortuosity (SOAM) – kurtosis	0.5628	1.64 ± 0.76	1.34 ± 1.75	0.20 [-0.69, 1.20]	60	0.58
tortuosity (SOAM) – median (rad/mm)	0.0049	3.88 ± 0.49	4.88 ± 0.77	-1.43 [-2.12, -0.55]	80	0.89
tortuosity (SOAM) – interquartile range (rad/mm)	0.0409	6.47 ± 0.42	7.36 ± 2.59	-0.42 [-1.94, 1.09]	60	0.78
tortuosity (SOAM) – range (rad/mm)	0.9079	39.14 ± 10.78	38.28 ± 15.58	0.06 [-0.85, 0.92]	60	0.52
velocity – mean (mm/s)	0.9692	1.99 ± 0.23	1.92 ± 0.37	0.23 [-0.70, 1.04]	60	0.51
velocity – variance (mm^2/s^2)	0.7871	2.05 ± 0.41	1.94 ± 0.72	0.18 [-0.76, 0.98]	60	0.54
velocity – skewness	0.2633	0.60 ± 0.11	0.64 ± 0.30	-0.14 [-1.20, 0.76]	55	0.66
velocity – kurtosis	0.7285	-0.41 ± 0.20	-0.44 ± 0.57	0.05 [-0.78, 0.93]	60	0.55
velocity – median (mm/s)	0.7871	1.58 ± 0.21	1.49 ± 0.35	0.29 [-0.61, 1.16]	60	0.54
velocity – interquartile range (mm/s)	0.6160	1.95 ± 0.29	1.80 ± 0.54	0.30 [-0.61, 1.02]	60	0.57
velocity – range (mm/s)	0.3348	6.20 ± 0.87	5.96 ± 1.90	0.15 [-0.81, 0.94]	60	0.64
velocity range – mean (mm/s)	0.5628	1.74 ± 0.15	1.61 ± 0.40	0.39 [-0.41, 1.27]	60	0.58
velocity range – variance (mm^2/s^2)	0.9079	1.49 ± 0.42	1.38 ± 0.64	0.20 [-0.69, 1.14]	60	0.52
velocity range – skewness	0.0049	0.62 ± 0.17	0.84 ± 0.13	-1.38 [-2.08, -0.32]	80	0.89
velocity range – kurtosis	0.0186	-0.40 ± 0.41	-0.01 ± 0.37	-0.99 [-1.79, -0.01]	70	0.82
velocity range – median (mm/s)	0.4179	1.38 ± 0.14	1.26 ± 0.33	0.42 [-0.49, 1.17]	60	0.61
velocity range – interquartile range (mm/s)	0.3749	1.55 ± 0.25	1.31 ± 0.57	0.48 [-0.33, 1.20]	60	0.63
velocity range – range (mm/s)	0.2976	5.46 ± 1.30	5.38 ± 2.07	0.05 [-0.99, 0.88]	60	0.65
fractal dimension	0.9079	1.09 ± 0.13	1.07 ± 0.29	0.07 [-0.77, 0.86]	60	0.52
fractal R^2	0.7871	0.99 ± 0.00	0.96 ± 0.09	0.50 [-0.08, 1.01]	60	0.54
maximum ($F_{max}^{(1,2,3)}$)						

Feature	p	pCR	non-pCR	Effect size	Acc (%)	AUC
compensated vessel coverage (%)	0.0030	42.56 ± 10.32	19.99 ± 13.90	1.71 [0.77, 2.76]	80	0.91
distances – mean (mm)	1.0000	1.54 ± 1.03	2.00 ± 1.44	-0.34 [-1.14, 0.73]	60	0.52
distances – variance (mm^2)	1.0000	1.92 ± 2.85	2.71 ± 2.99	-0.26 [-0.93, 0.99]	60	0.50
distances – skewness	0.0339	2.93 ± 0.86	2.01 ± 0.90	0.99 [-0.12, 1.79]	70	0.79
distances – kurtosis	0.0491	11.03 ± 5.71	5.74 ± 6.15	0.85 [-0.17, 1.90]	50	0.77
distances – median (mm)	0.8773	1.22 ± 0.89	1.65 ± 1.45	-0.33 [-1.06, 0.82]	60	0.56
distances – interquartile range (mm)	0.9079	1.83 ± 1.40	1.88 ± 1.31	-0.04 [-0.89, 1.17]	60	0.52
distances – range (mm)	0.4636	5.09 ± 2.57	6.28 ± 4.04	-0.32 [-1.16, 0.63]	60	0.60
flow direction – variance (rad^2)	0.5628	3.65 ± 0.26	3.82 ± 0.45	-0.43 [-1.11, 0.59]	60	0.58
track length – mean (mm)	0.0010	0.73 ± 0.05	0.60 ± 0.09	1.61 [0.89, 2.29]	70	0.95
track length – variance (mm^2)	0.0698	0.39 ± 0.07	0.31 ± 0.11	0.83 [-0.01, 1.71]	60	0.75
track length – skewness	0.8471	2.43 ± 0.32	2.48 ± 0.60	-0.10 [-0.97, 0.80]	60	0.53
track length – kurtosis	0.9692	9.01 ± 2.11	9.60 ± 5.37	-0.13 [-0.96, 0.68]	60	0.51
track length – median (mm)	0.0008	0.58 ± 0.03	0.47 ± 0.08	1.68 [0.91, 2.36]	75	0.96
track length – interquartile range (mm)	0.0097	0.65 ± 0.04	0.57 ± 0.09	1.04 [0.31, 1.64]	70	0.85
track length – range (mm)	0.0826	5.37 ± 0.69	4.28 ± 1.51	0.83 [-0.09, 1.65]	60	0.74
tortuosity (DM) – mean	0.8471	2.81 ± 0.63	3.04 ± 1.21	-0.21 [-1.01, 0.74]	60	0.53
tortuosity (DM) – variance	0.5120	24.19 ± 16.25	18.18 ± 9.90	0.45 [-0.59, 1.49]	50	0.59
tortuosity (DM) – skewness	0.1325	12.68 ± 5.22	9.20 ± 5.24	0.64 [-0.33, 1.58]	50	0.71
tortuosity (DM) – kurtosis	0.0491	268.63 ± 233.65	144.53 ± 160.62	0.62 [-0.29, 1.50]	50	0.77
tortuosity (DM) – median	0.3749	1.52 ± 0.18	2.04 ± 1.50	-0.42 [-0.80, 0.63]	60	0.63
tortuosity (DM) – interquartile range	0.2633	1.55 ± 0.55	1.97 ± 1.04	-0.46 [-1.14, 0.48]	45	0.66
tortuosity (DM) – range	0.0698	91.95 ± 39.13	61.19 ± 42.29	0.72 [-0.50, 1.70]	70	0.75
tortuosity (SOAM) – mean (mm^{-1})	0.3749	9.27 ± 1.26	10.96 ± 3.17	-0.62 [-1.25, 0.16]	60	0.63
tortuosity (SOAM) – mean (rad/mm)	0.1535	75.76 ± 19.25	92.46 ± 21.31	-0.78 [-1.63, 0.01]	60	0.70
tortuosity (SOAM) – variance (rad^2/mm^2)	0.1137	2.20 ± 0.31	1.93 ± 0.37	0.75 [-0.20, 1.92]	60	0.72
tortuosity (SOAM) – skewness	0.0972	6.68 ± 1.96	5.23 ± 2.38	0.63 [-0.43, 2.01]	50	0.73
tortuosity (SOAM) – kurtosis	0.2030	6.84 ± 1.51	9.11 ± 4.37	-0.61 [-1.20, 0.29]	60	0.68
tortuosity (SOAM) – median (rad/mm)	0.0698	10.14 ± 1.99	12.37 ± 2.84	-0.84 [-1.53, 0.02]	60	0.75
tortuosity (SOAM) – interquartile range (rad/mm)	0.1535	52.30 ± 3.09	56.43 ± 6.62	-0.72 [-1.50, 0.14]	60	0.70
velocity – mean (mm/s)	0.0826	2.59 ± 0.18	2.44 ± 0.19	0.78 [-0.04, 1.53]	60	0.74
velocity – variance (mm^2/s^2)	0.1770	2.51 ± 0.25	2.68 ± 0.28	-0.59 [-1.48, 0.53]	60	0.69
velocity – skewness	0.2633	0.98 ± 0.10	1.18 ± 0.36	-0.65 [-1.20, 0.18]	35	0.66
velocity – kurtosis	0.2633	0.26 ± 0.28	0.94 ± 1.48	-0.56 [-0.98, 0.27]	60	0.66
velocity – median (mm/s)	0.0339	2.34 ± 0.21	2.09 ± 0.22	1.13 [0.19, 1.97]	65	0.79
velocity – interquartile range (mm/s)	0.3348	2.33 ± 0.19	2.38 ± 0.35	-0.18 [-1.14, 0.75]	60	0.64
velocity – range (mm/s)	0.3348	7.01 ± 0.07	6.89 ± 0.34	0.41 [-0.61, 0.85]	60	0.64
velocity range – mean (mm/s)	0.0122	2.43 ± 0.12	2.16 ± 0.25	1.20 [0.49, 1.94]	70	0.84
velocity range – variance (mm^2/s^2)	0.0972	2.41 ± 0.16	2.19 ± 0.31	0.81 [-0.07, 1.44]	70	0.73
velocity range – skewness	0.0698	1.10 ± 0.29	1.46 ± 0.43	-0.90 [-1.50, 0.03]	45	0.75
velocity range – kurtosis	0.0587	0.78 ± 0.74	2.41 ± 2.10	-0.91 [-1.54, -0.33]	50	0.76
velocity range – median (mm/s)	0.0122	2.16 ± 0.17	1.82 ± 0.33	1.16 [0.33, 2.11]	75	0.84
velocity range – interquartile range (mm/s)	0.0062	2.32 ± 0.10	2.07 ± 0.20	1.41 [0.77, 2.17]	65	0.88
velocity range – range (mm/s)	0.5628	6.94 ± 0.15	6.79 ± 0.50	0.36 [-0.47, 1.02]	60	0.58
fractal dimension	0.0049	1.53 ± 0.08	1.36 ± 0.17	1.20 [0.32, 1.82]	85	0.89
fractal R^2	0.2976	1.00 ± 0.00	1.00 ± 0.01	0.23 [-0.66, 1.49]	60	0.65

Supplementary Table 11: Sensitivity analysis of feature selection based on ULM. Values indicate the number of times that features were selected by LASSO across the 20 LOOCV folds.

Individual measurements

1

skewness of tortuosity (DM)	16
range of distances	13
compensated vessel coverage	5
median tortuosity (DM)	3
• skewness of velocity range<br>• kurtosis of velocity range	2
• skewness of distances<br>• median distance<br>• range of track lengths<br>• IQR of tortuosity (DM)	1

2

median track length	12
• kurtosis of distances<br>• variance of tortuosity (SOAM)<br>• skewness of velocity range	3
• kurtosis of track lengths<br>• IQR of tortuosity (DM)<br>• kurtosis of velocity range	1

3

median track length	7
• mean tortuosity (DM)<br>• variance of tortuosity (DM)	6
variance of velocity	4
• range of distances<br>• kurtosis of track lengths<br>• range of velocity ranges	3
• skewness of velocity ranges<br>• kurtosis of velocity ranges	2
• mean track length<br>• IQR of tortuosity (SOAM)<br>• median velocity<br>• IQR of velocity	1

Changes between measurements

1 → 2

compensated vessel coverage ($\|\Delta F\|$)	20
skewness of distances ($\|\Delta F\|$)	17
mean tortuosity (SOAM) (F_{min})	16
compensated vessel coverage (F_{max})	14
variance of tortuosity (SOAM) (F_{max})	10
median tortuosity (SOAM) (F_{min})	5
• range of track lengths ($\|\Delta F\|$)<br>• median track length (F_{max})	4
• kurtosis of tortuosity (DM) (ΔF)<br>• range of tortuosity (DM) (ΔF)	2
• kurtosis of distances ($\|\Delta F\|$)<br>• mean track length ($\|\Delta F\|$)<br>• fractal dimension ($\|\Delta F\|$)<br>• IQR of tortuosity (DM) (F_{min})	1

2 → 3

median tortuosity (DM) (F_{min})	17
median track length (F_{max})	16
• kurtosis of distances ($\|\Delta F\|$)<br>• IQR of track lengths (F_{max})	10
compensated vessel coverage (F_{max})	9
median tortuosity (SOAM)) (F_{min})	8
variance of tortuosity (DM) (ΔF)	7
IQR of velocity ranges (F_{max})	5
IQR of tortuosity (DM) (F_{min})	4
median velocity ($\|\Delta F\|$)	3
• skewness of tortuosity (SOAM) (F_{max})<br>• kurtosis of tortuosity (SOAM) (F_{max})	2
• mean tortuosity (DM) (ΔF)<br>• median tortuosity (DM) (ΔF)<br>• tortuosity (DM) (ΔF)<br>• variance of velocity (ΔF)<br>• IQR of velocity (ΔF)<br>• compensated vessel coverage ($\|\Delta F\|$)<br>• skewness of distances ($\|\Delta F\|$)<br>• median velocity range ($\|\Delta F\|$)<br>• fractal dimension ($\|\Delta F\|$)<br>• IQR of tortuosity (SOAM) (F_{min})<br>• variance of velocity ranges (F_{max})	1

1 → 3

range of distances (F_{min})	19
range of tortuosity (DM) ($\|\Delta F\|$)	17
mean tortuosity (DM) (ΔF)	15
kurtosis of tortuosity (DM) (ΔF)	14
IQR of tortuosity (SOAM) (F_{max})	7
IQR of velocity ranges (F_{max})	5
• variance of tortuosity (DM) ($\|\Delta F\|$)<br>• range of tortuosity (SOAM) ($\|\Delta F\|$)	4
median velocity range (F_{max})	3
• kurtosis of velocity ranges (F_{min})<br>• variance of velocities (F_{max})<br>• skewness of velocity ranges (F_{max})	2
• skewness of tortuosity (DM) (ΔF)<br>• fractal dimension (ΔF)<br>• median distance ($\|\Delta F\|$)<br>• median distance (F_{min})<br>• variance of tortuosity (DM) (F_{max})	1

Combined measurements

1 & 2

compensated vessel coverage ($\|\Delta F\|$)	20
skewness of distances ($\|\Delta F\|$)	17
mean tortuosity (SOAM) (F_{min})	14
compensated vessel coverage (F_{max})	13
variance of tortuosity (SOAM) (F_{max})	8
• range of track lengths ($\|\Delta F\|$)<br>• median tortuosity (SOAM) (F_{min})<br>• median track length (F_{max})	4
• skewness of velocity ranges ($F^{(2)}$)<br>• kurtosis of tortuosity (DM) (ΔF)<br>• range of tortuosity (DM) (ΔF)<br>• kurtosis of distances ($\|\Delta F\|$)	2

1 & 2 & 3

compensated vessel coverage ($\|\Delta F^{(1,2)}\|$)	20
skewness of distances ($\|\Delta F^{(1,2)}\|$)	15
compensated vessel coverage ($F_{max}^{(1,2)}$)	14
median tortuosity (SOAM) ($F_{min}^{(2,3)}$)	12
mean tortuosity (SOAM) ($F_{min}^{(1,2)}$)	11
• median track length ($F_{max}^{(1,2,3)}$)<br>• IQR of velocity ranges ($F_{max}^{(1,2,3)}$)	4
• mean tortuosity (SOAM) ($F_{min}^{(1,2,3)}$)<br>• median tortuosity (SOAM) ($F_{min}^{(1,2,3)}$)	2
• median tortuosity (SOAM) ($F^{(2)}$)<br>• range of tortuosity (DM) ($\Delta F^{(1,2)}$)<br>• kurtosis of distances ($\|\Delta F^{(1,2)}\|$)	1

• median tortuosity (SOAM) ($F^{(2)}$)<br>• IQR of tortuosity (DM) ($F_{\min}$)	1	• range of track lengths ($\lvert\Delta F^{(1,2)}\rvert$)<br>• median tortuosity (SOAM) ($F_{\min}{}^{(1,2)}$)<br>• skewness of velocity ranges ($F_{\min}{}^{(1,2)}$)<br>• median track length ($F_{\max}{}^{(1,2)}$)<br>• range of track lengths ($F_{\max}{}^{(1,2)}$)<br>• variance of tortuosity (SOAM) ($F_{\max}{}^{(1,2)}$)<br>• range of distances ($F_{\min}{}^{(1,3)}$)<br>• median tortuosity (DM) ($F_{\min}{}^{(2,3)}$)<br>• median track length ($F_{\max}{}^{(2,3)}$)<br>• IQR of velocity ranges ($F_{\max}{}^{(2,3)}$)<br>• skewness of velocity ranges ($F_{\min}{}^{(1,2,3)}$)<br>• compensated vessel coverage ($F_{\max}{}^{(1,2,3)}$)

DM: distance metric; IQR: interquartile range; SOAM: sum-of-angles metric

Supplementary Table 12: Comparison of feature selection probability among ULM and comparative methods. Values represent the number of features that showed a selection probability ≥ 50% (left value) or ≥ 75% (right value) during LOOCV.

	Individual measurements			Changes between measurements			Combined measurements	
**Method**	**1**	**2**	**3**	**1 → 2**	**2 → 3**	**1 → 3**	**1 & 2**	**1 & 2 & 3**
ULM	2 | 1	1 | 0	0 | 0	5 | 3	4 | 2	4 | 3	4 | 2	5 | 2
B-mode size measurement	1 | 1	1 | 1	1 | 1	0 | 0	1 | 1	1 | 1	1 | 0	1 | 0
B-mode image	0 | 0	0 | 0	0 | 0	0 | 0	3 | 1	0 | 0	0 | 0	3 | 1
Contrast-mode MIP	0 | 0	0 | 0	1 | 0	2 | 2	1 | 0	2 | 0	2 | 2	4 | 2
Histological CD31 masks	3 | 2							

Supplementary Table 13: Number of features with large effect sizes or significant nominal p-values among ULM or comparative methods.

	Individual measurements			Changes between measurements		
**Method**	**1**	**2**	**3**	**1 → 2**	**2 → 3**	**1 → 3**
***# of features with* $\|g\| \geq 0.8$**						
ULM	11 (24%)	7 (15%)	0 (0%)	43 (23%)	28 (15%)	15 (8%)
B-mode size measurement	0 (0%)	0 (0%)	0 (0%)	0 (0%)	0 (0%)	0 (0%)
B-mode image	0 (0%)	0 (0%)	0 (0%)	1 (3%)	5 (14%)	2 (6%)
Contrast-mode MIP	3 (18%)	0 (0%)	0 (0%)	24 (35%)	1 (1%)	7 (10%)
Histological CD31 mask	5 (50%)					
***# of features with* $p < 0.05$**						
ULM	9 (20%)	7 (15%)	0 (0%)	40 (22%)	20 (11%)	9 (5%)
B-mode size measurement	0 (0%)	0 (0%)	0 (0%)	0 (0%)	0 (0%)	0 (0%)
B-mode image	0 (0%)	0 (0%)	0 (0%)	0 (0%)	2 (6%)	1 (3%)
Contrast-mode MIP	1 (6%)	0 (0%)	0 (0%)	12 (18%)	0 (0%)	9 (13%)
Histological CD31 mask	7 (70%)					

Supplementary Table 14: Summary statistics of features extracted from B-mode size measurements for the three individual measurements. From left to right: feature name, p-value for a statistical significance between patients with pCR and non-pCR, mean ± standard deviation of patients with pCR, mean ± standard deviation of patients with non-pCR, effect size expressed by Hedges' g with bootstrapped 95% confidence interval, cross-validated predictive accuracy, area under the receiver operating characteristic curve (AUC).

	Measurement 1						Measurement 2						Measurement 3					
**Feature**	**p**	**pCR**	**non-pCR**	**Effect size**	**Acc (%)**	**AUC**	**p**	**pCR**	**non-pCR**	**Effect size**	**Acc (%)**	**AUC**	**p**	**pCR**	**non-pCR**	**Effect size**	**Acc (%)**	**AUC**
tumour volume (cm^3)	0.7871	2.33 ± 1.32	7.42 ± 10.87	-0.57 [-1.12, 0.14]	60	0.54	0.9079	1.42 ± 0.98	5.30 ± 7.50	-0.63 [-1.27, -0.09]	60	0.52	0.2030	0.65 ± 0.69	3.93 ± 6.27	-0.64 [-1.12, -0.14]	30	0.68

Supplementary Table 15: Summary statistics of features extracted from B-mode size measurements for changes between measurements. Delta radiomic features of changes include relative change, absolute change, minimum, and maximum of the two measurements in question. From left to right: feature name, p-value for a statistical significance between patients with pCR and non-pCR, mean ± standard deviation of patients with pCR, mean ± standard deviation of patients with non-pCR, effect size expressed by Hedges' g with bootstrapped 95% confidence interval, cross-validated predictive accuracy, area under the receiver operating characteristic curve (AUC).

	Measurement 1 → 2						Measurement 2 → 3						Measurement 1 → 3					
**Feature**	**p**	**pCR**	**non-pCR**	**Effect size**	**Acc (%)**	**AUC**	**p**	**pCR**	**non-pCR**	**Effect size**	**Acc (%)**	**AUC**	**p**	**pCR**	**non-pCR**	**Effect size**	**Acc (%)**	**AUC**
relative change (ΔF)																		
tumour volume (cm^3)	0.7285	-0.91 ± 1.03	-2.12 ± 4.73	0.31 [-0.46, 1.10]	60	0.55	0.5628	-0.78 ± 0.85	-1.37 ± 2.96	0.24 [-0.92, 0.99]	60	0.58	0.7285	-1.68 ± 0.91	-3.49 ± 4.75	0.46 [-0.41, 1.14]	60	0.55
absolute change ($\lvert\Delta F\rvert$)																		
tumour volume (cm^3)	0.3348	0.91 ± 1.03	2.65 ± 4.42	-0.48 [-0.88, 0.49]	50	0.64	0.9079	0.96 ± 0.60	1.97 ± 2.57	-0.47 [-1.18, 0.32]	60	0.52	0.7285	1.68 ± 0.91	3.49 ± 4.75	-0.46 [-1.19, 0.22]	60	0.55
minimum (F_{min})																		
tumour volume (cm^3)	0.9079	1.42 ± 0.98	5.03 ± 7.40	-0.59 [-1.23, 0.01]	60	0.52	0.2633	0.56 ± 0.56	3.63 ± 5.84	-0.64 [-1.12, -0.22]	30	0.66	0.2030	0.65 ± 0.69	3.93 ± 6.27	-0.64 [-1.22, -0.22]	30	0.68
maximum (F_{max})																		
tumour volume (cm^3)	0.7871	2.33 ± 1.32	7.68 ± 10.88	-0.60 [-1.17, 0.05]	60	0.54	0.8471	1.52 ± 0.97	5.60 ± 7.77	-0.64 [-1.32, -0.13]	60	0.53	0.7871	2.33 ± 1.32	7.42 ± 10.87	-0.57 [-1.14, 0.04]	60	0.54

Supplementary Table 16: Summary statistics of features extracted from B-mode size measurements for the three measurements combined. Delta radiomic features include the minimum and maximum of the three measurements. From left to right: feature name, p-value for a statistical significance between patients with pCR and non-pCR, mean ± standard deviation of patients with pCR, mean ± standard deviation of patients with non-pCR, effect size expressed by Hedges' g with bootstrapped 95% confidence interval, cross-validated predictive accuracy, area under the receiver operating characteristic curve (AUC).

Feature	Measurement 1 & 2 & 3					
	**p**	**pCR**	**non-pCR**	**Effect size**	**Acc (%)**	**AUC**
minimum (F_{min})						
tumour volume (cm^3)	0.2633	0.56 ± 0.56	3.63 ± 5.84	-0.64 [-1.14, -0.22]	60	0.66
maximum (F_{max})						
tumour volume (cm^3)	0.7871	2.33 ± 1.32	7.68 ± 10.88	-0.60 [-1.24, -0.04]	60	0.54

Supplementary Table 17: Summary statistics of features extracted from B-mode ultrasound images for the three individual measurements. From left to right: feature name, p-value for a statistical significance between patients with pCR and non-pCR, mean ± standard deviation of patients with pCR, mean ± standard deviation of patients with non-pCR, effect size expressed by Hedges' g with bootstrapped 95% confidence interval, cross-validated predictive accuracy, area under the receiver operating characteristic curve (AUC).

	Measurement 1						Measurement 2						Measurement 3					
Feature	p	pCR	non-pCR	Effect size	Acc (%)	AUC	p	pCR	non-pCR	Effect size	Acc (%)	AUC	p	pCR	non-pCR	Effect size	Acc (%)	AUC
histogram - mean	0.7871	1.94 ± 0.22	1.99 ± 0.43	-0.13 [-0.90, 0.77]	60	0.54	0.2030	1.94 ± 0.47	2.14 ± 0.42	-0.44 [-1.32, 0.63]	45	0.68	0.4179	2.38 ± 0.39	2.21 ± 0.44	0.39 [-0.48, 1.37]	50	0.61
histogram - variance	0.8471	0.83 ± 0.23	0.82 ± 0.22	0.06 [-0.94, 1.13]	60	0.53	0.1325	0.75 ± 0.24	0.93 ± 0.22	-0.76 [-1.71, 0.28]	60	0.71	0.7285	0.92 ± 0.22	0.85 ± 0.24	0.27 [-0.62, 1.26]	55	0.55
histogram - skewness	0.9079	0.93 ± 0.49	0.93 ± 0.41	0.01 [-1.04, 1.04]	60	0.52	0.7871	0.91 ± 0.60	0.88 ± 0.67	0.04 [-0.88, 0.95]	60	0.54	0.9692	0.59 ± 0.23	0.65 ± 0.38	-0.18 [-0.95, 0.72]	60	0.51
histogram - kurtosis	0.7871	1.01 ± 1.29	0.72 ± 1.02	0.25 [-0.71, 1.35]	50	0.54	0.8471	0.94 ± 1.38	1.17 ± 2.30	-0.11 [-0.80, 0.90]	60	0.53	0.9079	0.45 ± 0.71	0.33 ± 0.67	0.16 [-0.78, 1.13]	60	0.52
histogram - median	0.4064	2.00 ± 0.00	1.83 ± 0.58	0.35 [-0.64, 0.99]	60	0.58	0.2125	1.63 ± 0.74	2.00 ± 0.60	-0.54 [-1.60, 0.50]	50	0.66	0.3632	2.25 ± 0.46	2.00 ± 0.60	0.43 [-0.42, 1.27]	50	0.60
histogram - interquartile range	0.5407	1.13 ± 0.35	1.25 ± 0.45	-0.29 [-0.93, 0.95]	60	0.56	0.8517	1.13 ± 0.35	1.17 ± 0.39	-0.11 [-0.72, 1.26]	60	0.52	0.3290	1.38 ± 0.52	1.17 ± 0.39	0.45 [-0.52, 1.66]	50	0.60
histogram - range	0.5571	5.13 ± 0.83	4.92 ± 0.67	0.27 [-0.70, 1.21]	50	0.58	0.1809	4.75 ± 0.89	5.33 ± 0.89	-0.63 [-1.63, 0.27]	50	0.68	0.2342	5.38 ± 0.92	4.92 ± 0.79	0.52 [-0.49, 1.64]	45	0.66
fractal dimension	0.7285	1.84 ± 0.01	1.84 ± 0.01	0.15 [-0.74, 0.93]	60	0.55	0.4179	1.83 ± 0.02	1.84 ± 0.01	-0.47 [-1.65, 0.36]	60	0.61	0.5890	1.84 ± 0.02	1.84 ± 0.01	0.02 [-1.29, 1.12]	60	0.58
fractal R^2	0.7871	1.00 ± 0.00	1.00 ± 0.00	0.19 [-0.90, 1.02]	55	0.54	0.4177	1.00 ± 0.00	1.00 ± 0.00	-0.58 [-1.80, 0.39]	60	0.61	0.6433	1.00 ± 0.00	1.00 ± 0.00	0.15 [-0.90, 1.00]	60	0.57

Supplementary Table 18: Summary statistics of features extracted from B-mode ultrasound images for changes between measurements. Delta radiomic features of changes include relative change, absolute change, minimum, and maximum of the two measurements in question. From left to right: feature name, p-value for a statistical significance between patients with pCR and non-pCR, mean ± standard deviation of patients with pCR, mean ± standard deviation of patients with non-pCR, effect size expressed by Hedges' g with bootstrapped 95% confidence interval, cross-validated predictive accuracy, area under the receiver operating characteristic curve (AUC).

	Measurement 1 → 2						Measurement 2 → 3						Measurement 1 → 3					
Feature	p	pCR	non-pCR	Effect size	Acc (%)	AUC	p	pCR	non-pCR	Effect size	Acc (%)	AUC	p	pCR	non-pCR	Effect size	Acc (%)	AUC
relative change (ΔF)																		
histogram - mean	0.3749	0.00 ± 0.40	0.15 ± 0.42	-0.35 [-1.34, 0.56]	50	0.63	0.0826	0.44 ± 0.33	0.06 ± 0.43	0.90 [-0.07, 1.81]	65	0.74	0.7285	0.44 ± 0.32	0.22 ± 0.60	0.42 [-0.34, 1.21]	60	0.55
histogram - variance	0.0826	-0.09 ± 0.20	0.11 ± 0.22	-0.88 [-1.68, 0.02]	50	0.74	0.0097	0.17 ± 0.20	-0.08 ± 0.17	1.28 [-0.09, 2.39]	75	0.85	0.7285	0.08 ± 0.19	0.03 ± 0.23	0.23 [-0.62, 1.09]	60	0.55
histogram - skewness	0.6160	-0.03 ± 0.61	-0.05 ± 0.59	0.03 [-0.94, 1.20]	60	0.57	0.7871	-0.32 ± 0.54	-0.23 ± 0.57	-0.15 [-1.21, 0.70]	60	0.54	0.7871	-0.34 ± 0.51	-0.28 ± 0.45	-0.13 [-1.26, 0.73]	60	0.54
histogram - kurtosis	0.8471	-0.07 ± 1.78	0.46 ± 2.16	-0.25 [-1.09, 0.91]	60	0.53	0.9079	-0.50 ± 1.62	-0.84 ± 2.08	0.17 [-0.82, 0.93]	60	0.52	0.7285	-0.56 ± 1.43	-0.38 ± 0.85	-0.16 [-1.24, 1.00]	60	0.55
histogram - median	0.1256	-0.38 ± 0.74	0.17 ± 0.72	-0.71 [-1.65, 0.49]	65	0.70	0.0336	0.63 ± 0.52	0.00 ± 0.60	1.05 [-0.06, 2.06]	75	0.76	0.8942	0.25 ± 0.46	0.17 ± 0.72	0.13 [-0.73, 1.08]	60	0.52
histogram - interquartile range	0.7602	0.00 ± 0.53	-0.08 ± 0.51	0.15 [-0.86, 1.09]	55	0.54	0.2451	0.25 ± 0.46	0.00 ± 0.43	0.54 [-0.37, 1.36]	55	0.61	0.1544	0.25 ± 0.71	-0.08 ± 0.29	0.64 [-1.17, 1.74]	75	0.65
histogram - range	0.1974	-0.38 ± 1.19	0.42 ± 1.00	-0.71 [-1.64, 0.20]	50	0.67	0.0864	0.63 ± 1.06	-0.42 ± 1.16	0.89 [-0.05, 1.87]	45	0.73	0.6833	0.25 ± 1.04	0.00 ± 1.04	0.23 [-0.79, 1.23]	60	0.56
fractal dimension	0.2976	-0.01 ± 0.01	0.00 ± 0.01	-0.61 [-1.52, 0.37]	60	0.65	0.1535	0.00 ± 0.01	0.00 ± 0.01	0.56 [-0.51, 1.61]	50	0.70	0.9692	0.00 ± 0.02	0.00 ± 0.02	-0.09 [-1.17, 0.90]	60	0.51
fractal R^2	0.2976	0.00 ± 0.00	0.00 ± 0.00	-0.70 [-1.53, 0.22]	50	0.65	0.0972	0.00 ± 0.00	0.00 ± 0.00	0.81 [0.00, 1.44]	65	0.73	0.5628	0.00 ± 0.00	0.00 ± 0.00	-0.09 [-0.83, 0.82]	60	0.58
absolute change (|ΔF|)																		
histogram - mean	0.7871	0.32 ± 0.20	0.36 ± 0.24	-0.17 [-1.19, 0.68]	60	0.54	0.2318	0.49 ± 0.23	0.34 ± 0.26	0.59 [-0.33, 1.59]	40	0.67	0.3348	0.44 ± 0.32	0.54 ± 0.31	-0.30 [-1.23, 0.73]	50	0.64
histogram - variance	0.5628	0.18 ± 0.11	0.17 ± 0.17	0.03 [-0.81, 1.18]	60	0.58	0.1325	0.23 ± 0.12	0.17 ± 0.07	0.66 [-0.59, 1.71]	45	0.71	0.5120	0.16 ± 0.12	0.18 ± 0.14	-0.18 [-0.99, 0.85]	60	0.59

	Measurement 1 → 2						Measurement 2 → 3						Measurement 1 → 3					
**Feature**	**p**	**pCR**	**non-pCR**	**Effect size**	**Acc (%)**	**AUC**	**p**	**pCR**	**non-pCR**	**Effect size**	**Acc (%)**	**AUC**	**p**	**pCR**	**non-pCR**	**Effect size**	**Acc (%)**	**AUC**
histogram - skewness	0.4636	0.47 ± 0.34	0.39 ± 0.42	0.19 [-0.76, 1.13]	60	0.60	0.8471	0.45 ± 0.41	0.40 ± 0.45	0.12 [-0.74, 1.14]	60	0.53	0.7285	0.48 ± 0.37	0.41 ± 0.32	0.18 [-0.79, 1.19]	60	0.55
histogram - kurtosis	0.6160	1.35 ± 1.04	1.33 ± 1.73	0.02 [-0.86, 1.11]	55	0.57	1.0000	1.11 ± 1.22	1.26 ± 1.83	-0.09 [-0.80, 1.01]	60	0.50	0.2030	1.23 ± 0.84	0.70 ± 0.59	0.72 [-0.35, 1.98]	50	0.68
histogram - median	0.6228	0.63 ± 0.52	0.50 ± 0.52	0.23 [-0.64, 1.18]	60	0.56	0.2273	0.63 ± 0.52	0.33 ± 0.49	0.56 [-0.38, 1.74]	40	0.65	0.2963	0.25 ± 0.46	0.50 ± 0.52	-0.48 [-1.43, 0.45]	55	0.63
histogram - interquartile range	1.0000	0.25 ± 0.46	0.25 ± 0.45	0.00 [-0.77, 1.12]	60	0.50	0.6971	0.25 ± 0.46	0.17 ± 0.39	0.19 [-0.62, 1.32]	50	0.54	0.0451	0.50 ± 0.53	0.08 ± 0.29	0.99 [-0.26, 2.49]	75	0.71
histogram - range	0.7717	0.88 ± 0.83	0.75 ± 0.75	0.15 [-0.77, 1.12]	60	0.54	0.9344	0.88 ± 0.83	0.92 ± 0.79	-0.05 [-1.00, 0.88]	60	0.52	0.7688	0.75 ± 0.71	0.67 ± 0.78	0.11 [-0.78, 1.12]	60	0.54
fractal dimension	0.6713	0.01 ± 0.01	0.01 ± 0.01	0.07 [-1.07, 1.68]	60	0.56	0.7871	0.01 ± 0.01	0.01 ± 0.01	0.23 [-0.69, 1.31]	60	0.54	0.9079	0.01 ± 0.01	0.01 ± 0.01	0.10 [-0.86, 1.18]	60	0.52
fractal R^2	0.3348	0.00 ± 0.00	0.00 ± 0.00	0.40 [-0.52, 1.52]	60	0.64	0.7871	0.00 ± 0.00	0.00 ± 0.00	0.27 [-0.93, 2.13]	60	0.54	0.3749	0.00 ± 0.00	0.00 ± 0.00	-0.51 [-1.06, 0.39]	60	0.63
minimum (F_{min})																		
histogram - mean	0.3348	1.78 ± 0.26	1.89 ± 0.34	-0.32 [-1.28, 0.63]	60	0.64	0.3348	1.92 ± 0.46	2.01 ± 0.37	-0.21 [-1.15, 0.90]	60	0.64	0.6160	1.94 ± 0.22	1.83 ± 0.27	0.42 [-0.45, 1.33]	60	0.57
histogram - variance	0.2318	0.70 ± 0.22	0.79 ± 0.20	-0.40 [-1.40, 0.70]	50	0.67	0.2976	0.72 ± 0.23	0.80 ± 0.20	-0.40 [-1.28, 0.69]	55	0.65	0.7871	0.80 ± 0.22	0.74 ± 0.24	0.22 [-0.73, 1.26]	60	0.54
histogram - skewness	0.9692	0.68 ± 0.50	0.71 ± 0.38	-0.06 [-1.02, 0.99]	60	0.51	0.7285	0.52 ± 0.31	0.56 ± 0.34	-0.13 [-0.94, 0.90]	60	0.55	0.8471	0.52 ± 0.24	0.58 ± 0.30	-0.21 [-0.92, 0.96]	60	0.53
histogram - kurtosis	1.0000	0.30 ± 0.76	0.28 ± 0.84	0.03 [-0.82, 1.05]	60	0.50	0.6713	0.14 ± 0.58	0.12 ± 0.69	0.03 [-0.76, 1.06]	60	0.56	0.7285	0.12 ± 0.58	0.17 ± 0.63	-0.09 [-0.87, 0.90]	60	0.55
histogram - median	0.4958	1.50 ± 0.53	1.67 ± 0.49	-0.31 [-1.36, 0.64]	60	0.58	0.4352	1.63 ± 0.74	1.83 ± 0.58	-0.31 [-1.40, 0.75]	45	0.60	0.0847	2.00 ± 0.00	1.67 ± 0.49	0.83 [0.41, 1.61]	35	0.67
histogram - interquartile range	0.4750	1.00 ± 0.00	1.08 ± 0.29	-0.35 [-0.57, -0.25]	55	0.54	0.8240	1.13 ± 0.35	1.08 ± 0.29	0.13 [-0.64, 1.11]	55	0.52	0.2661	1.00 ± 0.00	1.17 ± 0.39	-0.52 [-1.12, -0.28]	60	0.58

Feature	Measurement 1 → 2						Measurement 2 → 3						Measurement 1 → 3					
	p	pCR	non-pCR	Effect size	Acc (%)	AUC	p	pCR	non-pCR	Effect size	Acc (%)	AUC	p	pCR	non-pCR	Effect size	Acc (%)	AUC
histogram - range	0.2535	4.50 ± 0.76	4.75 ± 0.45	-0.41 [-1.59, 1.06]	45	0.64	0.7349	4.63 ± 0.92	4.67 ± 0.65	-0.05 [-1.04, 1.18]	60	0.55	0.4497	4.88 ± 0.83	4.58 ± 0.67	0.38 [-0.56, 1.49]	60	0.60
fractal dimension	0.6713	1.83 ± 0.02	1.84 ± 0.01	-0.19 [-1.36, 0.70]	60	0.56	0.7285	1.83 ± 0.02	1.84 ± 0.01	-0.29 [-1.58, 0.73]	60	0.55	0.9692	1.83 ± 0.01	1.83 ± 0.01	0.04 [-0.90, 1.06]	60	0.51
fractal R^2	0.3749	1.00 ± 0.00	1.00 ± 0.00	-0.35 [-1.36, 0.56]	55	0.63	0.8471	1.00 ± 0.00	1.00 ± 0.00	-0.35 [-2.13, 0.79]	60	0.53	0.5890	1.00 ± 0.00	1.00 ± 0.00	0.34 [-0.53, 1.00]	60	0.58
maximum (F_{max})																		
histogram - mean	0.3348	2.10 ± 0.38	2.25 ± 0.43	-0.34 [-1.27, 0.65]	50	0.64	1.0000	2.41 ± 0.36	2.35 ± 0.42	0.15 [-0.68, 1.09]	60	0.50	0.9079	2.38 ± 0.39	2.37 ± 0.42	0.03 [-0.79, 1.15]	60	0.52
histogram - variance	0.5628	0.88 ± 0.22	0.96 ± 0.22	-0.35 [-1.37, 0.65]	60	0.58	0.9692	0.95 ± 0.19	0.97 ± 0.23	-0.12 [-0.98, 0.81]	60	0.51	0.8471	0.95 ± 0.21	0.93 ± 0.18	0.14 [-0.83, 1.23]	60	0.53
histogram - skewness	0.6713	1.15 ± 0.47	1.10 ± 0.62	0.09 [-0.81, 1.05]	60	0.56	0.7871	0.97 ± 0.50	0.96 ± 0.64	0.01 [-0.89, 1.00]	60	0.54	1.0000	1.00 ± 0.42	0.99 ± 0.41	0.01 [-0.90, 0.99]	60	0.50
histogram - kurtosis	0.6160	1.66 ± 1.39	1.61 ± 2.18	0.02 [-0.73, 1.08]	60	0.57	0.6713	1.25 ± 1.22	1.39 ± 2.18	-0.07 [-0.82, 1.04]	60	0.56	0.3348	1.34 ± 1.07	0.87 ± 0.94	0.45 [-0.52, 1.57]	50	0.64
histogram - median	0.8383	2.13 ± 0.35	2.17 ± 0.58	-0.08 [-0.89, 0.96]	60	0.53	0.8099	2.25 ± 0.46	2.17 ± 0.58	0.15 [-0.73, 1.10]	60	0.53	0.8099	2.25 ± 0.46	2.17 ± 0.58	0.15 [-0.75, 1.10]	60	0.53
histogram - interquartile range	0.7340	1.25 ± 0.46	1.33 ± 0.49	-0.17 [-0.95, 0.88]	60	0.54	0.5934	1.38 ± 0.52	1.25 ± 0.45	0.25 [-0.67, 1.34]	45	0.56	0.2834	1.50 ± 0.53	1.25 ± 0.45	0.49 [-0.50, 1.69]	45	0.63
histogram - range	0.7373	5.38 ± 0.74	5.50 ± 0.90	-0.14 [-1.17, 0.71]	60	0.55	0.9316	5.50 ± 0.76	5.58 ± 0.79	-0.10 [-1.05, 0.79]	60	0.52	0.1578	5.63 ± 0.74	5.25 ± 0.62	0.53 [-0.55, 1.53]	40	0.68
fractal dimension	0.2028	1.84 ± 0.01	1.85 ± 0.01	-0.27 [-1.33, 0.61]	60	0.68	0.9385	1.84 ± 0.01	1.84 ± 0.01	-0.22 [-1.31, 0.87]	55	0.52	0.6996	1.85 ± 0.01	1.85 ± 0.01	0.24 [-0.92, 1.07]	60	0.56
fractal R^2	0.6713	1.00 ± 0.00	1.00 ± 0.00	-0.10 [-1.19, 0.72]	60	0.56	0.7868	1.00 ± 0.00	1.00 ± 0.00	-0.47 [-1.53, 0.53]	50	0.54	0.5628	1.00 ± 0.00	1.00 ± 0.00	-0.23 [-1.45, 0.84]	60	0.58

Supplementary Table 19: Summary statistics of features extracted from B-mode ultrasound images for the three measurements combined. Delta radiomic features include the minimum and maximum of the three measurements. From left to right: feature name, p-value for a statistical significance between patients with pCR and non-pCR, mean ± standard deviation of patients with pCR, mean ± standard deviation of patients with non-pCR, effect size expressed by Hedges' g with bootstrapped 95% confidence interval, cross-validated predictive accuracy, area under the receiver operating characteristic curve (AUC).

Feature	Measurement 1 & 2 & 3					
	p	pCR	non-pCR	Effect size	Acc (%)	AUC
minimum (F_{min})						
histogram - mean	0.4636	1.78 ± 0.26	1.78 ± 0.26	-0.17 [-1.19, 0.73]	60	0.60
histogram - variance	0.6160	0.69 ± 0.21	0.69 ± 0.21	-0.19 [-1.13, 0.76]	60	0.57
histogram - skewness	0.9692	0.47 ± 0.30	0.47 ± 0.30	-0.14 [-1.05, 0.99]	60	0.51
histogram - kurtosis	0.9692	0.01 ± 0.60	0.01 ± 0.60	0.03 [-0.70, 1.09]	60	0.51
histogram - median	0.4958	1.50 ± 0.53	1.50 ± 0.53	-0.31 [-1.36, 0.77]	55	0.58
histogram - interquartile range	0.4750	1.00 ± 0.00	1.00 ± 0.00	-0.35 [-0.58, -0.25]	55	0.54
histogram - range	0.8262	4.50 ± 0.76	4.50 ± 0.76	0.00 [-1.05, 1.25]	60	0.53
fractal dimension	0.9692	1.83 ± 0.02	1.83 ± 0.02	-0.19 [-1.28, 0.90]	55	0.51
fractal R^2	0.9079	1.00 ± 0.00	1.00 ± 0.00	-0.17 [-1.69, 0.74]	60	0.52
maximum (F_{max})						
histogram - mean	0.8471	2.41 ± 0.36	2.45 ± 0.38	-0.10 [-0.93, 0.95]	60	0.53
histogram - variance	0.9692	0.97 ± 0.19	0.99 ± 0.22	-0.10 [-1.03, 0.88]	60	0.51
histogram - skewness	0.5628	1.17 ± 0.44	1.11 ± 0.61	0.11 [-0.70, 1.19]	60	0.58
histogram - kurtosis	0.3348	1.86 ± 1.23	1.64 ± 2.15	0.11 [-0.82, 1.22]	55	0.64
histogram - median	0.7340	2.25 ± 0.46	2.33 ± 0.49	-0.17 [-0.98, 0.86]	60	0.54
histogram - interquartile range	0.4958	1.50 ± 0.53	1.33 ± 0.49	0.31 [-0.65, 1.39]	55	0.58
histogram - range	0.8909	5.75 ± 0.46	5.67 ± 0.78	0.12 [-0.74, 0.93]	60	0.52
fractal dimension	0.6432	1.85 ± 0.01	1.85 ± 0.00	-0.19 [-1.42, 0.76]	60	0.57
fractal R^2	0.5628	1.00 ± 0.00	1.00 ± 0.00	-0.32 [-1.64, 0.70]	60	0.58

Supplementary Table 20: Summary statistics of features extracted from contrast-mode MIPs for the three individual measurements. From left to right: feature name, p-value for a statistical significance between patients with pCR and non-pCR, mean ± standard deviation of patients with pCR, mean ± standard deviation of patients with non-pCR, effect size expressed by Hedges' g with bootstrapped 95% confidence interval, cross-validated predictive accuracy, area under the receiver operating characteristic curve (AUC).

	Measurement 1						Measurement 2						Measurement 3					
**Feature**	**p**	**pCR**	**non-pCR**	**Effect size**	**Acc (%)**	**AUC**	**p**	**pCR**	**non-pCR**	**Effect size**	**Acc (%)**	**AUC**	**p**	**pCR**	**non-pCR**	**Effect size**	**Acc (%)**	**AUC**
coverage (%)	0.0821	17.12 ± 17.09	6.71 ± 9.39	0.77 [-0.24, 1.67]	60	0.74	0.8471	13.67 ± 24.30	7.86 ± 13.07	0.30 [-0.53, 1.46]	55	0.53	0.2030	12.89 ± 14.69	6.36 ± 10.30	0.51 [-0.51, 1.48]	55	0.68
distances – mean (mm)	0.1763	0.82 ± 0.59	2.19 ± 1.92	-0.85 [-1.61, -0.10]	60	0.73	0.4636	1.64 ± 1.42	2.01 ± 1.64	-0.23 [-1.04, 0.75]	60	0.57	0.1535	1.00 ± 0.90	1.93 ± 1.70	-0.63 [-1.34, 0.29]	60	0.67
distances – variance (mm^2)	0.1932	0.82 ± 0.99	4.74 ± 6.51	-0.73 [-1.28, -0.08]	65	0.70	0.5999	1.73 ± 1.75	4.04 ± 5.28	-0.53 [-1.14, 0.33]	58	0.58	0.2723	1.03 ± 1.50	3.12 ± 4.81	-0.52 [-1.07, 0.27]	58	0.66
distances – skewness	0.4747	1.13 ± 0.52	0.94 ± 0.47	0.38 [-0.71, 1.54]	35	0.61	1.0000	1.09 ± 1.27	0.80 ± 0.53	0.30 [-0.76, 1.68]	58	0.50	0.6574	0.94 ± 0.50	0.86 ± 0.75	0.11 [-0.77, 1.04]	58	0.57
distances – kurtosis	0.4173	0.84 ± 1.53	0.24 ± 1.17	0.42 [-0.62, 1.66]	35	0.63	0.6574	2.05 ± 5.75	0.06 ± 1.34	0.50 [-0.62, 1.66]	42	0.57	0.9039	0.37 ± 1.27	0.61 ± 2.20	-0.12 [-1.02, 0.89]	58	0.52
distances – median (mm)	0.1529	0.59 ± 0.51	1.81 ± 1.75	-0.83 [-1.62, -0.06]	60	0.74	0.5628	1.51 ± 1.43	1.64 ± 1.34	-0.09 [-0.97, 0.89]	60	0.55	0.1425	0.82 ± 0.78	1.65 ± 1.58	-0.61 [-1.23, 0.43]	60	0.68
distances – interquartile range (mm)	0.1932	1.08 ± 0.78	2.66 ± 2.42	-0.77 [-1.40, 0.02]	53	0.70	0.5999	1.70 ± 1.39	2.42 ± 2.08	-0.38 [-1.26, 0.47]	58	0.58	0.3511	1.23 ± 1.19	2.12 ± 1.98	-0.50 [-1.16, 0.52]	53	0.64
distances – range (mm)	0.1613	3.25 ± 1.90	7.37 ± 5.48	-0.89 [-1.65, -0.18]	71	0.71	0.5448	4.69 ± 2.76	6.62 ± 5.13	-0.43 [-1.30, 0.41]	58	0.59	0.1288	3.27 ± 2.42	6.08 ± 4.36	-0.73 [-1.60, 0.12]	53	0.72
histogram - mean	0.1137	2.43 ± 0.96	1.79 ± 0.73	0.74 [-0.21, 1.93]	50	0.72	0.5120	2.21 ± 1.08	1.78 ± 0.77	0.45 [-0.60, 1.60]	50	0.59	0.2318	2.16 ± 0.89	1.73 ± 0.69	0.53 [-0.42, 1.79]	50	0.67
histogram - variance	0.1770	1.07 ± 0.46	0.81 ± 0.51	0.51 [-0.57, 1.52]	45	0.69	0.7871	0.84 ± 0.44	0.85 ± 0.44	-0.01 [-1.06, 0.97]	60	0.54	0.2976	1.05 ± 0.38	0.81 ± 0.57	0.45 [-0.48, 1.26]	60	0.65
histogram - skewness	0.0171	0.48 ± 0.83	2.59 ± 3.16	-0.77 [-1.30, -0.14]	37	0.83	0.3749	1.25 ± 1.76	3.02 ± 4.55	-0.46 [-1.00, 0.49]	50	0.63	0.3348	1.19 ± 1.45	5.13 ± 11.09	-0.43 [-0.80, 0.70]	60	0.64
histogram - kurtosis	0.1673	0.28 ± 1.07	16.87 ± 38.82	-0.51 [-0.72, 0.07]	37	0.70	0.7871	4.04 ± 5.92	27.78 ± 77.71	-0.37 [-0.94, 0.61]	60	0.54	0.6160	2.60 ± 6.18	139.09 ± 456.60	-0.37 [-0.67, 0.65]	60	0.57

Feature	Measurement 1						Measurement 2						Measurement 3					
	p	pCR	non-pCR	Effect size	Acc (%)	AUC	p	pCR	non-pCR	Effect size	Acc (%)	AUC	p	pCR	non-pCR	Effect size	Acc (%)	AUC
histogram - median	0.1833	2.25 ± 1.16	1.58 ± 0.90	0.63 [-0.42, 1.67]	45	0.67	0.2761	2.13 ± 1.46	1.50 ± 1.00	0.50 [-0.46, 1.60]	50	0.63	0.5546	1.88 ± 1.25	1.42 ± 0.67	0.47 [-0.57, 1.85]	55	0.57
histogram - interquartile range	0.1256	1.38 ± 0.74	0.83 ± 0.72	0.71 [-0.39, 1.71]	60	0.70	0.6438	1.00 ± 0.76	0.83 ± 0.72	0.22 [-0.62, 1.26]	60	0.56	0.5938	1.13 ± 0.64	0.92 ± 0.90	0.25 [-0.56, 1.20]	60	0.57
histogram - range	0.6677	4.75 ± 1.98	4.75 ± 1.36	0.00 [-1.23, 0.87]	60	0.56	0.4140	4.88 ± 0.83	4.50 ± 1.24	0.33 [-0.71, 1.01]	60	0.60	0.8047	4.88 ± 0.64	4.58 ± 1.38	0.24 [-0.62, 1.07]	60	0.54
fractal dimension	0.2698	1.30 ± 0.19	1.16 ± 0.18	0.69 [-0.35, 1.75]	29	0.67	0.9039	1.05 ± 0.40	1.08 ± 0.20	-0.08 [-1.45, 0.99]	58	0.52	0.4920	1.10 ± 0.26	1.00 ± 0.34	0.29 [-0.59, 1.17]	58	0.60
fractal R^2	0.6009	1.00 ± 0.00	0.99 ± 0.00	0.32 [-0.70, 1.17]	29	0.59	0.7780	0.93 ± 0.18	0.98 ± 0.02	-0.46 [-2.11, 0.64]	58	0.55	0.9039	0.99 ± 0.01	0.95 ± 0.08	0.48 [-0.65, 1.27]	58	0.52

Supplementary Table 21: Summary statistics of features extracted from contrast-mode MIPs for changes between measurements. Delta radiomic features of changes include relative change, absolute change, minimum, and maximum of the two measurements in question. From left to right: feature name, p-value for a statistical significance between patients with pCR and non-pCR (significant features are highlighted in light blue, significant features after Benjamini-Hochberg correction with a false discovery rate of 5% or 10% are highlighted in dark or medium blue, respectively), mean ± standard deviation of patients with pCR, mean ± standard deviation of patients with non-pCR, effect size expressed by Hedges' g with bootstrapped 95% confidence interval, cross-validated predictive accuracy, area under the receiver operating characteristic curve (AUC).

	Measurement 1 → 2						Measurement 2 → 3						Measurement 1 → 3					
**Feature**	**p**	**pCR**	**non-pCR**	**Effect size**	**Acc (%)**	**AUC**	**p**	**pCR**	**non-pCR**	**Effect size**	**Acc (%)**	**AUC**	**p**	**pCR**	**non-pCR**	**Effect size**	**Acc (%)**	**AUC**
relative change (ΔF)																		
coverage (%)	0.2976	-3.45 ± 35.87	1.14 ± 12.80	-0.18 [-1.30, 1.66]	60	0.65	1.0000	-0.77 ± 31.64	-1.50 ± 18.83	0.03 [-1.47, 1.03]	60	0.59	0.5120	-4.22 ± 19.93	-0.36 ± 15.89	-0.21 [-1.19, 1.12]	60	0.50
distances – mean (mm)	0.5046	1.04 ± 1.69	-0.08 ± 1.02	0.80 [-0.69, 1.89]	59	0.64	0.8471	-0.64 ± 1.72	-0.10 ± 1.33	-0.34 [-1.33, 0.65]	60	0.59	0.7868	0.24 ± 1.34	-0.18 ± 1.69	0.26 [-0.69, 1.29]	60	0.54
distances – variance (mm^2)	0.9623	1.14 ± 2.07	-0.34 ± 4.01	0.42 [-0.69, 1.06]	53	0.51	0.5726	-0.70 ± 2.16	-1.02 ± 1.71	0.16 [-0.83, 1.33]	44	0.63	0.4079	0.33 ± 2.20	-1.48 ± 4.81	0.44 [-0.58, 1.26]	56	0.59
distances – skewness	0.5362	-0.44 ± 1.05	-0.06 ± 0.40	-0.50 [-1.99, 1.17]	59	0.60	0.8968	-0.15 ± 1.52	0.01 ± 0.52	-0.14 [-1.49, 1.01]	56	0.59	0.6065	-0.13 ± 0.88	-0.04 ± 0.82	-0.10 [-1.15, 0.97]	56	0.53
distances – kurtosis	0.6009	-0.76 ± 2.78	-0.07 ± 1.11	-0.33 [-1.53, 0.99]	47	0.59	0.8968	-1.68 ± 6.24	0.37 ± 1.20	-0.46 [-1.78, 0.63]	50	0.56	0.7577	-0.32 ± 2.37	0.25 ± 2.28	-0.24 [-1.25, 0.81]	50	0.53
distances – median (mm)	0.3604	1.13 ± 1.71	-0.11 ± 1.01	0.89 [-0.24, 2.19]	59	0.69	0.7285	-0.69 ± 1.70	0.02 ± 1.49	-0.42 [-1.43, 0.50]	60	0.62	0.6709	0.27 ± 1.17	-0.12 ± 1.78	0.24 [-0.77, 1.32]	60	0.56
distances – interquartile range (mm)	0.6009	0.84 ± 1.79	-0.11 ± 0.85	0.69 [-0.46, 2.15]	47	0.59	0.9654	-0.46 ± 1.64	-0.34 ± 1.20	-0.08 [-1.09, 1.11]	56	0.60	0.5360	0.24 ± 1.77	-0.44 ± 1.61	0.39 [-0.63, 1.44]	56	0.51
distances – range (mm)	0.6009	1.87 ± 3.53	-0.32 ± 2.86	0.66 [-0.37, 1.71]	29	0.59	0.9654	-1.42 ± 3.42	-0.66 ± 2.41	-0.25 [-1.29, 0.77]	56	0.59	0.6065	0.27 ± 3.59	-0.89 ± 3.44	0.31 [-0.94, 1.27]	56	0.51
histogram - mean	0.5628	-0.22 ± 1.91	-0.01 ± 0.59	-0.16 [-1.46, 1.25]	60	0.58	0.8471	-0.05 ± 1.56	-0.05 ± 1.24	0.00 [-0.94, 0.94]	60	0.55	0.7285	-0.28 ± 1.27	-0.06 ± 1.17	-0.17 [-1.08, 0.94]	60	0.53
histogram - variance	0.1137	-0.23 ± 0.84	0.04 ± 0.50	-0.40 [-1.67, 0.85]	50	0.72	0.5120	0.21 ± 0.66	-0.04 ± 0.82	0.31 [-0.64, 1.26]	60	0.52	0.9079	-0.03 ± 0.61	0.00 ± 0.84	-0.03 [-0.89, 0.90]	60	0.59
histogram - skewness	0.5918	1.10 ± 2.15	0.43 ± 2.37	0.28 [-0.64, 1.35]	53	0.58	0.7285	-0.06 ± 2.48	2.11 ± 13.06	-0.20 [-1.02, 0.62]	60	0.56	0.7108	0.67 ± 1.96	2.54 ± 12.36	-0.18 [-0.87, 0.79]	58	0.55

Feature	Measurement 1 → 2						Measurement 2 → 3						Measurement 1 → 3					
	p	pCR	non-pCR	Effect size	Acc (%)	AUC	p	pCR	non-pCR	Effect size	Acc (%)	AUC	p	pCR	non-pCR	Effect size	Acc (%)	AUC
histogram - kurtosis	0.5358	4.22 ± 6.75	10.91 ± 42.65	-0.18 [-0.97, 0.71]	58	0.60	0.9692	-1.44 ± 8.87	111.31 ± 472.12	-0.29 [-0.98, 0.57]	55	0.60	0.5358	2.57 ± 6.98	122.22 ± 463.87	-0.31 [-0.95, 0.57]	53	0.51
histogram - median	0.7454	-0.13 ± 2.36	-0.08 ± 0.79	-0.03 [-1.37, 1.40]	60	0.55	1.0000	-0.25 ± 2.05	-0.08 ± 1.31	-0.10 [-1.29, 0.89]	60	0.57	0.6270	-0.38 ± 1.77	-0.17 ± 1.27	-0.13 [-1.25, 0.87]	60	0.51
histogram - interquartile range	0.5805	-0.38 ± 1.19	0.00 ± 0.60	-0.41 [-1.70, 0.74]	50	0.57	0.7759	0.13 ± 1.25	0.08 ± 1.16	0.03 [-1.11, 1.02]	60	0.61	0.4127	-0.25 ± 0.71	0.08 ± 1.08	-0.33 [-1.27, 0.54]	60	0.54
histogram - range	1.0000	0.13 ± 2.59	-0.25 ± 1.06	0.20 [-0.82, 1.81]	60	0.50	1.0000	0.00 ± 1.20	0.08 ± 2.15	-0.04 [-0.92, 0.74]	60	0.52	0.9025	0.13 ± 1.73	-0.17 ± 1.95	0.15 [-0.67, 1.13]	60	0.50
fractal dimension	0.3638	-0.32 ± 0.52	-0.06 ± 0.23	-0.66 [-1.65, 0.44]	41	0.64	0.8286	0.04 ± 0.59	-0.08 ± 0.32	0.25 [-0.87, 1.14]	50	0.51	1.0000	-0.13 ± 0.31	-0.15 ± 0.41	0.03 [-1.07, 1.05]	56	0.54
fractal R^2	0.2295	-0.08 ± 0.19	-0.01 ± 0.02	-0.56 [-2.92, 0.36]	47	0.69	0.8286	0.06 ± 0.18	-0.03 ± 0.08	0.63 [-0.15, 1.47]	61	0.63	0.4079	-0.01 ± 0.01	-0.02 ± 0.07	0.32 [-1.07, 1.51]	50	0.54
absolute change (|ΔF|)																		
coverage (%)	0.1770	22.96 ± 26.41	7.51 ± 10.19	0.81 [-0.28, 1.92]	60	0.69	0.3749	20.02 ± 23.32	10.35 ± 15.50	0.49 [-0.48, 1.44]	50	0.67	0.2318	15.68 ± 11.69	9.57 ± 12.36	0.48 [-0.49, 1.64]	50	0.63
distances – mean (mm)	0.0947	1.55 ± 1.16	0.66 ± 0.75	0.91 [-0.34, 2.01]	65	0.77	0.9692	1.31 ± 1.21	0.96 ± 0.87	0.32 [-0.70, 1.41]	60	0.59	0.3943	1.00 ± 0.83	1.33 ± 0.94	-0.35 [-1.38, 0.77]	60	0.61
distances – variance (mm^2)	0.6009	1.65 ± 1.63	1.96 ± 3.46	-0.10 [-0.95, 1.11]	53	0.59	0.5148	1.67 ± 1.42	1.46 ± 1.30	0.15 [-0.90, 1.09]	56	0.67	0.2991	1.44 ± 1.60	3.01 ± 3.92	-0.47 [-1.21, 0.54]	56	0.60
distances – skewness	0.0020	0.96 ± 0.50	0.26 ± 0.29	1.71 [0.41, 3.81]	71	0.93	0.0831	1.03 ± 1.06	0.39 ± 0.32	0.83 [-0.46, 1.67]	67	0.68	0.2523	0.76 ± 0.34	0.58 ± 0.55	0.37 [-0.87, 1.58]	72	0.75
distances – kurtosis	0.0031	2.42 ± 1.26	0.72 ± 0.81	1.58 [0.40, 3.35]	65	0.91	0.0831	3.47 ± 5.33	0.84 ± 0.89	0.70 [-0.54, 1.27]	78	0.76	0.0907	2.08 ± 0.82	1.36 ± 1.78	0.47 [-0.77, 1.80]	78	0.75
distances – median (mm)	0.1342	1.59 ± 1.20	0.66 ± 0.74	0.93 [-0.20, 2.27]	71	0.74	1.0000	1.28 ± 1.25	1.04 ± 1.02	0.21 [-0.73, 1.18]	60	0.63	0.2783	0.94 ± 0.64	1.39 ± 1.01	-0.49 [-1.47, 0.63]	69	0.60
distances – interquartile range (mm)	0.0553	1.52 ± 1.15	0.56 ± 0.62	1.05 [-0.09, 2.40]	65	0.79	0.3154	1.35 ± 0.91	0.92 ± 0.80	0.49 [-0.57, 1.40]	44	0.56	0.7577	1.26 ± 1.16	1.33 ± 0.90	-0.07 [-1.02, 1.24]	50	0.65

	Measurement 1 → 2						Measurement 2 → 3						Measurement 1 → 3					
**Feature**	**p**	**pCR**	**non-pCR**	**Effect size**	**Acc (%)**	**AUC**	**p**	**pCR**	**non-pCR**	**Effect size**	**Acc (%)**	**AUC**	**p**	**pCR**	**non-pCR**	**Effect size**	**Acc (%)**	**AUC**
distances – range (mm)	0.5362	2.90 ± 2.60	1.87 ± 2.09	0.42 [-0.81, 1.63]	47	0.60	0.4598	2.66 ± 2.43	1.80 ± 1.65	0.41 [-0.78, 1.38]	44	0.52	0.9182	2.62 ± 2.23	2.47 ± 2.43	0.06 [-0.98, 1.18]	44	0.61
histogram - mean	0.0151	1.51 ± 1.05	0.46 ± 0.35	1.42 [0.16, 2.44]	80	0.83	0.2030	1.19 ± 0.90	0.76 ± 0.95	0.44 [-0.51, 1.56]	50	0.60	0.4636	1.05 ± 0.67	0.81 ± 0.80	0.30 [-0.52, 1.36]	50	0.68
histogram - variance	0.2318	0.65 ± 0.54	0.37 ± 0.31	0.65 [-0.37, 1.88]	55	0.67	0.6713	0.48 ± 0.47	0.59 ± 0.55	-0.20 [-0.96, 0.93]	60	0.51	0.9692	0.49 ± 0.31	0.62 ± 0.54	-0.27 [-1.01, 0.57]	60	0.56
histogram - skewness	0.2268	1.97 ± 1.22	1.62 ± 1.72	0.21 [-0.65, 1.31]	58	0.68	0.6713	1.94 ± 1.36	5.84 ± 11.76	-0.40 [-1.14, 0.62]	60	0.62	0.4320	1.42 ± 1.41	5.59 ± 11.22	-0.44 [-0.84, 0.47]	60	0.56
histogram - kurtosis	1.0000	5.47 ± 5.61	19.25 ± 39.29	-0.41 [-0.93, 0.60]	58	0.50	0.3749	5.67 ± 6.65	162.96 ± 455.46	-0.42 [-0.72, 0.62]	68	0.79	0.0449	3.51 ± 6.48	152.24 ± 454.07	-0.39 [-0.59, 0.55]	55	0.63
histogram - median	0.0069	1.88 ± 1.25	0.42 ± 0.67	1.49 [-0.08, 2.64]	80	0.85	0.1765	1.50 ± 1.31	0.75 ± 1.06	0.62 [-0.46, 1.59]	45	0.65	0.2688	1.38 ± 1.06	0.83 ± 0.94	0.53 [-0.70, 1.44]	45	0.68
histogram - interquartile range	0.1292	0.88 ± 0.83	0.33 ± 0.49	0.80 [-0.19, 2.00]	45	0.69	0.7405	0.88 ± 0.83	0.75 ± 0.87	0.14 [-0.77, 1.18]	60	0.58	0.5225	0.50 ± 0.53	0.75 ± 0.75	-0.35 [-1.24, 0.49]	60	0.55
histogram - range	0.9670	1.38 ± 2.13	0.75 ± 0.75	0.41 [-0.68, 1.75]	50	0.51	0.7710	0.75 ± 0.89	1.25 ± 1.71	-0.33 [-0.99, 0.82]	60	0.54	0.7651	1.13 ± 1.25	1.33 ± 1.37	-0.15 [-0.91, 0.97]	60	0.54
fractal dimension	0.1932	0.42 ± 0.44	0.18 ± 0.14	0.74 [-0.32, 1.65]	35	0.70	0.4082	0.41 ± 0.40	0.22 ± 0.23	0.56 [-0.56, 1.55]	56	0.52	0.9182	0.26 ± 0.19	0.31 ± 0.28	-0.19 [-1.24, 0.99]	56	0.63
fractal R^2	0.2698	0.08 ± 0.19	0.01 ± 0.02	0.55 [-0.45, 2.41]	47	0.67	0.8286	0.07 ± 0.17	0.04 ± 0.07	0.23 [-0.71, 1.66]	56	0.65	0.3510	0.01 ± 0.01	0.03 ± 0.06	-0.35 [-1.73, 1.06]	56	0.54
minimum (F_{min})																		
coverage (%)	0.7281	3.91 ± 5.05	3.53 ± 4.96	0.07 [-0.73, 1.17]	60	0.55	0.2974	3.27 ± 3.09	1.93 ± 2.08	0.51 [-0.45, 1.49]	70	0.76	0.0631	7.17 ± 9.43	1.75 ± 2.23	0.85 [-0.30, 1.65]	75	0.65
distances – mean (mm)	0.0186	0.51 ± 0.49	1.75 ± 1.42	-1.05 [-1.79, -0.28]	74	0.81	0.2318	0.67 ± 0.72	1.42 ± 1.46	-0.59 [-1.18, 0.39]	60	0.79	0.0339	0.46 ± 0.34	1.39 ± 1.28	-0.87 [-1.44, -0.23]	65	0.67
distances – variance (mm^2)	0.0754	0.51 ± 0.93	3.30 ± 4.08	-0.84 [-1.44, -0.18]	58	0.75	0.2633	0.54 ± 0.80	2.68 ± 4.74	-0.55 [-1.00, 0.28]	60	0.76	0.0587	0.25 ± 0.28	2.31 ± 3.63	-0.69 [-1.17, -0.33]	65	0.66

Feature	Measurement 1 $\rightarrow$ 2						Measurement 2 $\rightarrow$ 3						Measurement 1 $\rightarrow$ 3					
	p	pCR	non-pCR	Effect size	Acc (%)	AUC	p	pCR	non-pCR	Effect size	Acc (%)	AUC	p	pCR	non-pCR	Effect size	Acc (%)	AUC
distances – skewness	0.4421	0.87 ± 1.27	0.71 ± 0.45	0.17 [-1.09, 1.72]	58	0.61	0.5628	0.50 ± 0.42	0.74 ± 0.70	-0.39 [-1.16, 0.57]	60	0.53	0.8471	0.66 ± 0.33	0.70 ± 0.63	-0.07 [-0.87, 0.81]	60	0.58
distances – kurtosis	0.7168	1.33 ± 5.89	-0.24 ± 1.03	0.39 [-0.82, 2.89]	58	0.56	0.6160	-0.52 ± 0.78	0.21 ± 1.80	-0.47 [-1.23, 0.44]	60	0.52	0.9079	-0.40 ± 0.68	0.07 ± 1.61	-0.33 [-0.99, 0.48]	60	0.57
distances – median (mm)	0.0150	0.31 ± 0.31	1.39 ± 1.21	-1.08 [-1.83, -0.45]	74	0.82	0.2023	0.52 ± 0.64	1.08 ± 1.13	-0.55 [-1.12, 0.55]	60	0.79	0.0372	0.29 ± 0.24	1.07 ± 1.08	-0.87 [-1.39, -0.28]	70	0.68
distances – interquartile range (mm)	0.0203	0.66 ± 0.73	2.21 ± 1.95	-0.95 [-1.65, -0.18]	68	0.82	0.1770	0.79 ± 0.88	1.71 ± 1.80	-0.59 [-1.18, 0.33]	60	0.77	0.0491	0.58 ± 0.40	1.66 ± 1.65	-0.79 [-1.38, -0.33]	65	0.69
distances – range (mm)	0.1518	2.61 ± 1.86	5.92 ± 4.55	-0.86 [-1.59, 0.05]	37	0.70	0.2976	2.65 ± 1.89	5.15 ± 4.64	-0.63 [-1.32, 0.17]	65	0.78	0.0409	2.01 ± 1.06	5.21 ± 4.19	-0.92 [-1.48, -0.37]	70	0.65
histogram - mean	0.9692	1.56 ± 0.57	1.56 ± 0.56	0.01 [-0.80, 1.21]	60	0.51	0.2976	1.59 ± 0.52	1.38 ± 0.32	0.50 [-0.44, 1.55]	60	0.70	0.1535	1.77 ± 0.67	1.35 ± 0.35	0.79 [-0.25, 1.87]	60	0.65
histogram - variance	1.0000	0.63 ± 0.39	0.64 ± 0.46	-0.03 [-0.92, 0.92]	60	0.50	0.2318	0.70 ± 0.31	0.54 ± 0.44	0.41 [-0.46, 1.36]	60	0.71	0.1325	0.82 ± 0.40	0.50 ± 0.43	0.72 [-0.29, 1.79]	60	0.67
histogram - skewness	0.0062	-0.09 ± 0.52	1.99 ± 3.00	-0.84 [-1.29, -0.25]	65	0.88	0.0698	0.25 ± 1.03	1.15 ± 1.15	-0.78 [-1.58, 0.26]	70	0.77	0.0491	0.28 ± 0.78	1.06 ± 1.20	-0.71 [-1.46, 0.16]	70	0.75
histogram - kurtosis	0.1770	-0.20 ± 0.53	12.70 ± 38.52	-0.41 [-0.61, 0.30]	45	0.69	0.7285	0.48 ± 1.77	1.96 ± 4.12	-0.42 [-1.09, 0.52]	60	0.52	0.9079	-0.06 ± 0.84	1.86 ± 4.10	-0.57 [-1.21, 0.12]	60	0.55
histogram - median	0.8517	1.25 ± 0.71	1.33 ± 0.78	-0.11 [-0.75, 1.09]	60	0.52	0.7671	1.25 ± 0.71	1.08 ± 0.29	0.32 [-0.58, 1.43]	55	0.59	0.3203	1.38 ± 0.74	1.08 ± 0.29	0.54 [-0.44, 1.55]	55	0.53
histogram - interquartile range	0.8305	0.75 ± 0.71	0.67 ± 0.65	0.12 [-0.77, 1.19]	60	0.53	0.6228	0.63 ± 0.52	0.50 ± 0.52	0.23 [-0.85, 1.02]	60	0.69	0.1416	1.00 ± 0.76	0.50 ± 0.67	0.68 [-0.52, 1.69]	60	0.56
histogram - range	0.8865	4.13 ± 1.81	4.25 ± 1.36	-0.08 [-1.26, 0.74]	60	0.52	0.3980	4.50 ± 0.76	3.92 ± 1.44	0.46 [-0.39, 1.04]	60	0.58	0.5524	4.25 ± 1.75	4.00 ± 1.54	0.15 [-1.14, 0.98]	60	0.61
fractal dimension	1.0000	1.01 ± 0.39	1.02 ± 0.16	-0.04 [-1.39, 0.98]	58	0.50	0.5120	0.87 ± 0.32	0.98 ± 0.32	-0.32 [-1.29, 0.52]	60	0.57	0.6160	1.04 ± 0.21	0.96 ± 0.29	0.29 [-0.55, 1.19]	60	0.59
fractal R^2	0.9678	0.92 ± 0.18	0.98 ± 0.02	-0.45 [-3.30, 0.56]	58	0.51	0.2976	0.92 ± 0.17	0.95 ± 0.08	-0.25 [-2.00, 0.65]	60	0.57	0.6160	0.98 ± 0.01	0.96 ± 0.08	0.44 [-1.03, 1.09]	60	0.65

	Measurement 1 → 2						Measurement 2 → 3						Measurement 1 → 3					
**Feature**	**p**	**pCR**	**non-pCR**	**Effect size**	**Acc (%)**	**AUC**	**p**	**pCR**	**non-pCR**	**Effect size**	**Acc (%)**	**AUC**	**p**	**pCR**	**non-pCR**	**Effect size**	**Acc (%)**	**AUC**
maximum (F_{max})																		
coverage (%)	0.0228	26.87 ± 23.72	11.04 ± 14.29	0.82 [-0.10, 1.77]	50	0.81	0.3749	23.29 ± 23.84	12.28 ± 14.67	0.56 [-0.36, 1.65]	50	0.75	0.0698	22.84 ± 17.00	11.32 ± 11.80	0.79 [-0.32, 1.87]	55	0.63
distances – mean (mm)	1.0000	2.12 ± 1.11	2.48 ± 2.04	-0.20 [-1.19, 0.83]	60	0.53	0.1768	1.98 ± 1.25	2.64 ± 1.65	-0.42 [-1.24, 0.56]	60	0.68	0.1880	1.44 ± 0.76	2.95 ± 1.99	-0.90 [-1.67, 0.15]	75	0.63
distances – variance (mm^2)	0.9039	1.95 ± 1.58	5.08 ± 7.03	-0.55 [-1.26, 0.34]	58	0.52	0.9079	2.21 ± 1.83	0.00 ± 0.00	-0.39 [-1.04, 0.41]	60	0.60	0.4636	1.51 ± 1.49	4.57 ± 6.64	-0.55 [-1.15, 0.21]	60	0.52
distances – skewness	0.0620	1.71 ± 0.94	0.94 ± 0.57	0.97 [-0.04, 1.60]	37	0.76	0.2976	1.53 ± 1.04	1.07 ± 0.60	0.55 [-0.29, 1.57]	60	0.58	0.5628	1.33 ± 0.42	1.14 ± 0.66	0.32 [-0.61, 1.14]	60	0.65
distances – kurtosis	0.0409	3.44 ± 5.14	0.42 ± 1.38	0.83 [-0.11, 1.54]	37	0.78	0.3348	2.94 ± 5.37	0.91 ± 2.03	0.53 [-0.33, 1.59]	60	0.61	0.4179	1.42 ± 1.26	1.09 ± 1.97	0.19 [-0.74, 1.18]	60	0.64
distances – median (mm)	0.9077	1.95 ± 1.17	2.09 ± 1.78	-0.08 [-0.93, 0.96]	60	0.56	0.1768	1.81 ± 1.25	2.32 ± 1.51	-0.35 [-1.29, 0.65]	60	0.71	0.1412	1.19 ± 0.65	2.61 ± 1.87	-0.90 [-1.69, -0.07]	75	0.63
distances – interquartile range (mm)	1.0000	1.99 ± 1.17	2.72 ± 2.43	-0.35 [-1.22, 0.56]	58	0.50	0.9079	2.14 ± 1.29	2.48 ± 2.17	-0.17 [-0.89, 0.82]	60	0.56	0.6713	1.68 ± 1.07	2.66 ± 2.44	-0.46 [-1.13, 0.43]	60	0.52
distances – range (mm)	0.6574	5.15 ± 2.32	7.62 ± 5.77	-0.51 [-1.33, 0.26]	58	0.57	0.7871	5.31 ± 2.65	6.65 ± 4.85	-0.31 [-1.10, 0.49]	60	0.63	0.3749	4.30 ± 2.29	7.07 ± 5.30	-0.61 [-1.28, 0.20]	60	0.54
histogram - mean	0.0151	3.08 ± 0.70	2.02 ± 0.84	1.29 [0.23, 2.42]	75	0.83	0.1535	2.78 ± 0.94	2.14 ± 0.81	0.71 [-0.23, 2.04]	65	0.77	0.0491	2.82 ± 0.83	2.17 ± 0.73	0.81 [-0.35, 1.91]	65	0.70
histogram - variance	0.0698	1.28 ± 0.20	1.01 ± 0.42	0.74 [-0.26, 1.38]	40	0.75	0.7871	1.18 ± 0.37	1.12 ± 0.39	0.16 [-0.64, 1.33]	60	0.65	0.2976	1.31 ± 0.23	1.12 ± 0.44	0.47 [-0.42, 1.24]	60	0.54
histogram - skewness	0.4636	1.63 ± 1.59	3.61 ± 4.51	-0.52 [-1.01, 0.24]	60	0.60	0.6160	2.19 ± 1.41	6.99 ± 11.23	-0.52 [-0.93, 0.39]	60	0.82	0.0186	1.52 ± 1.23	6.65 ± 10.86	-0.58 [-0.85, 0.04]	65	0.57
histogram - kurtosis	0.6713	4.59 ± 5.53	31.96 ± 76.97	-0.43 [-0.78, 0.53]	60	0.56	0.4179	6.16 ± 7.28	164.92 ± 454.73	-0.43 [-0.72, 0.48]	70	0.80	0.0279	3.01 ± 6.01	154.11 ± 453.30	-0.41 [-0.62, 0.74]	70	0.61
histogram - median	0.0128	3.13 ± 0.99	1.75 ± 1.06	1.28 [0.23, 2.36]	75	0.83	0.1354	2.75 ± 1.39	1.83 ± 1.03	0.74 [-0.28, 1.80]	50	0.72	0.0938	2.75 ± 1.16	1.92 ± 0.90	0.79 [-0.19, 2.06]	45	0.70
histogram - interquartile range	0.0646	1.63 ± 0.52	1.00 ± 0.74	0.90 [0.05, 1.95]	70	0.73	0.6415	1.50 ± 0.53	1.25 ± 0.87	0.32 [-0.49, 1.19]	60	0.58	0.5225	1.50 ± 0.53	1.25 ± 0.75	0.35 [-0.48, 1.19]	60	0.56

Feature	Measurement 1 → 2						Measurement 2 → 3						Measurement 1 → 3					
	p	pCR	non-pCR	Effect size	Acc (%)	AUC	p	pCR	non-pCR	Effect size	Acc (%)	AUC	p	pCR	non-pCR	Effect size	Acc (%)	AUC
histogram - range	0.3250	5.50 ± 0.53	5.00 ± 1.13	0.51 [-0.21, 1.27]	40	0.63	0.8942	5.25 ± 0.46	5.17 ± 0.72	0.13 [-0.83, 0.93]	60	0.53	0.8646	5.38 ± 0.74	5.33 ± 0.65	0.06 [-1.00, 0.94]	60	0.00
fractal dimension	0.0506	1.38 ± 0.16	1.19 ± 0.20	0.97 [0.16, 1.77]	63	0.77	0.4179	1.28 ± 0.18	1.16 ± 0.21	0.55 [-0.28, 1.41]	60	0.67	0.2318	1.27 ± 0.30	1.19 ± 0.25	0.27 [-0.98, 1.18]	55	0.11
fractal R^2	0.9678	1.00 ± 0.00	0.99 ± 0.02	0.39 [-0.65, 1.01]	58	0.51	0.9079	0.99 ± 0.00	0.99 ± 0.02	0.31 [-0.53, 1.14]	60	0.54	0.7871	0.99 ± 0.01	0.98 ± 0.06	0.32 [-0.71, 1.06]	1	0.00

Supplementary Table 22: Summary statistics of features extracted from contrast-mode MIPs for the three measurements combined. Delta radiomic features include the minimum and maximum of the three measurements. From left to right: feature name, p-value for a statistical significance between patients with pCR and non-pCR, mean ± standard deviation of patients with pCR, mean ± standard deviation of patients with non-pCR, effect size expressed by Hedges' g with bootstrapped 95% confidence interval, cross-validated predictive accuracy, area under the receiver operating characteristic curve (AUC).

Feature	Measurement 1 & 2 & 3					
	p	pCR	non-pCR	Effect size	Acc (%)	AUC
minimum (F_{min})						
coverage (%)	0.2454	2.36 ± 2.17	1.13 ± 1.19	0.72 [-0.33, 1.94]	60	0.66
distances – mean (mm)	0.0279	0.31 ± 0.23	1.31 ± 1.32	-0.92 [-1.41, -0.34]	75	0.80
distances – variance (mm^2)	0.0587	0.16 ± 0.18	2.27 ± 3.66	-0.71 [-1.22, -0.39]	75	0.76
distances – skewness	0.3749	0.35 ± 0.19	0.67 ± 0.64	-0.60 [-1.19, 0.35]	60	0.63
distances – kurtosis	0.5120	-0.85 ± 0.18	-0.03 ± 1.66	-0.61 [-1.19, 0.03]	60	0.59
distances – median (mm)	0.0276	0.18 ± 0.18	1.00 ± 1.09	-0.91 [-1.48, -0.43]	75	0.80
distances – interquartile range (mm)	0.0122	0.37 ± 0.25	1.62 ± 1.68	-0.91 [-1.40, -0.46]	75	0.84
distances – range (mm)	0.1137	1.86 ± 1.02	4.94 ± 4.41	-0.84 [-1.45, -0.30]	75	0.72
histogram - mean	0.4179	1.38 ± 0.27	1.28 ± 0.26	0.33 [-0.63, 1.36]	60	0.61
histogram - variance	0.3348	0.58 ± 0.37	0.42 ± 0.37	0.42 [-0.44, 1.38]	55	0.64
histogram - skewness	0.0049	-0.25 ± 0.39	0.93 ± 1.03	-1.35 [-2.04, -0.70]	80	0.89
histogram - kurtosis	0.6713	-0.38 ± 0.52	1.17 ± 2.70	-0.69 [-1.33, -0.06]	40	0.56
histogram - median	NaN	1.00 ± 0.00	1.00 ± 0.00	NaN [NaN, NaN]	60	NaN
histogram - interquartile range	0.7543	0.50 ± 0.53	0.42 ± 0.51	0.15 [-0.81, 1.12]	60	0.54
histogram - range	0.5604	4.00 ± 1.77	3.75 ± 1.54	0.15 [-1.07, 0.96]	60	0.58
fractal dimension	0.5628	0.85 ± 0.30	0.93 ± 0.28	-0.26 [-1.28, 0.58]	60	0.58
fractal R^2	0.3348	0.92 ± 0.17	0.95 ± 0.08	-0.25 [-1.61, 0.67]	60	0.64
maximum (F_{max})						
coverage (%)	0.0409	31.69 ± 22.08	14.85 ± 15.26	0.89 [-0.12, 2.00]	45	0.78
distances – mean (mm)	0.4858	2.33 ± 1.00	3.12 ± 2.00	-0.46 [-1.39, 0.54]	60	0.56
distances – variance (mm^2)	1.0000	2.35 ± 1.71	4.84 ± 6.76	-0.44 [-1.18, 0.40]	60	0.50
distances – skewness	0.0972	1.83 ± 0.85	1.16 ± 0.62	0.89 [0.21, 1.66]	45	0.73
distances – kurtosis	0.0698	3.88 ± 4.88	1.16 ± 1.98	0.76 [-0.08, 1.51]	50	0.75
distances – median (mm)	0.6422	2.17 ± 1.00	2.80 ± 1.84	-0.38 [-1.46, 0.65]	60	0.51
distances – interquartile range (mm)	0.7285	2.30 ± 1.13	2.75 ± 2.47	-0.21 [-0.98, 0.61]	60	0.55
distances – range (mm)	0.9079	5.62 ± 2.32	7.50 ± 5.54	-0.40 [-1.16, 0.52]	60	0.52
histogram - mean	0.0279	3.25 ± 0.62	2.30 ± 0.83	1.21 [0.19, 2.30]	65	0.80
histogram - variance	0.1535	1.39 ± 0.21	1.21 ± 0.34	0.59 [-0.46, 1.41]	45	0.70
histogram - skewness	0.1325	2.36 ± 1.21	7.46 ± 11.05	-0.56 [-0.91, 0.13]	35	0.71
histogram - kurtosis	0.1325	6.39 ± 7.09	168.39 ± 453.50	-0.44 [-0.66, 0.56]	35	0.71
histogram - median	0.0133	3.38 ± 0.92	2.00 ± 1.04	1.32 [0.35, 2.30]	55	0.83
histogram - interquartile range	0.4611	1.63 ± 0.52	1.33 ± 0.78	0.41 [-0.40, 1.28]	60	0.59
histogram - range	0.5386	5.63 ± 0.52	5.42 ± 0.67	0.32 [-0.58, 1.12]	60	0.58
fractal dimension	0.0491	1.41 ± 0.14	1.23 ± 0.20	0.95 [0.11, 1.65]	70	0.77
fractal R^2	0.9079	1.00 ± 0.00	0.99 ± 0.02	0.41 [-0.82, 0.86]	60	0.52

NaN: not any number

Supplementary Table 23: Summary statistics of features extracted from histological CD31 masks. From left to right: feature name, p-value for a statistical significance between patients with pCR and non-pCR (significant features are highlighted in light blue, significant features after Benjamini-Hochberg correction with a false discovery rate of 5% or 10% are highlighted in dark or medium blue, respectively), mean ± standard deviation of patients with pCR, mean ± standard deviation of patients with non-pCR, effect size expressed by Hedges' g with bootstrapped 95% confidence interval, cross-validated predictive accuracy, area under the receiver operating characteristic curve (AUC).

Feature	p	pCR	non-pCR	Effect size	Acc (%)	AUC
coverage (%)	0.0018	4.45 ± 3.50	1.21 ± 0.99	1.39 [0.30, 2.58]	47	0.92
distances – mean (µm)	0.0052	34.55 ± 14.86	77.12 ± 51.47	-0.96 [-1.50, -0.27]	58	0.88
distances – variance (µm^2)	0.0171	1050.30 ± 681.59	5941.18 ± 9347.56	-0.62 [-1.09, -0.37]	58	0.83
distances – skewness	0.6504	1.74 ± 0.58	1.94 ± 1.60	-0.14 [-0.85, 0.86]	58	0.57
distances – kurtosis	0.8369	4.55 ± 3.23	16.65 ± 44.21	-0.32 [-1.02, 0.82]	58	0.54
distances – median (µm)	0.0098	26.77 ± 13.87	62.39 ± 40.19	-1.02 [-1.61, -0.30]	58	0.86
distances – interquartile range (µm)	0.0072	35.46 ± 12.86	75.11 ± 60.55	-0.77 [-1.16, -0.32]	58	0.87
distances – range (µm)	0.0171	259.89 ± 94.50	510.19 ± 253.10	-1.13 [-1.78, -0.50]	58	0.83
fractal dimension	0.0052	1.35 ± 0.09	1.22 ± 0.09	1.43 [0.45, 2.50]	58	0.88
fractal R^2	0.1003	1.00 ± 0.00	1.00 ± 0.00	0.75 [-0.26, 1.57]	53	0.74

Supplementary Table 24: Features considered significant after Benjamini-Hochberg correction. Given for a false discovery rate of 5% (highlighted in blue) or 10%. From left to right: feature name, p-value for a statistical significance between patients with pCR and non-pCR, mean ± standard deviation of patients with pCR, mean ± standard deviation of patients with non-pCR, effect size expressed by Hedges' g with bootstrapped 95% confidence interval, cross-validated predictive accuracy, area under the receiver operating characteristic curve (AUC).

Feature	p	pCR	non-pCR	Effect size	Accuracy (%)	AUC
ULM – measurement 1 → 2						
compensated vessel coverage in % ($\|\Delta F\|$)	0.0005	28.39 ± 12.52	7.50 ± 6.34	2.21 [0.48, 3.43]	84	0.95
velocity range – skewness (F_{min})	0.0014	0.66 ± 0.11	1.14 ± 0.51	-1.13 [-1.67, 0.70]	85	0.94
tortuosity (SOAM) – mean in rad/mm (F_{min})	0.0018	6.07± 0.40	7.93 ± 1.85	-1.21 [-1.99, -0.29]	90	0.93
track length – median in mm (F_{max})	0.0018	0.57 ± 0.04	0.44 ± 0.09	1.49 [0.81, 2.19]	80	0.93
distances – skewness ($\|\Delta F\|$)	0.0018	1.41 ± 0.52	0.58 ± 0.54	1.49 [0.15, 2.88]	68	0.92
compensated vessel coverage in % (F_{max})	0.0023	41.71 ± 11.45	16.06 ± 14.57	1.83 [0.71, 3.42]	80	0.92
tortuosity (SOAM) – median in rad/mm (F_{min})	0.0049	3.92 ± 0.56	5.80 ± 2.09	-1.08 [-1.60, -0.33]	75	0.89
Histological CD31 masks						
vessel coverage in %	0.0018	4.45 ± 3.50	1.21 ± 0.99	1.39 [0.30, 2.58]	47	0.92
fractal dimension	0.0052	1.35 ± 0.09	1.22 ± 0.09	1.43 [0.45, 2.50]	63	0.88
distances – range in mm	0.0171	0.26 ± 0.09	0.51 ± 0.25	-1.13 [-1.78, -0.50]	58	0.83
Contrast-mode MIPs – measurement 1 → 2						
distances – skewness ($\|\Delta F\|$)	0.0020	0.96 ± 0.50	0.26 ± 0.29	1.71 [0.41, 3.81]	71	0.93
distances – kurtosis ($\|\Delta F\|$)	0.0031	2.42 ± 1.26	0.72 ± 0.81	1.58 [0.40, 3.35]	65	0.91
histogram – skewness (F_{min})	0.0062	-0.09 ± 0.52	1.99 ± 3.00	-0.84 [-1.29, -0.25]	65	0.88
histogram – median ($\|\Delta F\|$)	0.0069	1.88 ± 1.25	0.42 ± 0.67	1.49 [-0.08, 2.64]	80	0.85

Supplementary Table 25: Sensitivity analysis of feature selection based on B-mode size measurements. Values indicate the number of times that features were selected by LASSO across the 20 LOOCV folds.

Individual measurements

1		2		3	
tumour volume	18	tumour volume	20	tumour volume	19

Changes between measurements

1 → 2		2 → 3		1 → 3	
tumour volume (F_{max})	9	tumour volume (F_{min})	18	tumour volume (F_{min})	18
tumour volume (F_{min})	6	tumour volume (ΔF)	6	tumour volume (ΔF)	6
tumour volume ($\lvert\Delta F\rvert$)	4	tumour volume (F_{max})	3		
		tumour volume ($\lvert\Delta F\rvert$)	1		

Combined measurements

1 & 2		1 & 2 & 3	
tumour volume ($F^{(2)}$)	12	tumour volume ($F^{(3)}$)	14
tumour volume ($\lvert\Delta F\rvert$)	10	tumour volume ($\Delta F^{(1,3)}$)	2
tumour volume (F_{max})	4	tumour volume ($F_{max}{}^{(2,3)}$)	2
tumour volume (ΔF)	2	tumour volume ($F^{(2)}$)	1
		tumour volume ($\Delta F^{(1,2)}$)	1
		tumour volume ($\lvert\Delta F^{(1,2)}\rvert$)	1
		tumour volume ($\Delta F^{(2,3)}$)	1
		tumour volume ($F_{min}{}^{(2,3)}$)	1

Supplementary Table 26: Sensitivity analysis of feature selection based on B-mode ultrasound images. Values indicate the number of times that features were selected by LASSO across the 20 LOOCV folds.

Individual measurements

1		2		3	
		variance of histogram	6		
		• kurtosis of histogram	3		
		• median of histogram			
		• fractal dimension			
		• fractal R^2			
		• mean of histogram	1		
		• skewness of histogram			
		• range of histogram			

Changes between measurements

1 → 2		2 → 3		1 → 3	
variance of histogram (ΔF)	2	variance of histogram (ΔF)	15	kurtosis of histogram ($\lvert\Delta F\rvert$)	7
• median of histogram (ΔF)	1	median of histogram (ΔF)	13	IQR of histogram ($\lvert\Delta F\rvert$)	6
• fractal dimension (ΔF)		fractal R^2 (ΔF)	12	• mean of histogram ($\lvert\Delta F\rvert$)	5
• skewness of histogram ($\lvert\Delta F\rvert$)		variance of histogram ($\lvert\Delta F\rvert$)	9	• fractal R^2 ($\lvert\Delta F\rvert$)	
• variance of histogram (F_{min})		range of histogram (ΔF)	2	median of histogram ($\lvert\Delta F\rvert$)	3
• range of histogram (F_{min})		• median of histogram ($\lvert\Delta F\rvert$)	1	kurtosis of histogram (F_{max})	2
		• mean of histogram (F_{min})		• kurtosis of histogram (ΔF)	1
		• variance of histogram (F_{min})		• variance of histogram ($\lvert\Delta F\rvert$)	
				• fractal dimension ($\lvert\Delta F\rvert$)	
				• mean of histogram (F_{min})	
				• range of histogram (F_{min})	
				• range of histogram (F_{max})	
				• fractal dimension (F_{max})	

Combined measurements

1 & 2		1 & 2 & 3	
variance of histogram (ΔF)	9	variance of histogram ($\Delta F^{(2,3)}$)	17
variance of histogram ($F^{(2)}$)	6	median of histogram ($\Delta F^{(2,3)}$)	14
• range of histogram (ΔF)	3	fractal R^2 ($\Delta F^{(2,3)}$)	10
• fractal R^2 (ΔF)		IQR of histogram ($\lvert\Delta F^{(1,3)}\rvert$)	9
• range of histogram ($F^{(2)}$)	2	variance of histogram ($\lvert\Delta F^{(2,3)}\rvert$)	6
• fractal dimension (ΔF)		• variance of histogram ($F^{(2)}$)	4
median of histogram (ΔF)	1	• variance of histogram ($\Delta F^{(1,2)}$)	
		• range of histogram ($\Delta F^{(2,3)}$)	
		median of histogram ($\Delta F^{(1,2)}$)	3
		• range of histogram ($F^{(2)}$)	2
		• variance of histogram ($F_{min}^{(1,2)}$)	
		• kurtosis of histogram ($\lvert\Delta F^{(1,3)}\rvert$)	
		• fractal R^2 ($\Delta F^{(1,2)}$)	1
		• range of histogram ($F_{min}^{(1,2)}$)	
		• fractal dimension ($F_{max}^{(1,2)}$)	
		• mean of histogram ($\lvert\Delta F^{(1,3)}\rvert$)	
		• range of histogram ($F_{max}^{(1,3)}$)	
		• mean of histogram ($\Delta F^{(2,3)}$)	
		• fractal dimension ($\Delta F^{(2,3)}$)	

IQR: interquartile range

Supplementary Table 27: Sensitivity analysis of feature selection based on contrast-mode MIPs. Values indicate the number of times that features were selected by LASSO across the 20 LOOCV folds.

Individual measurements

1		2		3	
IQR of histogram	8	fractal R^2	3	range of distances	10
fractal dimension	6	• kurtosis of distances	2	fractal R^2	8
• range of distances	1	• range of distances		skewness of distances	3
• fractal R^2		• median of histogram		vessel coverage	2
		• IQR of distances	1	• median of histogram	1
		• variance of histogram		• IQR of histogram	

Changes between measurements

1 → 2		2 → 3		1 → 3	
skewness of distances ($\|\Delta F\|$)	19	skewness of histogram (F_{min})	14	vessel coverage (F_{min})	14
IQR of distances ($\|\Delta F\|$)	18	skewness of distances ($\|\Delta F\|$)	6	range of distances (F_{min})	10
• kurtosis of distances ($\|\Delta F\|$)	9	IQR of distances ($\|\Delta F\|$)	5	fractal R^2 ($\|\Delta F\|$)	6
• median of histogram ($\|\Delta F\|$)		kurtosis of distances ($\|\Delta F\|$)	2	median of histogram ($\|\Delta F\|$)	5
• variance of histogram (ΔF)	3	• variance of distances (ΔF)	1	• skewness of distances ($\|\Delta F\|$)	3
• IQR of histogram (F_{max})		• mean of histogram ($\|\Delta F\|$)		• mean distance (F_{min})	
• skewness of histogram ($\|\Delta F\|$)	2	• variance of histogram (F_{min})		kurtosis of distances ($\|\Delta F\|$)	2
• skewness of histogram (F_{min})		• skewness of distances (F_{max})		• range of distances (ΔF)	1
mean of histogram ($\|\Delta F\|$)	1			• fractal R^2 (ΔF)	
				• median distance (F_{min})	
				• IQR of histogram (F_{min})	
				• median distance (F_{max})	

Combined measurements

1 & 2		1 & 2 & 3	
skewness of distances ($\|\Delta F\|$)	19	skewness of distances ($\|\Delta F^{(1,2)}\|$)	19
IQR of distances ($\|\Delta F\|$)	16	skewness of histogram ($F_{min}^{(1,2,3)}$)	17
kurtosis of distances ($\|\Delta F\|$)	9	IQR of distances ($\|\Delta F^{(1,2)}\|$)	12
median of histogram ($\|\Delta F\|$)	8	kurtosis of distances ($\|\Delta F^{(1,2)}\|$)	11
IQR of histogram (F_{max})	7	• median of histogram ($\|\Delta F^{(1,2)}\|$)	6
variance of histogram (ΔF)	5	• skewness of histogram ($F_{max}^{(1,3)}$)	
skewness of histogram (F_{min})	3	range of distances ($F_{max}^{(1,3)}$)	5
IQR of histogram ($F^{(1)}$)	2	mean distance ($\|\Delta F^{(1,2)}\|$)	2
• median distance (ΔF)	1	• variance of histogram ($\Delta F^{(1,2)}$)	1
• mean distance ($\|\Delta F\|$)		• vessel coverage ($F_{min}^{(1,3)}$)	
• fractal dimension ($\|\Delta F\|$)		• median distance ($F_{max}^{(1,3)}$)	
		• skewness of histogram ($F_{min}^{(2,3)}$)	

IQR: interquartile range

Supplementary Table 28: Sensitivity analysis of feature selection based on histological CD31 masks. Values indicate the number of times that features were selected by LASSO across the 20 LOOCV folds.

fractal dimension	20
range of distances	19
vessel coverage	12
• skewness of distances<br>• fractal R^2	2

References

1 Zwanenburg A, Vallières M, Abdalah MA, Aerts HJWL, Andrearczyk V, Apte A, et al. The Image Biomarker Standardization Initiative: Standardized quantitative radiomics for high-throughput image-based phenotyping. *Radiology* 2020;**295**(2):328–38.

2 Costa A. Hausdorff (box-counting) fractal dimension. MATLAB Central File Exchange; 2025. URL: https://de.mathworks.com/matlabcentral/fileexchange/30329-hausdorff-box-counting-fractal-dimension. Accessed on 2025 November 19.

Study protocol

Translated from the original German document. The current study corresponds to study part B of the clinical study.

Project title

Motion-Model Ultrasound Localisation Microscopy (mULM) for Diagnosis and Treatment Monitoring in Breast Cancer

Study director and principal investigator:

Univ.-Prof. Dr. med. E. Stickeler
Department of Gynecology and Obstetrics
Uniklinik RWTH Aachen
Pauwelsstr. 30
52074 Aachen
Tel. 0241-80-88400
Fax 0241-80-82476
Email: estickeler@ukaachen.de

Location and date / Study protocol version 5

____

Prof. E. Stickeler, M.D.

Abstract

Ultrasound is routinely used in breast cancer screening and to assess the dignity of suspicious lesions, and is supplemented by contrast-enhanced ultrasound [1–4]. With support from a DFG grant (SCHM1171 / 4-1, KI1072 / 11-1), we have developed a high-resolution contrast-enhanced ultrasound method called Motion-Model Ultrasound Localisation Microscopy (mULM), which, through the use of microbubbles (MB) as an ultrasound contrast agent, in addition to other parameters, enables an accurate assessment of the vascular architecture in tumors, individual blood flow velocities and directions, as well as the quantification of relative blood volume and perfusion. mULM describes the algorithm that tracks the motion of individual microbubbles using a probabilistic approach. The method is based on RaDAR measurement techniques. Markov Chain Monte Carlo Data Association enables the reliable and valid tracking of multiple objects. In general, the algorithms assign the most probable path to individual objects with measured positions. This assignment is a combinatorial problem in which the probability of a specific trajectory can be calculated. However, since it is not practical to compute all combinations due to limited computational resources, Markov Chain Monte Carlo Data Association has proven to be a good compromise between accuracy and computational complexity. In this approach, paths are generated randomly, with their trajectories being probability-weighted according to the Markov process [5].

We recently demonstrated in a mouse model that this method can be used to extract various morphometric parameters which, in combination with radiomic analysis, enable the automated differentiation of various tumor models [6–8].

In this study, we aim to adapt mULM and ultrasound-based radiomics for clinical application and extend the technique to 3D imaging. However, several technical challenges arose for robust application in a clinical setting: In particular, patient movement and lower image resolution, combined with increased slice thickness and non-optimized injection protocols, limited the use of only short image sequences and the extraction of a reduced number of MB paths.

As a first step, therefore, the mULM methodology will be refined and adapted for use in breast imaging with clinical ultrasound devices (study part A). Subsequently, the 2D-mULM method will be applied to monitor the response of breast cancer to neoadjuvant chemotherapy (study part B). Accuracy will be compared with conventional contrast-enhanced ultrasound data analyses. Furthermore, we aim to further develop the method from 2D to full 3D imaging and compare both approaches in a small cohort of patients with breast tumors of varying malignancy (study part C). In all three study parts, histological analyses of the tumor tissue will also be performed to compare them with the results of the mULM examinations and thus validate the method.

To fully harness the potential of mULM data analysis, algorithms are being developed and implemented to enable the automated extraction and cluster analysis of vascular features. Similarity measures and cluster analyses are performed to automatically group patients. Machine learning and data mining methods are applied to analyze the ultrasound images and parameters from mULM in order to extract clinically relevant information. The goal of study part B is to predict the response of patients with breast cancer to chemotherapy, and in study part C, to differentiate between benign and malignant breast masses in 2D and 3D. Parameters such as intensity, shape, or texture are extracted from conventional ultrasound images and combined with functional and structural parameters from the mULM analysis (e.g., tumor vascular architecture, blood flow profiles). The individual parameters and their combinations are evaluated for their predictive value. Various statistical methods are used to evaluate and select the parameters (or combinations thereof), including ANOVA, the Friedman test, and machine learning methods such as LASSO, gradient boosting, deep neural networks, and support vector machines. Thus, this project will contribute to the clinical translation of multiparametric high-resolution ultrasound and pave the way for ultrasound-based radiomics analysis. This holds the potential to advance noninvasive ultrasound diagnostics of the breast, thereby reducing the number—and thus the burden—of biopsies, and to predict response to chemotherapy in order to promptly replace ineffective treatment regimens.

Objective

The goal of this project is to adapt mULM for clinical use in order to investigate, in an exploratory patient study, the differentiation between benign and malignant breast cancer tumours as well as the prediction and monitoring of breast cancer responses to neoadjuvant chemotherapy.

Planning
Study design: Single-centre, prospective, diagnostic study

Study duration:
Total duration: 3.5 years: April 1, 2021 – October 1, 2024
Recruitment period: 38 months: April 1, 2021 – June 1, 2024

Study Population:

Study part A:	Female patients with primary breast cancer
Study part B:	Female patients with primary breast cancer who are undergoing neoadjuvant chemotherapy.
Study part C:	Patients with a high degree of suspicion of primary breast cancer

Participation by study participants of childbearing age is generally permitted; in cases of uncertainty, menopausal status will be determined through laboratory testing in accordance with national guidelines. The importance of effective contraception is emphasized during the chemotherapy counseling session, and immediate exclusion from the study will occur if a pregnancy is confirmed.

Planned number of cases:
Number of patients: 80
The statistical analysis was conducted in collaboration with the Institute for Medical Statistics (Stephanie Wied from the Institute for Medical Statistics, using nQuery software). For the exploratory study arm A, formal sample size planning is not possible. Based on and our preliminary work, we expect sufficient statistical power with 16 patients [5–9]. The calculations for study arms B and C were performed using a significance level of 5%, and a two-sided confidence interval was assumed. The calculation was based on the assumptions that, under neoadjuvant chemotherapy, 30% of patients will respond fully to the therapy (study part B), and that a 50% rate of malignancy can be assumed for highly suspicious tumours to be biopsied (study part C). With an expected sensitivity and specificity of 0.75, a permitted deviation of 0.25, and a permitted confidence interval width of 0.5, the sample size is 40 patients in study arm B and 24 patients in study arm C.

Inclusion criteria:

1. Patients with histologically confirmed primary breast cancer, including all intrinsic subtypes (Study Arms A and B), and patients with a high degree of suspicion of primary breast cancer (Study Arm C)
2. Treatment with neoadjuvant chemotherapy (Study Arms A and B)
3. Written informed consent
4. Individuals who are legally competent and mentally capable of following the instructions of the study staff

Exclusion criteria:

1. Minors
2. Hypersensitivity/allergy to ultrasound contrast agents or their components: sulfur hexafluoride, macrogol 4000, colfosceryl stearate, 1-(1,2-dipalmitoyl-3-sn-phosphatidyl)glycerol sodium salt, palmitic acid
3. Right-left shunt
4. Signs of cardiovascular instability
5. Acute endocarditis
6. Artificial heart valves
7. Acute systemic inflammation and/or sepsis
8. Overactive coagulation status and/or recent thromboembolism

9. End-stage kidney or liver disease
10. Severe pulmonary hypertension (pulmonary artery pressure > 90 mmHg)
11. Uncontrolled systemic hypertension
12. Acute respiratory distress syndrome
13. Pregnancy
14. Individuals who are institutionalized pursuant to an administrative or judicial order
15. Individuals who are in a relationship of dependence or employment with the sponsor or investigator
16. Alcohol and drug abuse
17. Anticipated non-compliance

Study protocol:

If a patient is eligible to participate in the study based on the inclusion and exclusion criteria, she is enrolled. The following data are collected during the screening visit: age, height, weight, smoking status, cardiac function, pulmonary status, complete blood count, and laboratory chemistry (according to the standards of the Department of Gynecology and Obstetrics at UK Aachen), as well as current medications. In addition, in all three study arms, following completion of the diagnostic workup, a histological examination is performed on the remaining tumour tissue archived at the Institute of Pathology, which is collected from the patients in accordance with guidelines during their care.

Study part A:

This study is conducted as part of the initial evaluation of patients with primary breast cancer. The clinical procedure follows the current guideline-compliant protocols of the Department of Gynecology and Obstetrics at Uniklinik RWTH Aachen. According to the guidelines, ultrasound imaging, along with mammography and clinical examination, is considered a baseline examination for the diagnostic evaluation of abnormal breast findings. During ultrasound imaging (Toshiba/Canon Aplio 500 system in 2D), the size, location, and structure of the tumour are assessed using B-mode ultrasound, elastography, and contrast-enhanced ultrasound. The study-relevant part of this procedure is the post-processing analysis of the contrast-enhanced ultrasound. To ensure optimal evaluation, the optimal dose is determined by administering the ultrasound contrast agent at two concentrations. If multiple lesions are present, all lesions are measured. The contrast-enhanced examination is performed on the largest lesion. A bolus of contrast agent is administered intravenously, and perfusion measurement within the tumour is performed on a representative cross-section. To ensure optimal visualization of the vessels, patients receive two doses of contrast agent at 0.0075 ml/kg body weight (BW) and 0.015 ml/kg BW. This total dose falls within the range of clinical practice and is below the maximum recommended dose of 0.03 ml/kg BW. The repeated administration of different concentrations corresponds to clinical practice, where repeated injections at varying concentrations are also used to optimally assess a lesion. A pause of approximately 15 minutes is kept between individual contrast agent injections, after which no microbubbles are circulating in the blood. mULM is based on the analysis of the recorded ultrasound data (Figures 1 and 2). The exact ultrasound examination procedure for study part A is outlined in SOP A. In addition, the remaining tumour tissue samples from the patients, which are archived at the Institute of Pathology and collected as part of routine clinical care, are examined histologically as part of the study after the pathological diagnosis has been completed. This includes tissue from the biopsy performed prior to the patients' enrolment in the study and tissue removed during surgical resection of the tumour.

Study part B:

This assessment takes place as part of the clinical evaluation of patients with primary breast cancer who are scheduled for neoadjuvant chemotherapy. The clinical procedure is conducted in accordance with the current guideline-compliant protocols of the Department of Gynecology and Obstetrics at Uniklinik RWTH Aachen. According to the guidelines, ultrasound is the standard clinical procedure for assessing tumour size during the course of treatment. As part of the study, non-contrast-enhanced diagnostic ultrasound (B-mode and elastography) is initially performed to assess the size, location, and structure of the tumour. The ultrasound examinations are performed in 2D using the Toshiba/Canon Aplio 500 system. If multiple lesions are present, all lesions are measured. The contrast-enhanced examination is performed to assess tumour perfusion in the largest lesion. A bolus of contrast agent is administered intravenously, and a perfusion measurement is performed within the tumour

at a representative cross-section. The dosage is based on the optimal dosage determined in study part A. This is followed by the infusion of the chemotherapeutic agent. After completion of neoadjuvant chemotherapy, surgical resection of the tumour and microscopic examination of the tissue are performed in accordance with guidelines (Figures 3 and 4). In addition to the resected specimens, the patients' biopsies, which were performed prior to enrolment in the study, are also analysed histologically. The exact ultrasound examination procedure for study part B is outlined in SOP B.

Study part C:
This examination takes place as part of the clinical evaluation of patients for the diagnostic evaluation of an abnormal breast finding. The diagnostic procedure is conducted in accordance with the current guidelines-compliant protocols of the Department of Gynecology and Obstetrics at Uniklinik RWTH Aachen. According to the guidelines, ultrasound imaging, along with mammography and clinical examination, is considered a baseline examination for the diagnostic assessment of abnormal breast findings. These findings will subsequently be histologically evaluated via biopsy. As part of the study, the ultrasound examination will initially consist of non-contrast-enhanced diagnostic ultrasound (B-mode and elastography) to document the location, size, and structure of the tumour. Ultrasound examinations are performed in 2D and 3D using the GE Volusion E10 or Philips Epiq 7/Elite. If multiple lesions are present, all lesions are measured. The contrast-enhanced examination is performed on the largest lesion. A bolus of contrast agent is administered intravenously, and a perfusion measurement is performed within the tumour at a representative cross-section. The dosage is based on the optimal dosage determined in study part A. The mULM is based on the analysis of the image data recorded during the examination. This is followed by a biopsy for histological evaluation of the tumours, during which the tumour grade, hormone receptor and HER2/neu status, proliferation rate, and various vascular parameters (e.g., vascular density) are assessed (Figures 5 and 6). In addition, if a patient's tumour is surgically resected, the resected specimen is also examined histologically. The exact ultrasound examination procedure for study part C is outlined in SOP C.

Tissue analyses (all three study parts):
As part of the study, histological examinations are performed on the tissue samples collected from the participants during biopsy and surgical resection. These are absolutely necessary to validate the results of the mULM examination and to verify their accuracy. These tissue samples are part of routine clinical practice and are not collected specifically for the study. The tissue examinations conducted as part of this study are carried out in cooperation with the Institute of Pathology at RWTH Aachen University Hospital. The focus of the tissue examinations is on the analysis of vascular structures, specifically involving staining of endothelial cells (CD31) and smooth muscle cells (SMA). In addition, a database search is conducted regarding the histological findings (e.g., grade, hormone receptor and HER2/neu status, proliferation density, vascular density). If necessary, additional standard staining are performed (e.g., HE, hormone receptors, HER2/neu receptors, proliferation rate).
For the analysis of the tissue samples, these are pseudonymised (see Patient Identification), whereby the same pseudonym is used for assignment to the corresponding participant as for the contrast-enhanced ultrasound examinations. Thus, the tissue sample is assigned to the corresponding pseudonym, but not to the patient as an individual.
Both tissue samples are part of routine patient care and are not collected for the purposes of this study. These tissues consist of paraffin blocks that have been stored long-term at the Institute of Pathology; they were collected as part of routine clinical practice and are no longer needed for diagnostic purposes at the time of the study-related histological examination. Sufficient preserved material remains to allow for further examinations even outside the scope of the study. The tissue blocks will remain in the long-term archive of the Institute of Pathology at the Uniklinik RWTH Aachen, and the minimum archiving periods required by the German Cancer Society will be taken into account during the conduct of the study.

Study withdrawal:
A patient may be withdrawn from the study if
- the patient does not follow the instructions of the study staff, resulting in deviations from the study protocol (lack of compliance).
- inclusion and exclusion criteria are no longer met.

- The patient withdraws their informed consent.
- Other reasons, at the physician's discretion, preclude further participation in the study.

The entire study or parts of the study may be terminated if

- the safety, well-being, and privacy of the participating patients cannot be guaranteed.
- the study is not being conducted in accordance with the protocol.

Benefit-risk assessment

Potential benefits for the patient from participating

The use of mULM in breast cancer has not yet been studied. The diagnostic differentiation of tumour entities using mULM between benign and malignant, and between responders and non-responders offers the potential to optimize patient care. As a non-invasive method, mULM could reduce the need for invasive biopsies of lesions suspected of being tumours, thereby alleviating the burden on patients. Early detection of a tumour's lack of response to chemotherapy allows for a switch to effective therapies and eliminates the side effects of ineffective treatment. Patients should not expect any immediate benefits.

Potential side effects from the contrast agent

SonoVue® has been approved in the European Union (EU) since 2001 for ultrasound diagnostics to enhance the visibility of blood, primarily for the characterization of liver and breast tumours. The mULM examination is a post-processing analysis of the SonoVue® application.

The injection of the contrast agent may cause the following side effects:

Occasionally (1 to 10 patients out of 1,000 (0.1–1%))

- Headaches, paresthesia (e.g., tingling), dizziness, taste disturbances
- Skin redness
- Sore throat (pharyngitis)
- Nausea, abdominal pain
- Itching, rash
- Back pain
- Chest discomfort, reactions at the injection site, sensation of heat
- Elevated blood sugar levels

Rare (1 to 10 out of 10,000 treated patients (0.01–0.1%))

- Hypersensitivity (signs include, among others, skin erythema, bradycardia, hypotension, dyspnea, loss of consciousness, cardiac arrest/cardiovascular arrest, anaphylactic reaction, anaphylactic shock)
- Insomnia
- Sinus headache (headache originating in the sinuses)
- Blurred vision
- Low blood pressure
- Chest pain, pain, fatigue

Unknown

- Vasovagal reaction
- Myocardial infarction, myocardial ischemia
- Vomiting

SonoVue® must not be used in patients with a known right-left shunt, signs of cardiovascular instability, acute endocarditis, artificial heart valves, acute systemic inflammation and/or sepsis, hypercoagulable state and/or recent thromboembolism, end-stage renal or hepatic disease, severe pulmonary hypertension (pulmonary artery pressure > 90 mmHg), uncontrolled systemic hypertension, or acute respiratory distress syndrome, or in cases of hypersensitivity to ultrasound contrast agents or their components—sulfur hexafluoride, macrogol 4000,

colfosceryl stearate, or 1-(1,2-dipalmitoyl-3-sn-phosphatidyl)glycerol sodium salt of palmitic acid. In very rare cases, fatal reactions have been reported that were temporally associated (within 3 weeks) with the use of SonoVue®. Most of these patients had a high baseline risk of severe cardiac complications, which may have contributed to the fatal outcome.

Potential side effects of ultrasound

There is no information available on the long-term effects of contrast-enhanced ultrasound in patients with cancer. In studies in which contrast-enhanced diagnostic and therapy-supporting ultrasound was performed in patients with pancreatic cancer following chemotherapy, patients did not experience any additional discomfort from the contrast-enhanced diagnostic and therapy-supporting ultrasound compared to chemotherapy alone [10,11].

Potential side effects from the indwelling venous catheter

Misplacement of the needle, thrombophlebitis, post-procedural bleeding, or hematomas may occur, as well as nerve irritation at the puncture site. In very rare cases, thrombosis, extravasation, thromboembolism, sepsis, vascular injury, or nerve injury with permanent damage in the area supplied by the affected nerves may also occur.

COVID-19-related study risks

The study takes place during the patients' routine hospital stay at Uniklinik RWTH Aachen. Therefore, they do not have to travel any additional distances that would increase their risk of SARS-CoV-2 infection. To minimize the risk of infection during the examination, all participants are required to wear a facemask. Whenever possible, a minimum distance of 1.5 meters will be maintained between all individuals. The number of people present in the examination room is also kept to a minimum. Furthermore, all surfaces that patients come into contact with are disinfected before and after the examination. These measures reduce the risk of SARS-CoV-2 transmission as much as possible, meaning that the risk of contracting COVID-19 is considered low.

Data management

Data is entered into an electronic case report form. The principal investigator retains patient records and original data for as long as possible and in accordance with local retention standards. The principal investigator retains study documents in accordance with the ICH-GCP (International Council for Harmonisation of Technical Requirements for Pharmaceuticals for Human Use Good Clinical Practice) guidelines. The principal investigator/study site takes measures to prevent the accidental or premature destruction of the documents. Study documents must not be destroyed before the retention period required of personnel responsible for conducting the study has expired.

Data quality

This study will be designed and conducted in a manner that ensures the use of standardized terminology and procedures and the collection of accurate, consistent, and reliable data. All requirements for the planning, conduct, and completion of a GCP-compliant clinical trial are taken into account to enable the collection of valid, objective, and reliable data, while adhering to all ethical and legal provisions and high-quality standards to ensure the well-being of study participants.

AE & SAE

Adverse events (AE) and severe adverse events (SAE) are documented, evaluated, and reported in accordance with GCP.

Patient identification

A patient identification list is maintained. This list links patients' personal data to their pseudonyms and is stored exclusively at the clinic under GCP-compliant conditions. Copies may not be made at any time.

Data protection

It is ensured that patient-related data and tissue samples are never disclosed to third parties. Analyses are conducted exclusively using pseudonymised or anonymised data. Results of analyses are published in anonymised form, so that it is no longer possible to link data to individual patients.

The data is entered into a secure database using a personal identification number. This database contains no information that would allow clinical data to be linked to a specific individual. A separate database containing identification data (pseudonymisation information) for the registered patients contains no clinical data and does not allow patient data to be linked to the identification number in the clinical database. Only a limited, specifically documented group of database administrators can link patient identification data to clinical data. The data is stored on password-protected server drives. The relevant data protection regulations under the Federal Data Protection Act (Bundesdatenschutzgesetz) are considered at all times.
Upon completion of the study, proper storage and management of all study documents will be ensured in accordance with legal requirements for up to ten years.

Publication
The study will be registered at ClinicalTrials.gov. The study results will be published in at least one scientific article. The ethics committee will be informed of the study results.

Insurance
No additional patient insurance was obtained. The Uniklinik RWTH Aachen and its staff members participating in the study are covered by liability insurance in the event that you suffer harm due to their negligence. No travel accident insurance was obtained.

Study schedule

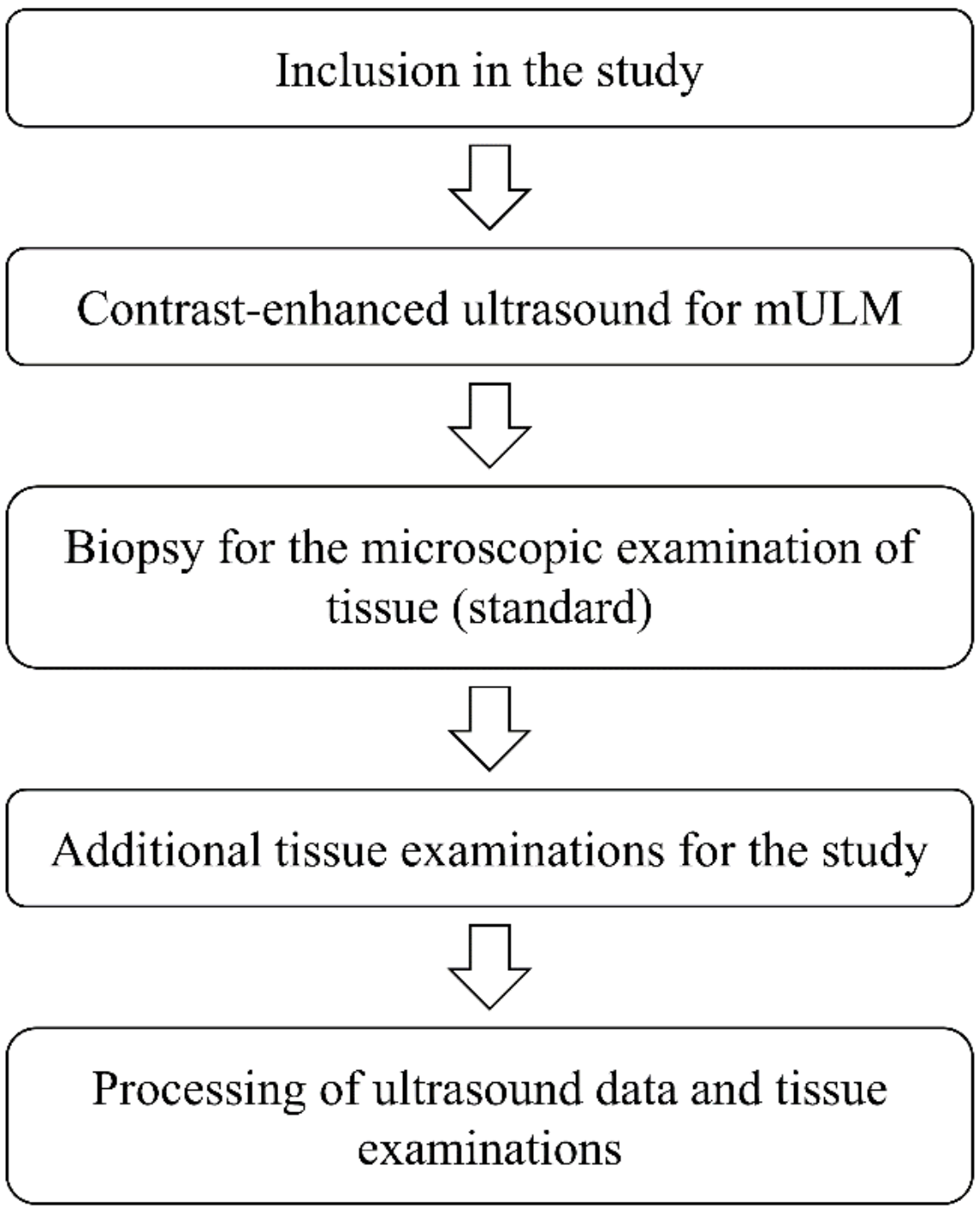

Figure 1. Research plan for study part A

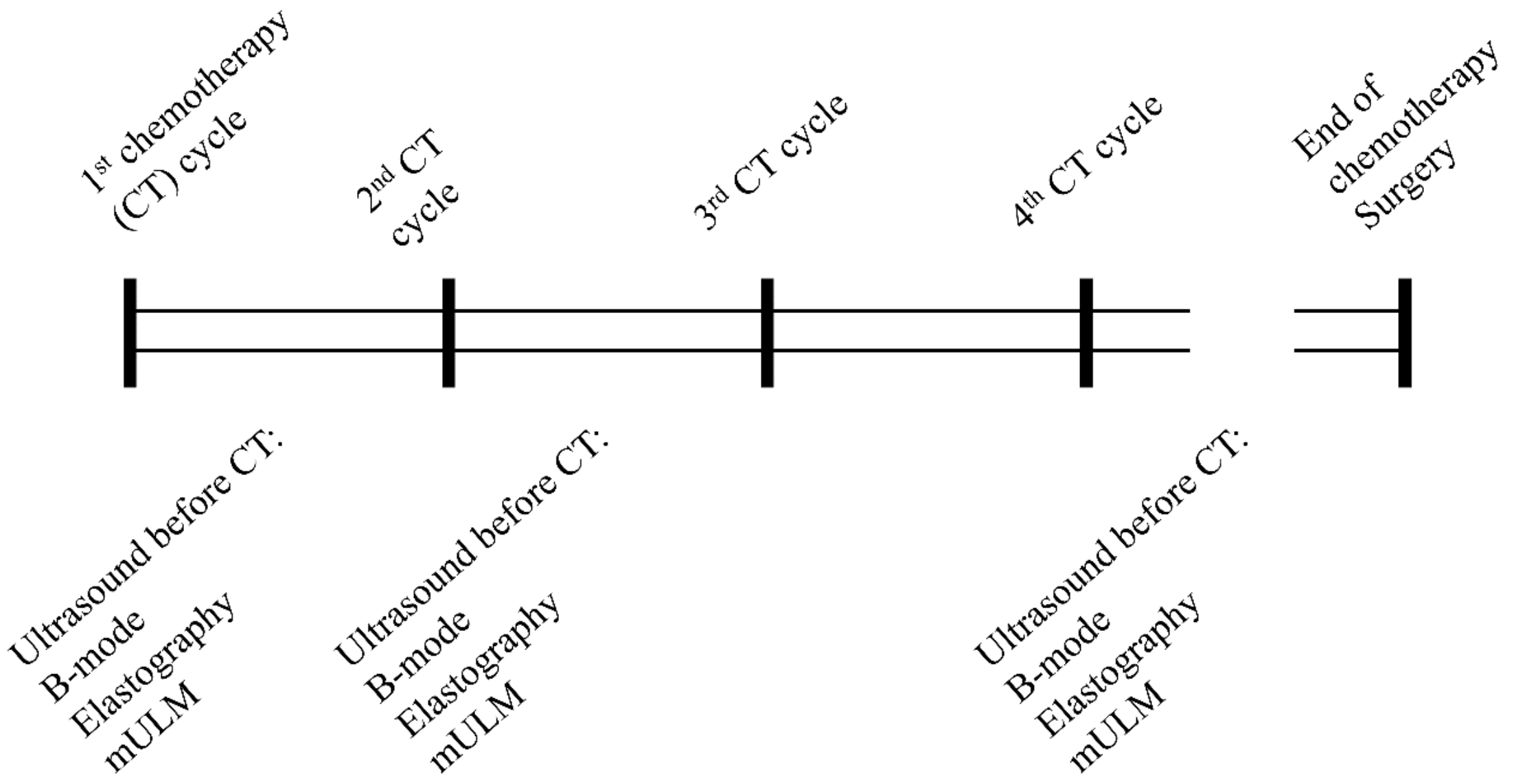

Figure 2. Workflow of study part A

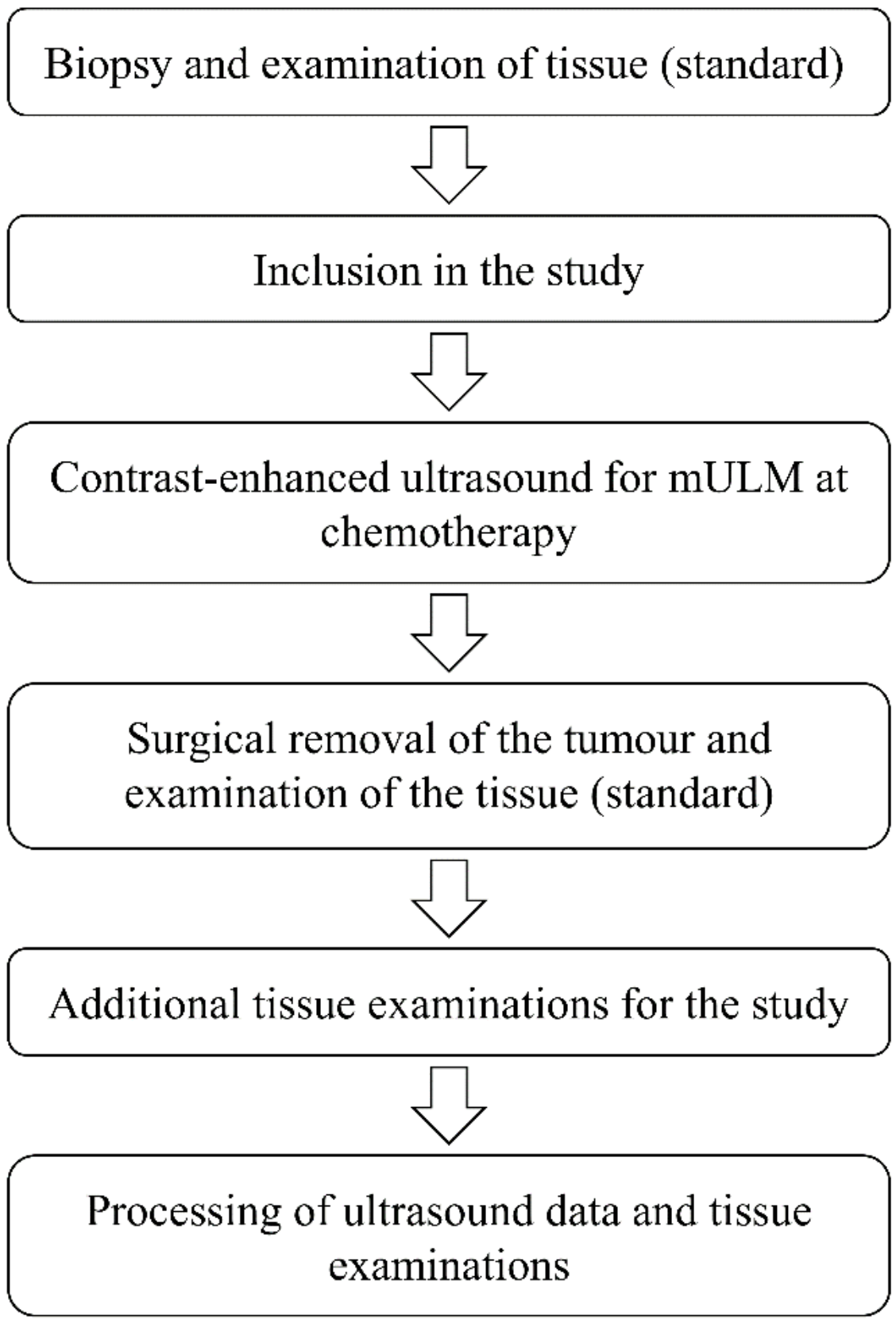

Figure 3. Research plan for study part B

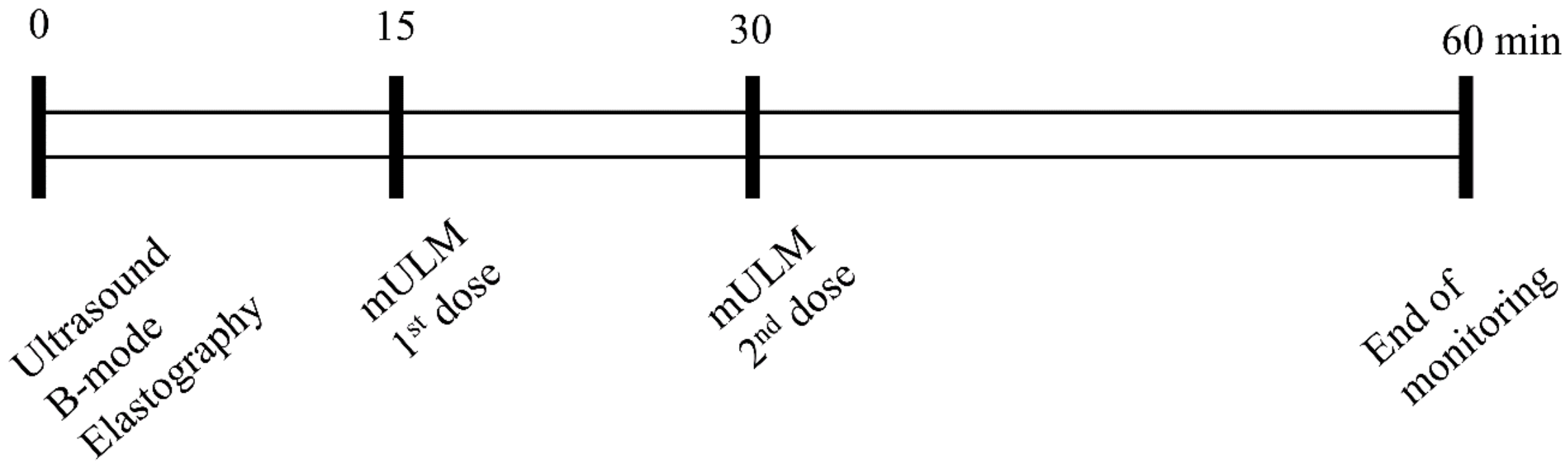

Figure 4. Workflow of study part B

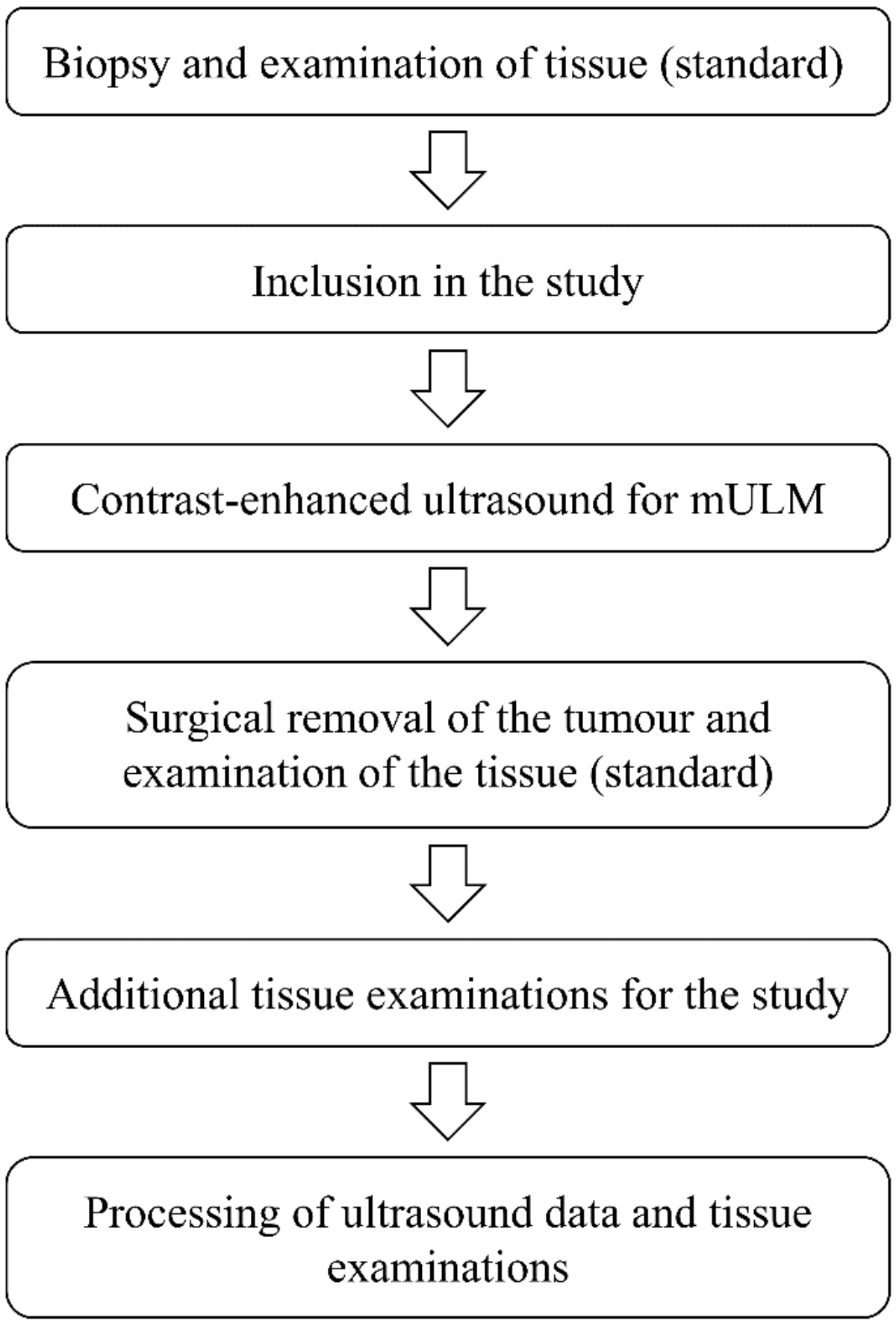

Figure 5. Research plan for study part C

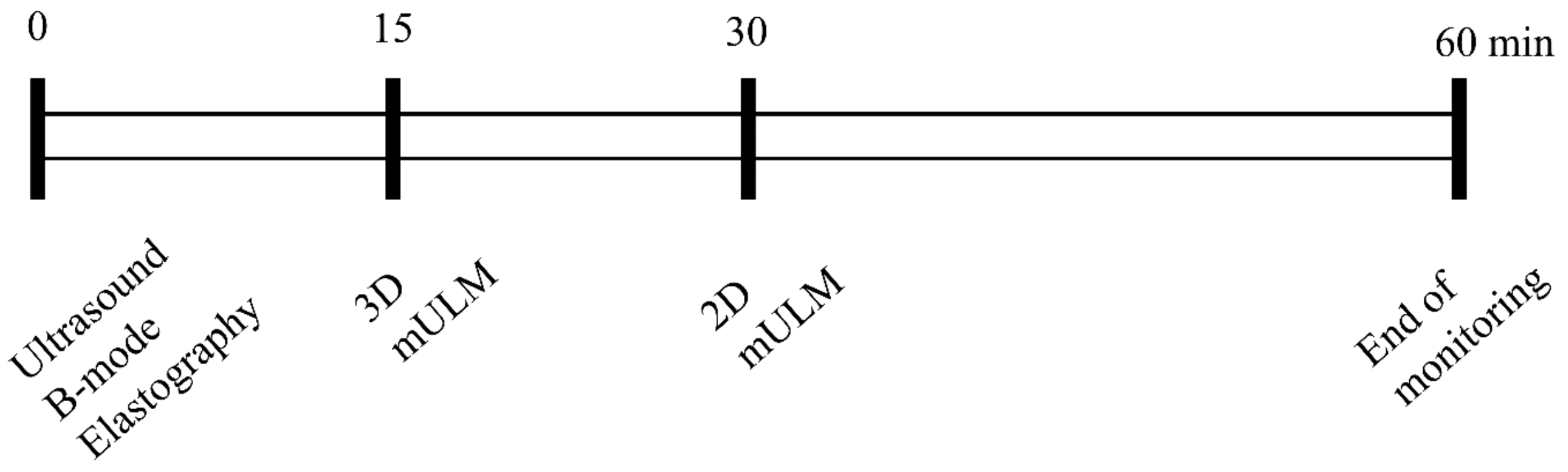

Figure 6. Workflow of study part C

Signatures

I have read the study protocol thoroughly and verified its accuracy and completeness. I understand the prerequisites and conditions of this study and hereby affirm that I will conduct the study in accordance with this protocol, the Declaration of Helsinki, and applicable legal provisions (§ 15 BOÄ, BDSG).

<u>I also agree to</u>

- await approval from the relevant ethics committee before enrolling study participants in this study.
- carry out study-related procedures only after obtaining the study participant’s written consent.
- submit any changes to the study protocol as an amendment to the relevant ethics committee for approval.
- ensure access to all original data, records, and medical files at all times
- treat study information as confidential.

By signing this document, I acknowledge receipt of the study protocol.

References

[1] Zhao H, Xu R, Ouyang Q, Chen L, Dong B, Huihua Y. Contrast-enhanced ultrasound is helpful in the differentiation of malignant and benign breast lesions. Eur J Radiol. 2010; 73: 288-293.

[2] Wan CF, Du J, Fang H, Li FH, Zhu JS, Liu Q. Enhancement Patterns and Parameters of Breast Cancers on Contrast-Enhanced Ultrasound: Correlation with Prognostic Factors. Radiology 2012; 262: 450–459.

[3] Saracco A, Szabó BK, Tánczos E, Bergh J, Hatschek T. Contrast-enhanced ultrasound (CEUS) in assessing early response among patients with invasive breast cancer undergoing neoadjuvant chemotherapy. Acta Radiologica 2017; 58: 394-402.

[4] Luo J, Chen JD, Chen Q, Yue LX, Zhou G, Lan C, Li Y, Wu CH, Lu JQ. Contrast-enhanced ultrasound improved the performance of the Breast Imaging Reporting and Data System (BI-RADS) evaluation of critical breast lesions. World J Radiol 8 2016; 8: 610–617.

[5] Ackermann D, Schmitz G. Detection and tracking of multiple microbubbles in ultrasound B-mode images. IEEE Transactions on Ultrasonics, Ferroelectrics, and Frequency Control. 2016; 63(1):72-82.

[6] Dencks S, Piepenbrock M, Opacic T, Krauspe B, Stickeler E, Kiessling F, Schmitz G. Clinical pilot application of super-resolution ultrasound imaging in breast cancer. IEEE Trans Ultrason, Ferroelectr, Freq Control. 2019;66(3):517-26.

[7] Opacic T, Dencks S, Theek B, Piepenbrock M, Ackermann D, Rix A, Lammers T, Stickeler E, Delorme S, Schmitz G, Kiessling F. Motion-model ultrasound localization microscopy for preclinical and clinical multiparametric tumor characterization. Nature Communications. 2018;9(1):1527.

[8] Theek B, Opacic T, Mockel D, Schmitz G, Lammers T, Kiessling F. Automated generation of reliable blood velocity parameter maps from contrast-enhanced ultrasound data. Contrast Media Mol Imaging. 2017;2017:2098324.

[9] Theek B, Opacic T, Magnuska Z, Lammers T, Kiessling F. Radiomic analysis of contrast-enhanced ultrasound data. Sci Rep. 2018;8(1):11359.

[10] Dimcevski G, Kotopoulis S, Bjåne T, Hoem D, Schjött J, Gjertsen BT, Biermann M, Molven A, Sorbye H, McCormac E, Postema M, and Gilja OH. Ultrasound and microbubble-enhanced treatment of inoperable pancreatic adenocarcinoma. J Clin Oncol 2016;34.

[11] Kotopoulis S, Dimcevski G, Gilja OH, Hoem D, and Postema M. Treatment of human pancreatic cancer using combined ultrasound, microbubbles, and gemcitabine: a clinical case study. Med Phys. 2013;40:072902.
